# Successful coordination emerges from aligned self-predictions in a dynamic motor interaction

**DOI:** 10.1038/s44271-026-00523-7

**Published:** 2026-08-20

**Authors:** Prasetia Utama Putra, Fumihiro Kano

**Affiliations:** 1 Centre for the Advanced Study of Collective Behaviour, University of Konstanz, Konstanz, Germany; 2 Max-Planck Institute of Animal Behavior, Konstanz, Germany; 3 Present Address: Institute for Advanced Study, Kyushu University, Fukuoka, Japan

**Keywords:** Human behaviour, Computational science, Perception

## Abstract

A central challenge in understanding joint action is explaining how individuals achieve successful coordination in dynamic, real-world settings. Although coordination is thought to depend on anticipating future events, including the actions of others, the precise contribution of such predictive processing remains unclear, since most existing evidence comes from simplified laboratory tasks that infer anticipation indirectly from reaction times. To address this gap, we studied 70 participants (forming 64 pairs) during solo and joint sessions of a turn-taking ball-hitting task while recording multimodal behavioral, physiological, demographic, and social measures. We found that successful coordination was most strongly associated with anticipation of one’s own action outcomes, outperforming physiological synchrony, motor behavior, demographic characteristics, and social closeness. Partners whose predictions of their own action outcomes were more closely aligned coordinated better, likely because each behaved in ways the other implicitly expected. A Bayesian generative model further showed that well-coordinated pairs relied more heavily on prior expectations than on incoming sensory evidence when generating predictions during joint action. These results suggest that efficient coordination in dynamic real-world tasks emerges primarily when individuals’ internal models for predicting their own actions are well aligned across partners. Our quantitative multimodal approach provides a framework for disentangling the contributions of predictive, physiological, motor, and social factors to coordination in naturalistic interactions.

## Abstract (web summary)

Combining eye-movement, body-kinematics, heart-rate, and individual-characteristic data with machine learning, this study shows that coordination during dynamic motor interaction reflects how similarly partners predict their own actions.

## Introduction

Humans excel at cooperating across scales^1–3^, an ability that often depends on effective coordination among individuals. Coordination, defined as the spatiotemporal alignment of actions to achieve shared goals or sustain interaction^4,5^, has been studied across cognitive psychology^5^, neuroscience^6^, motor control^7^, computational modeling^8^, and artificial intelligence^9^. These studies have examined coordination in tasks ranging from lifting a plank^10^ to musical duet performance^11,12^, to human-robot collaboration^13–15^. While some forms of coordination emerge spontaneously from local interactions, such as synchrony in animal groups^16^, phase-locking between rocking chairs^17^, or human-machine herding^18^, everyday human coordination is considerably more perceptually and cognitively demanding, requiring continuous integration of perceptual, motor, and predictive processes. Understanding these mechanisms is important not only for theories of social interaction, but also for applications in collaborative robotics^19^, sports training^20^, and teamwork in education and healthcare^21^.

Several complementary theoretical frameworks propose that predictive processing plays a central role in successful coordination. Minimal architectures for joint action emphasize shared task representations and the ability to monitor and predict a partner’s behavior^22,23^. Computational accounts extend principles from motor control to social interaction, proposing that interacting with others relies on predictive and control mechanisms similar to those used for one’s own bodily movements^7,24,25^. Likewise, the Predictive Joint-Action Model^26^ proposes that goal representations, action planning, and sensory processing interact hierarchically to minimize prediction errors. From the perspective of the free-energy principle^27,28^, coordination can be understood as the continual updating of internal generative models to minimize surprise by balancing prior expectations with incoming sensory evidence. Together, these frameworks suggest that successful coordination depends on predictive alignment between interacting individuals.

Although these theoretical models are supported by empirical work, most experimental paradigms rely on simplified tasks such as finger tapping^29,30^, manipulating objects to produce sounds^25^, bouncing a ball on a drum^31^, or balancing a tray^32^. These paradigms offer experimental control but capture less of what happens in naturalistic settings, where individuals must continuously adapt under uncertainty about future events^33–35^. Everyday joint action involves rich verbal and nonverbal sensorimotor communication, with individuals continuously monitoring and adapting to one another across multiple sensory modalities and timescales^33,34^. Such interactions are further complicated by perceptual uncertainty, environmental noise, and the inherent unpredictability of others’ behavior, factors that are often minimized or absent in simplified tasks^35^. As a result, the contribution of predictive processing to successful coordination in dynamic, naturalistic interactions remains unclear.

Answering this question is challenging because successful coordination can emerge from multiple interacting mechanisms. Demographic, physical, physiological, and relational variables, including age, gender, body morphology, movement dynamics, physiological synchrony, and social closeness, have all been linked to coordination performance^10,30,31,36–38^. Consequently, even if prediction contributes to successful coordination, its relative importance remains difficult to determine.

A further challenge concerns the mechanisms that support successful coordination. At one extreme, coordination may emerge without rich predictive representations. In highly rhythmic tasks, coordinated behavior can arise through mutual coupling between interacting agents, leading to stable phase-locked patterns of behavior without explicit anticipation of future actions^17,39,40^. However, it remains unclear whether such mechanisms are sufficient in naturalistic interactions, where coordination often unfolds under irregular timing, changing environmental conditions, and uncertainty about future events.

When prediction is required, one possibility is that anticipation is generated by extrapolating currently available sensory information^41,42^. For example, a player may track a ball’s trajectory to estimate its future position^43,44^, or a musician may predict the next beat by extending an ongoing rhythmic pattern^45^. Prediction of a partner’s actions may likewise depend on ongoing sensory information, but often involves continuously integrating information generated by another individual during the interaction^46,47^. A second possibility is that anticipation relies on internal models that generate expectations about upcoming actions and their sensory consequences. Such models are well established in motor control, where the nervous system predicts the outcomes of self-generated actions before sensory feedback becomes available^48^. Extended to social interaction, these same mechanisms may support anticipation of a partner’s behavior through simulation or internal representation of their actions^7,12,49,50^. Whether successful coordination depends primarily on online sensory extrapolation, internal models, or some combination of the two remains unclear.

Another key challenge concerns the target of prediction. Even if prediction plays a central role in coordination, it remains unclear what individuals are predicting^22^. Most coordination paradigms assess performance through joint outcomes (e.g., refs. ^49,51^), making it difficult to determine whether successful coordination depends on anticipating a partner’s actions, the consequences of one’s own actions, or both. One notable exception comes from musical duet performance, where pianists synchronized more accurately with recordings of their own previous performances than with recordings by other pianists, despite comparable temporal structure across recordings^12^. This finding suggests that successful coordination may depend not only on predicting a partner’s behavior but also on predicting the sensory consequences of one’s own actions. However, the relative contributions of self-prediction and partner-prediction remain largely unexplored.

To address these challenges, we developed a dynamic turn-taking ball-hitting task that combines continuous uncertainty about future events with alternating contributions from self and partner. We used anticipatory eye movements as a window onto prediction, as gaze typically shifts toward expected future events before they are confirmed by sensory feedback^52^. Anticipatory eye movements have been extensively used to study prediction in fast-paced ball sports, where performers direct their gaze toward future task-relevant locations before the ball arrives^53–57^. In a recent solo ball-hitting task, Putra et al.^58^ showed that the timing and precision of anticipatory saccades to future bounce locations predicted successful performance, indicating that these eye movements provide a quantitative measure of predictive processing during dynamic interception tasks. By extending this approach to a dyadic setting, our paradigm allows us to distinguish predictions following one’s own actions from those following a partner’s actions while participants coordinate in real time.

Coordination success was defined as performing better together than expected from individual skill alone. We estimated expected performance as the average of the two partners’ individual-task scores. Pairs whose joint performance exceeded this value were considered successful, indicating that their coordination produced gains beyond the sum of each person’s solo ability. To estimate the relative contribution of candidate predictors, we applied machine-learning techniques, including SHapley Additive exPlanations (SHAP)^59^. In addition, Bayesian generative models derived from the free-energy principle^27^ provided a formal framework for evaluating how prior expectations and incoming sensory evidence contribute to predictive processing during joint action.

We addressed three central hypotheses. First, based on previous findings linking anticipatory gaze and motor control to successful performance in ball-interception tasks^53,58^, we hypothesized that anticipatory gaze behavior and motor performance measured during individual-action tasks would predict coordination success during joint action. Second, based on studies showing that skilled coordination depends on predictive adaptation between interacting individuals^12,25^, we hypothesized that well-coordinated pairs would differ from less-coordinated pairs in both anticipatory gaze behavior and movement dynamics during interaction. Third, based on predictive-processing accounts of joint action^26,27^, we hypothesized that anticipatory gaze error during successful coordination would depend more strongly on internal-model-based prediction than on online tracking of current sensory evidence alone.

Our paradigm also allowed us to address an additional question for which we did not make a strong prediction: whether successful coordination is more closely associated with anticipating the consequences of one’s own actions or those of a partner. Although both forms of prediction have been implicated in joint action^5,7,12,60^, they are typically difficult to disentangle because they contribute to the same joint outcome. In our task, however, we could compare anticipatory gaze following self-generated hits with anticipatory gaze following partner-generated hits, which offered insight into these potentially distinct forms of prediction during ongoing coordination.

## Methods

### Participants and datasets

A total of 91 participants (36 female, 54 male, and one non-binary; mean age =  25.29 years, SD = 7.09, range = 19–45) were recruited and received monetary compensation (see Supplementary Information for justification of sample size). All participants were free of physical disability. Those with hyperopia or myopia used corrective lenses with their Tobii Glasses. Five participants were members of a ping-pong club; the rest had minimal or casual experience. The study was approved by the Ethics Committee of the University of Konstanz (No. 39/2022), and data collection occurred between November 2022 and March 2023. Prior to participation in the experimental sessions, all participants provided written informed consent, and only those who had done so were enrolled in the study. This study was not preregistered. A part of the dataset used in this study, specifically from the individual-action task, was previously reported in^58^.

### Experimental procedures

During the experiments, participants wore a motion-capture suit, Tobii Glasses 3, and an ECG (electrocardiogram) sensor. The motion-capture system comprised 13 Vicon Vero cameras and one Vicon Vantage camera, all sampling at 100 Hz. The Tobii Glasses recorded gaze data at 50 Hz, while the ECG sensor recorded inter-beat intervals at approximately 1–2 Hz (see S1 and S2). To synchronize the devices, transistor-transistor logic pulses were sent to the glasses’ recording unit, and ECG acquisition was triggered concurrently with the start of the Vicon motion-capture recording.

We also tracked the position and motion of the racket, ball, table, and wall. Reflective markers were placed on the table, wall, and racket, and the ball was coated with reflective powder for tracking. The racket was 24.6 cm long, 15 cm wide, and weighed 142.2 g. The ball had a 4.5 cm diameter and weighed 4.04 g. Static and functional calibration for pose estimation was conducted before the experiment, and eye-tracker calibration was repeated before each task.

Each session included three participants, or two if one canceled, who completed both individual and joint-action tasks (see Supplementary Information for details). Before beginning, participants read an information sheet and watched an instructional video. In the individual-action task, participants hit the ball against the wall for approximately 3 min. In the joint-action task, two participants took alternating turns hitting the ball in a coordinated manner for 5 to 6 min. The order of tasks was counterbalanced across participants to control for order effects. Sessions with two participants involved a single joint-action task, producing one dyad. Sessions with three participants involved three joint-action tasks covering all pairwise combinations (AB, AC, and BC), so that each participant completed the joint task twice with two different partners. The experiment was preceded by a brief warm-up of approximately 1 min that allowed participants to familiarize themselves with the equipment and the instructions.

After the tasks, participants completed a questionnaire covering demographics (age, gender, height, and weight), self-assessed table tennis skills, their relationship with their partner, and evaluations of each other’s performance. Data on ethnicity and cultural background were not collected in this study.

### Data preprocessing

Before computing predictors, we performed three preparatory steps: data preprocessing, verification of device synchronization, and a sanity check of the recorded data.

Preprocessing involved noise filtering and gap-filling for both eye-movement and motion capture data. For motion capture, the cleaning process consisted of three main steps. First, we applied a Woltring filter^61^ with a mean-squared-error mode and a smoothing level of 20. The filtered data were then exported as CSV files. Second, we removed segments and trajectories affected by marker swapping, based on marker distance and velocity patterns (see Supplementary Information). Third, any missing data were linearly interpolated. To reduce noise and emphasize trends, we smoothed the ball trajectory using a moving average with a 3-frame window. No filtering was applied to the eye-movement data. However, we up-sampled the eye-tracking signal to 100 Hz using linear interpolation to align it with the motion capture sampling rate.

Synchronization was double-checked by cross-referencing the timestamp of ball-wall contact events with the video recordings from the eye tracker.

Sanity checks were conducted by verifying that the number of recorded data points matched the expected count, calculated as the product of the recording duration and the device’s sampling rate.

Predictors were computed at the event level. An event was defined as a continuous sequence beginning with racket-ball contact (start of Phase 1), followed by the ball bouncing off the wall (end of Phase 1 and start of Phase 2), then bouncing on the table (end of Phase 2 and start of Phase 3), and ending with the next racket-ball contact (end of Phase 3). Events containing all three phases were classified as successful; otherwise, they were marked as failures. Phase detection was based on the ball’s proximity to task-relevant objects: Phase 1 was identified using the distance to the racket and wall, Phase 2 using the distance to the wall and table, and Phase 3 using the distance to the table and racket.

Data exclusion criteria included an eye-tracking quality of less than 65% and a total score of less than 55% in either individual-action or joint-action tasks. As a result, 9 out of 91 individual-action tasks and 15 out of 79 joint-action tasks were excluded. After applying these criteria, 70 participants contributing 64 valid dyads remained in the final dataset.

### Quantification of predictors

#### Rationale for predictors

We measured **eye movements**, in particular *anticipatory saccades*, because in fast-paced ball sports players direct their gaze toward bounce points or the ball’s future position well before contact^53–57,62^. When predictions deviate from the actual trajectory, players produce *corrective saccades* to adjust their gaze. Such anticipatory gaze shifts are critical for successful ball interception^58^. We also measured gaze behavior immediately before *contact*, specifically smooth pursuit and fixation (together referred to as *pursuit*), as tracking the ball during this phase has been shown to influence performance: individuals who maintain longer pursuit durations tend to perform better^63^.

We measured the timing of the forward swing prior to ball contact (**pre-contact swing**) and the alignment of gaze with the anticipated point of racket-ball contact (*gaze-racket distance*) to estimate the degree of motor planning^64^.

Because successful ball interception depends directly on racket-ball kinematics at **contact**^58,65,66^, we quantified impact kinematic variables, including *ball-racket angle*, *ball speed control* (the change in ball speed relative to racket speed), and *racket spatial control*, defined as the spatial position of ball contact relative to the wrist.

We measured similarity in **heart rate** variability (*LF/HF*), the ratio of low-frequency to high-frequency spectral power, commonly interpreted as an index of sympathetic-parasympathetic balance, because interpersonal physiological synchrony has been associated with shared attention, social cohesion, and cooperative success^36,67,68^.

We measured whole-body **movement intensity** (*motion energy*) because similarity in motion energy may reflect similar levels of physical effort during interaction^50,69^.

Finally, we measured individual and relational traits (**static** parameters) because individuals’ *skills*, demographic characteristics such as *gender*^37^ and *age*^31,37,38,70^, physical characteristics such as *height*^10^, and their *social relationship*^30^ can influence coordination.

#### Eye-movement

We measured two key eye movement behaviors: saccades and pursuit. Saccades are rapid, ballistic eye movements that shift the point of fixation, enabling the eyes to quickly move between points of interest^71^. In contrast, pursuit occurs when the eyes maintain a stable image of a moving target on the fovea^72^. Saccade detection was performed using a method established in a previous work^58^, as detailed below.

Saccades were identified using Duchowski’s 5-tap filter ([0, 1, 2, 3, 2, 1, 0])^73^, applied to the instantaneous visual angle between successive gaze-in-the-head vectors. A saccade was detected when the filtered velocity exceeded 40^∘^/s. The onset and offset were determined by identifying points where acceleration surpassed 15^∘^/s^2^.

Among detected saccades, anticipatory saccades were defined as those that followed the ball’s trajectory and exhibited the largest amplitude within the relevant phase. Saccades occurring after this anticipatory movement were labeled as corrective, while all remaining saccades were classified as normal and excluded from further analysis.

Pursuit episodes were identified when the gaze met four criteria: (1) the visual angle between the gaze and the ball was smaller than 10^∘^; (2) the velocity of the gaze-in-the-head exceeded 30^∘^/s; (3) the ratio between gaze velocity and ball velocity fell between 0.3 and 1.2; (4) the segment had to persist for at least 50 ms.

#### Pre-contact swing and contact

Pre-contact swing predictors included the swing onset and the gaze-racket distance that we found strongly correlated with individuals’ performance during the ball interception task^58^. The onset of the pre-contact swing was identified by detecting a peak in the acceleration profile of the distance between the racket and the wall. Gaze-racket distance was defined as the visual angle between the gaze position at the offset of pursuit and the racket position at the moment of contact.

Contact predictors included the ball-racket angle, racket spatial control, and ball speed control. The ball-racket angle was computed as the angle between the post-contact trajectories of the ball and racket, after subtracting the contact point from both vectors. Racket spatial control was calculated as the Euclidean distance between the wrist and the contact point, after subtracting the racket’s centroid to normalize for its spatial orientation. Ball speed control was quantified as the difference in ball speed before and after contact.

#### Heart rate and movement intensity

The low-to-high frequency (LF/HF) ratio was used as a heart rate measure. To account for initial preparation time, we excluded the first 30 s of RR-interval data (the time between two consecutive heartbeats) before calculating the LF/HF ratio (this procedure increased XGBoost performance, see Supplementary Information). The LF/HF values were then computed using the time-domain functions provided in the hrv-analysis library^74^.

Movement intensity was quantified using motion energy, defined as the mean spatial displacement in joint angles across consecutive frames. Whole-body motion energy was computed as the time-averaged motion energy across the upper body, lower body, and head segments.

#### Static characteristics

Static characteristics included skill, age, height, gender, and the social relationship with a partner during joint action. Skill was measured quantitatively as the number of successes during the individual-action tasks, while height was estimated as the mean translational position of the Y-axis from the individual’s Tobii segment of motion capture data. Additional measures included age, gender, self-rated performance, team-rated performance, perceived role as facilitator during joint action, and performance in the individual-action task administered after the experiment. Social relationship was rated on a scale from 1 (stranger) to 7 (very close).

### Analysis framework

#### Above- and below-expected groups

The categorization into above-expected and below-expected groups was derived using a Bayesian Gaussian Linear Model (GLM) (Eq. (1)). In this model, participants’ success scores in individual-action tasks served as a linear predictor of their performance in joint-action tasks:

1 $$\begin{array}{rcl}&&y \sim T(3,\mu ,\sigma )\hfill\\ &&\mu =\alpha +\beta * X\hfill\\ &&\alpha \sim N(0,5)\hfill\\ &&\beta \sim N(0,5)\hfill\\ &&\sigma \sim HalfCauchy(10)\end{array}$$

where *X* represents individuals’ success percentage in individual-action tasks, and *y* denotes pairs’ success percentage in joint-action tasks.

Model parameters were estimated using U-Turn Hamiltonian Monte Carlo sampling^75^, with hyperparameters detailed in Table 1. Based on the posterior estimates, we computed the predicted mean of joint-action performance. Teams whose scores exceeded the predicted mean were classified as above-expected, while those falling below it were classified as below-expected.

**Table 1:** Hyperparameters used to sample the model parameters vary for each model, taking into account the convergence of the estimation process

Parameters	Values (GLM)	Values (*T*-Test and generative)
Number of samples	1000	1000
Number of tunes	500	2000
Number of chains	4	4
Number of core	4	4
Target acceptance	0.95	0.99

#### Classification using XGBoost (Analysis 1)

To test the first hypothesis, we trained an XGBoost classifier^76^ to predict a pair’s performance in the joint-action task using several measures collected during the individual-action task: eye-movement behavior, pre-contact swing and contact, heart rate, and movement energy. We also added static participant characteristics. The classifier’s response variable was whether the pair performed above or below the level expected from their two individual scores. Because the model weighs all of these measures together, it lets us ask not only whether anticipatory gaze behavior and motor response measured during the individual-action task carry information about how well a pair coordinates in the joint-action task, but also how important they are relative to the other variables.

The model was evaluated using stratified threefold cross-validation, a strategy chosen to mitigate overfitting given the limited sample size (*n* = 64 pairs and 38 predictors). In each fold, 67% of the 64 available instances were used for training and validation, and 33% were held out for testing. Stratification preserved balanced representation of both outcome classes across folds, and the held-out partition was never accessed during model fitting, providing an unbiased estimate of out-of-sample performance. Hyperparameter tuning was performed on the training data using the Tree-structured Parzen Estimator algorithm^77^ prior to cross-validation, ensuring that no information from the test set influenced model selection. The optimized parameters were then fixed and applied during model training and the subsequent computation of SHapley Additive exPlanations (SHAP) values and confidence intervals (see Supplementary Information).

To address multicollinearity among predictors, correlation-adjusted SHAP values were computed^59^. This approach accounts for feature dependencies by combining an empirical distribution method with either a Gaussian or a Gaussian copula model. The empirical distribution preserves true marginal distributions and complex dependencies, whereas the Gaussian copula assumes linear dependencies that may introduce artifacts. A hybrid method integrating both empirical and Gaussian approximations was adopted to balance accuracy and generalizability. SHAP values were computed on the held-out test set of each fold, with the training set serving as the background distribution. Computing attributions on unseen data ensured that the reported feature importances reflected patterns that generalized beyond the training sample rather than artifacts of model fitting.

The robustness of predictions and feature importance estimates was assessed by computing confidence scores for accuracy, the Matthews correlation coefficient (MCC), and the mean absolute SHAP value. Confidence intervals were estimated through bootstrapping (*n* = 50 samples) with test-set resampling with replacement.

#### Hierarchical Bayesian *T*-test (Analysis 2)

The second hypothesis concerns whether coordination quality is visible in behavior during the interaction itself. To test it, we compared well-coordinated and less-coordinated pairs on their anticipatory gaze behavior and movement dynamics during the joint-action task, together with their static characteristics. We tested these group differences using Bayesian *t*-tests^78^, which quantify uncertainty and allow each difference to be interpreted in probabilistic terms.

Discrete outcomes, such as the number of corrective saccades in Phases 1 and 2, were modeled using a Poisson likelihood, with the mean parameter assigned a truncated-normal prior (lower bound = 0). Continuous predictors were modeled using a skewed Student’s *t*-distribution, with a normal prior placed on the location parameter. These models were fit to 9056 data points (5338 above-expected and 3718 below-expected).

For predictors with fewer observations, such as static characteristics, physiological state, and motion energy (*n* = 64 pairs), we used a standard (non-skewed) Student’s *t*-distribution instead. This choice reduced the number of hyperparameters by omitting the skewness term required in the skewed model, which improved model stability. To account for the nested structure of the data and the variability across sessions and individuals (in both hitter and receiver roles), we implemented a hierarchical model with varying intercepts at the session and individual levels (Eq. (2)). See Supplementary Information for details of the prior distributions used.

2 $$\begin{array}{rcl}&&{y}_{i}^{({{\rm{above}}})} \sim T(a,b,{\mu }_{1},{\sigma }_{1})\hfill\\ &&{y}_{i}^{({{\rm{below}}})} \sim T(a,b,{\mu }_{2},{\sigma }_{2})\hfill\\ &&{\mu }_{k} \sim N(\overline{x},1)+\alpha \hfill\\ &&\alpha ={\alpha }_{{{\rm{session}}}}+0.5* ({\alpha }_{{{\rm{subject}}}1}+{\alpha }_{{{\rm{subject}}}2})\\ \end{array}$$

We standardized all continuous predictors using Z-score before performing sampling with a U-Turn sampler^75^ and optimized hyperparameters (Table 1). The estimation convergence was evaluated using R-hat and Effective Sample Size (ESS) (see Supplementary Information).

#### Bayesian Generative Model (Analysis 3)

The third hypothesis asks what drives accurate gaze anticipation during successful coordination. To address it, we fitted a hierarchical Bayesian generative model to each participant’s Phase-1 visual-angle error in the joint-action task (*y*_*f*_). The model expressed this error in terms of the participant’s prior expectation (*P*) and the incoming sensory evidence ($\hat{X}$), allowing us to examine the relative importance of each.

The model was fit to 3330 data points (1749 above-expected and 1581 below-expected). The observation model assumed that the true posterior distribution of *y*_*f*_ followed a Skewed Student-*t* distribution, parameterized by a location parameter *μ*, a scale parameter *σ*, and skewness and kurtosis parameters *a* and *b*, respectively:

3 $${y}_{f} \sim T(a,b,\mu ,\sigma ).$$

The mean structure *μ* combined prior expectations, reflecting internal models, with sensory evidence from the joint task:

4 $$\mu =(1-w)\,P+w\,\hat{X},$$

where *P* denotes the expectations, $\hat{X}$ denotes the latent variable representing current sensory evidence, and *w* ∈ [0, 1] is a weight determining the relative reliance on prior expectations versus sensory evidence. The model included group-level variation in *w*, allowing each group to be characterized by different weighting parameters. The weight was defined as the relative variance of the sensory evidence compared to the total variance of expectations and sensory signals:

5 $${w}_{i}=\frac{{\sigma }_{{{\rm{sensed}}}[i]}^{2}}{{\sigma }_{{{\rm{sensed}}}[i]}^{2}+{\sigma }_{{{\rm{prior}}}[i]}^{2}},$$

such that lower weights reflect stronger reliance on prior expectations (proactive strategy), while higher weights indicate stronger reliance on sensory input (reactive strategy). The expectations component was parameterized as:

6 $$P={\alpha }_{{{\rm{subject}}}[i]}+{\alpha }_{{{\rm{session}}}[k]}+{\epsilon }_{{{\rm{prior}}}},$$

where *α*_subject_ captures subject-level differences in prior expectations (parameterized from the mean and variance of Phase-1 error in the individual-action task), *α*_session[*k*]_ controls for systematic shifts across sessions, and *ϵ*_prior_ denotes Gaussian noise corrupting the prior.

The sensory evidence was encoded as a latent projection:

7 $$\hat{X}={{{\bf{X}}}}^{\top }\beta +{\alpha }_{{{\rm{session}}}[k]}+{\epsilon }_{{{\rm{sense}}}},$$

where **X** comprised the relevant trial features (Phase-1 and Phase-2 visual-angle errors, Phase-3 pursuit duration, and pre-contact swing and contact predictors from the preceding event), *β* are regression weights, *α*_session[*k*]_ again accounts for session-level variation, and *ϵ*_sense_ denotes Gaussian noise corrupting sensory evidence.

This model therefore estimated participants’ prediction error in joint action as a precision-weighted combination of prior expectations and current sensory input. By comparing group-level parameters, we examined whether well-coordinated pairs relied more on proactive (prior-based) or reactive (evidence-based) strategies.

#### Handling of repeated individual observations

As described in the experimental procedures, sessions comprised either two or three participants depending on whether a cancellation occurred (see Supplementary Information). The number of available partners determined whether a given individual contributed to one or two dyads in the joint-action condition.

Analysis 1 was conducted at the dyad level. Each predictor was constructed as a combination of the two individuals’ values within a pair, and dyads were treated as the unit of analysis. Because the predictors describe pair-level properties that vary with partner identity, two dyads involving the same participant are not redundant: they correspond to different interaction conditions and produce different predictor values.

Analyses 2 and 3 were conducted at the individual level. Repeated observations from the same participant were accounted for by including by-participant and by-session random intercepts in the models. This specification absorbed individual and session baseline differences into the model and ensured that the estimated effects were not biased by the unequal number of observations per participant.

## Results

**Fig. 1:**
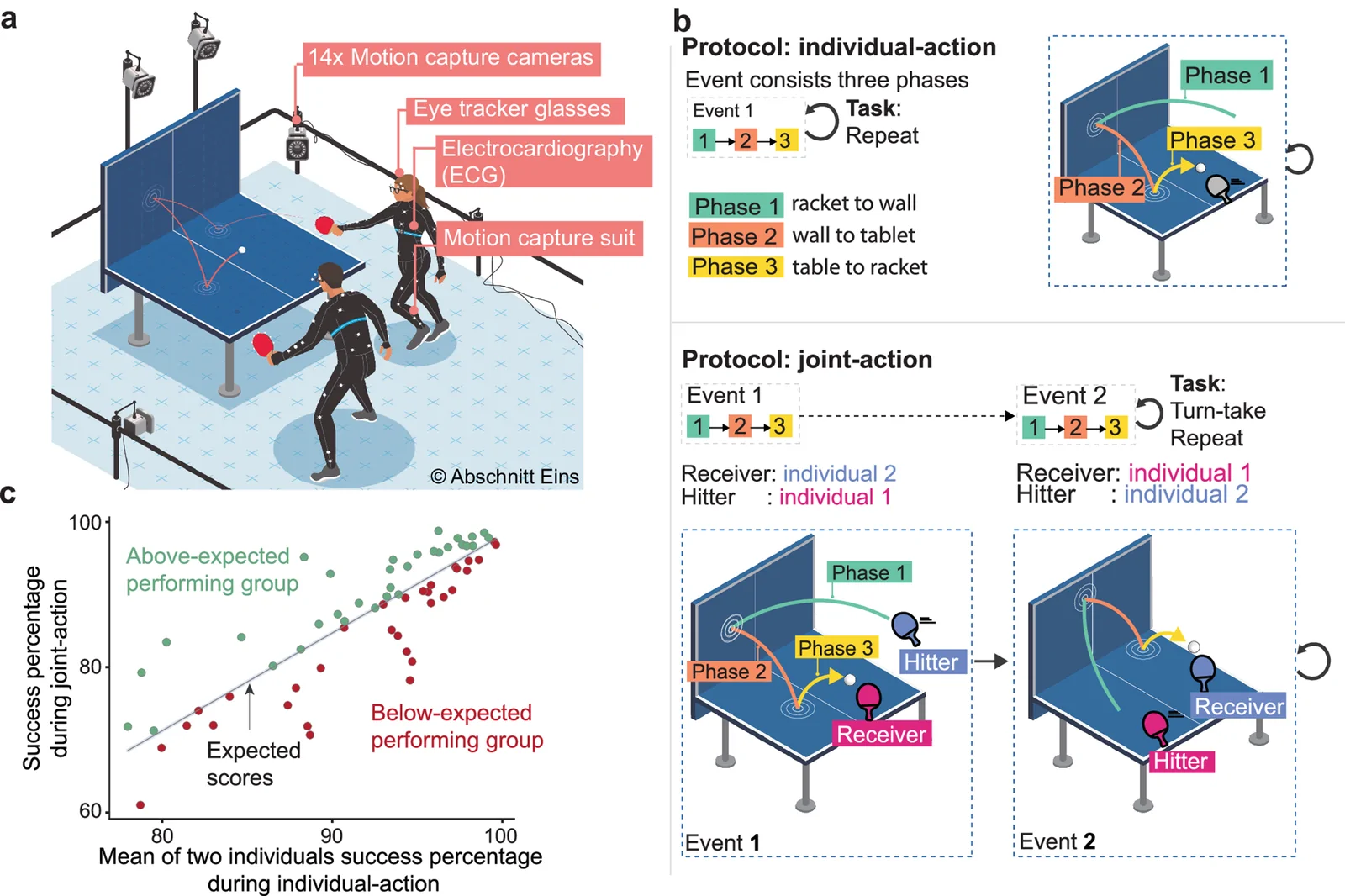
Experimental setup, protocol, and task performance for individual and joint-action tasks. **a** Experimental setup. The room was equipped with 14 motion capture cameras. Participants wore eye-tracking glasses, motion capture suits, and an ECG sensor. **b** Experimental protocol for individual and joint-action tasks. In both conditions, participants hit the ball individually (individual-action) or alternated hits with a partner (joint-action), continuing the rally for as long as possible. If they failed, they started a new rally. **c** Participants’ mean success percentages during the individual and joint-action tasks (left), and the categorization of pairs that performed “above expected” and “below expected” during joint-action tasks (right). Dots represent each pair’s mean scores, and the line represents the expected scores for each pair, fitted using a Bayesian Gaussian Linear Model based on the mean of the pair’s individual-action scores (see 2).

Our final dataset consisted of 70 participants forming 64 pairs. Participants took part in a “turn-taking ping-pong” experiment. Participants were instructed to hit the ball against a wall as many times as possible within approximately 3 min (individual-action task) or 5–6 min (joint-action task). Each participant completed both the individual-action task (hitting the ball alone) and the joint-action task (taking turns hitting the ball with a partner; Fig. 1b), with the order of tasks counterbalanced across participants. A *rally* refers to a continuous sequence of ball hits between participants until a failure occurs. An *event* begins immediately after a participant hits the ball and ends when the ball is next struck—either by the same participant (in the individual-action task), by their partner (in the joint-action task), or when a failure occurs (in both tasks).

On average, participants successfully hit the ball back in 90.82% (SD = 10.53%) of events during the individual-action task, and in 85.21% (SD = 11.33%) of events during the joint-action task. To examine joint performance relative to individual skill, we estimated the expected joint-action skill for each pair using a Bayesian GLM, based on the mean of the pair’s individual-action scores. Pairs performing above this expected value were classified as “above-expected,” and those below as “below-expected.” Because this classification is defined relative to individual skill, baseline expertise is already accounted for before any predictor is evaluated. To examine skill effects further, we also included the mean and between-partner difference in skill as predictors in Analysis 1 and compared them between groups in Analysis 2.

To examine how individuals anticipated the consequences of their partner’s and their own actions and the ball trajectory, we divided each event into three phases based on the ball’s bouncing points (Fig. 1b): (Phase 1) the ball is struck by the racket and bounces off the wall; (Phase 2) it rebounds and lands (i.e., bounces) on the table; (Phase 3) it bounces up from the table, allowing the racket to make contact again. Note that after a participant (the hitter) hits the ball, it returns to the same participant in the individual-action task, whereas in the joint-action task, it returns to their partner (the receiver).

**Fig. 2:**
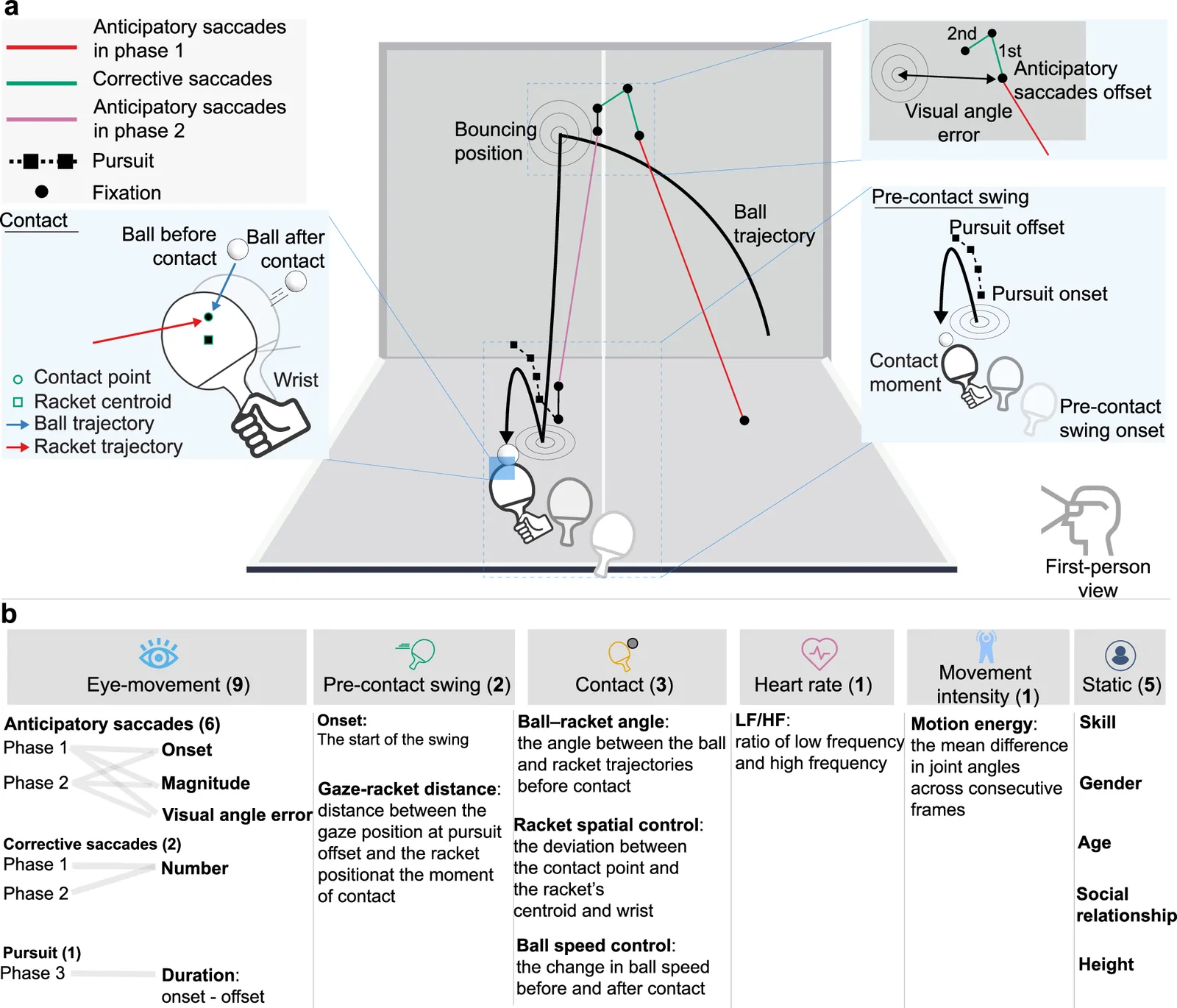
Definition of predictors extracted from eye, body, and heart-rate measures. **a** Participants’ typical eye movement and racket maneuvers from a first-person view. The red line represents anticipatory saccades in phase 1, with onset (start of the line) and offset (end of the line). Green lines indicate corrective saccades occurring after anticipatory saccades, while magenta represents anticipatory saccades in phase 2. Pursuit is shown as squares with a dotted line, and fixations as black-filled circles. **b** Predictors used in this study, categorized into six groups: eye movement, pre-contact swing, contact, heart rate, motion energy, and static characteristics. The eye movement group contains 9 predictors, pre-contact swing 2, contact 3, heart rate 1, motion energy 1, and static characteristics 5. In total, 21 predictors were extracted from three modalities: eye movement, body kinematics, and heart rate.

During Phase 1, they typically anticipated the bounce point on the wall, often making corrective saccades toward that location (Fig. 2a). In Phase 2, their gaze shifted to anticipate the bounce point on the table. During Phase 3, they closely tracked the approaching ball using either fixations or smooth pursuit eye movements.

From each participant’s data, we extracted 21 parameters, grouped into six categories, that may influence joint-action performance (Fig. 2b).

### Analysis 1: predictors of coordination success

We started by examining which parameters measured in the individual-action task best predicted coordination success in joint-action pairs. For each parameter, we derived two types of predictors: (1) the dissimilarity in individual-action predictors between the two members of a pair, and (2) the mean of their individual-action predictors. Dissimilarity was primarily quantified using the Wasserstein distance^79^, which captures differences in both mean and variance by measuring the minimal “cost” of transforming one distribution into another. For scalar features—such as static characteristics, motion energy, and heart rate—we used simple pairwise differences. These predictors were then used to train an XGBoost classifier^76^ to categorize pairs as either above-expected or below-expected in coordination performance.

Model training and evaluation followed a threefold cross-validation procedure to assess whether the model generalizes beyond the training data. Its performance was evaluated using two metrics. Accuracy was defined as the percentage of correct predictions, calculated as the sum of true positives and true negatives divided by the total number of predictions (true positives, false positives, true negatives, and false negatives). We also calculated the MCC, which ranges from −1 (indicating poor performance) to 1 (indicating perfect prediction). MCC incorporates all four components of the confusion matrix and provides a balanced measure of classification performance, even in cases of imbalanced datasets.

**Fig. 3:**
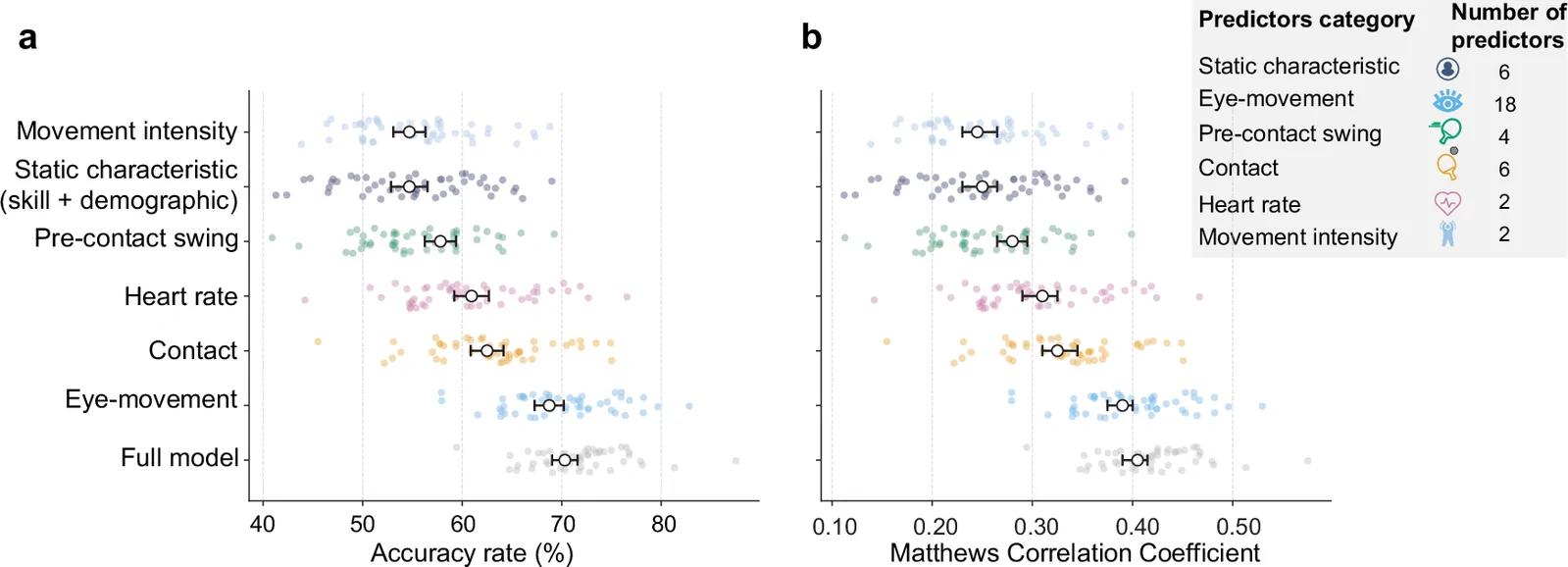
Test performance of XGBoost models trained on predictor categories from the individual-action task to classify pairs as above- or below-expected in joint-action coordination (*n* = 64 observations). Predictors included similarity and mean metrics. For the static characteristics category, the mean was computed only for skill, whereas the remaining static predictors are represented as between-partner differences. Accuracy (**a**), and the Matthews Correlation Coefficient (MCC) (**b**), were estimated on held-out test data using threefold cross-validation. Results are ordered from lowest (top) to highest (bottom). Circles indicate the scores on the test data, error bars indicate 95% bootstrap confidence intervals centered on the point estimates, and scatter points represent the distribution of scores across individual bootstrap samples. The “Full model” combines all predictor categories.

We compared the full model, which included all predictor categories, with models that each contained a single predictor category. The full model achieved the highest performance across both accuracy and MCC scores (Fig. 3). Among the single-category models, predictive performance based on accuracy and MCC scores ranked as follows: eye movement, contact behavior, heart rate, pre-contact swing, static characteristics, and movement intensity.

**Fig. 4:**
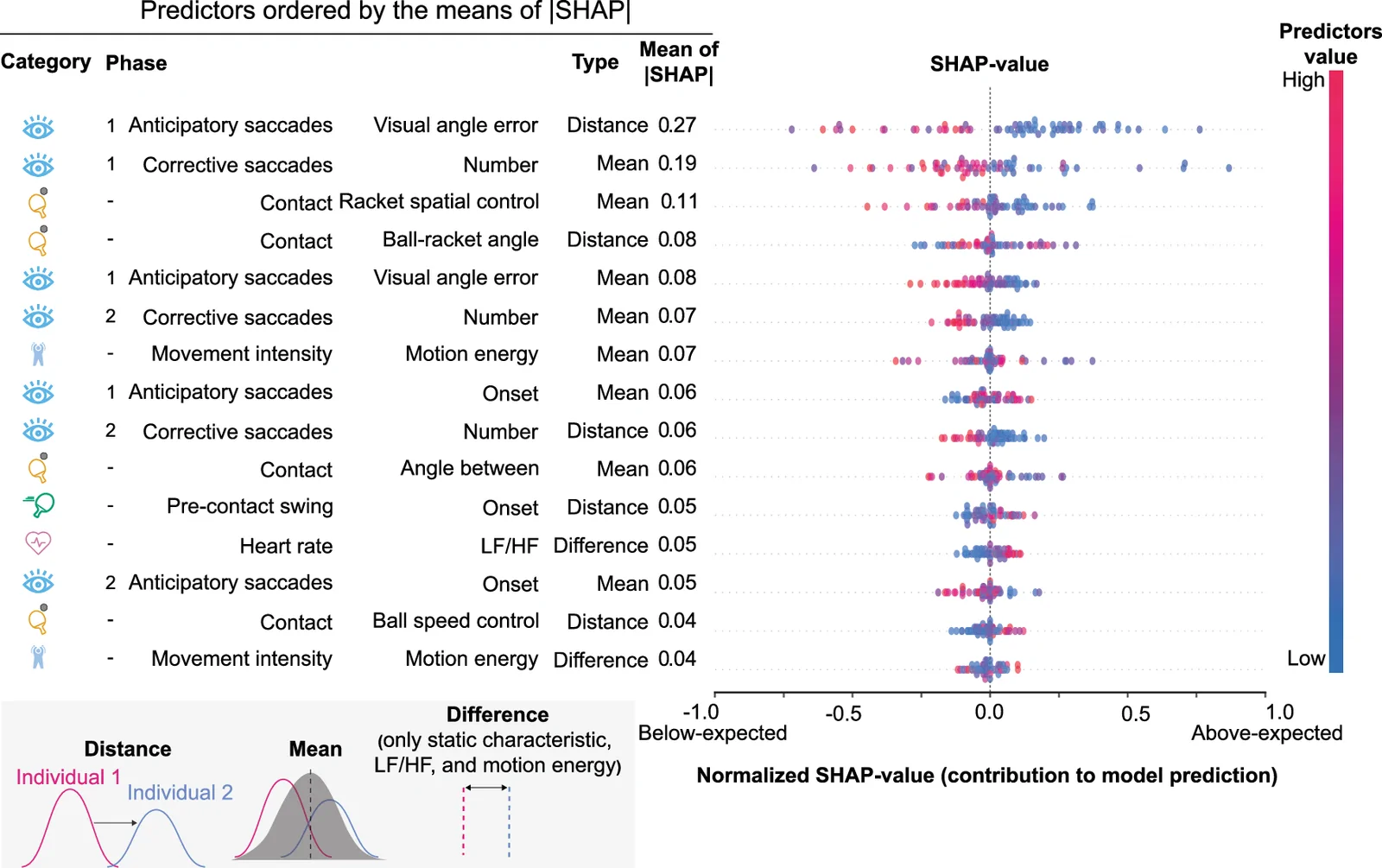
Predictors ranked by mean absolute SHAP values (∣SHAP∣), computed on the test set (*n* = 64 observations) with the training set as the background distribution. Signed SHAP values indicate the direction of each predictor’s contribution: positive values support classification into the above-expected group, negative values into the below-expected group. Predictor values are color-coded, with high values in red and low values in blue.

To identify the most informative predictors, we computed feature importance using correlation-adjusted SHAP values^59^. We computed SHAP values using the full model, and Fig. 4 shows the top 15 predictors, ranked by their mean absolute SHAP values.

Among the top 15 predictors, 7 were related to eye movement, 4 to contact behavior, 2 to movement intensity, 1 to pre-contact swing, and 1 to heart rate. No predictors from the static characteristics (including skill difference and mean) category appeared in the top 15. Notably, two predictors stood out for their strong influence: the dissimilarity (Wasserstein distance^79^) in visual angle error and the mean number of corrective saccades during Phase 1. Their SHAP values were approximately three and two times greater, respectively, than the average SHAP value of the top 15 predictors (mean = 0.08). Based on these findings, we constructed a parsimonious model using only these two top predictors. Remarkably, this simplified model achieved an accuracy of 75% (95% CI = [73.66%, 76.34%]), slightly outperforming the full model (+4.69%).

### Analysis 2: behaviors distinguishing well- and less-coordinated pairs

To characterize the behavioral features that distinguish above-expected from below-expected groups, we compared predictors of joint-action performance between the two groups using Bayesian *t*-tests^78^.

We divided the individuals within each pair into hitter and receiver, and computed eye-movement predictors separately for each role. From the hitter’s perspective, these predictors reflect how participants anticipate the outcome of their own actions after striking the ball (Phase 1), as well as how they monitor the receiver’s behavior after the ball bounces off the wall (Phases 2 and 3). From the receiver’s perspective, the predictors reflect how participants anticipate the hitter’s actions during Phase 1 and prepare their responses during Phases 2 and 3. For pre-contact swing and contact behavior, predictors were extracted only from the receiver, because the receiver in the current event is the participant whose pre-contact swing and contact were being executed at that moment.

In addition, to assess positional alignment between the two individuals in a pair, we introduced two new predictors under this category. Pair-centered deviation was defined as the Euclidean distance between the midpoint of the hitter’s and receiver’s positions and the ball’s bounce point in Phase 1. Post-hit table proximity retreat captured how much the hitter moved away from the table after making their shot and before their partner returned the ball; lower values indicated that the hitter retreated, creating more space for the receiver to return the ball. These measures capture spatial accommodations that partners make for one another, which previous research suggests facilitate interpersonal coordination by enabling rapid mutual adjustment^80^.

**Fig. 5:**
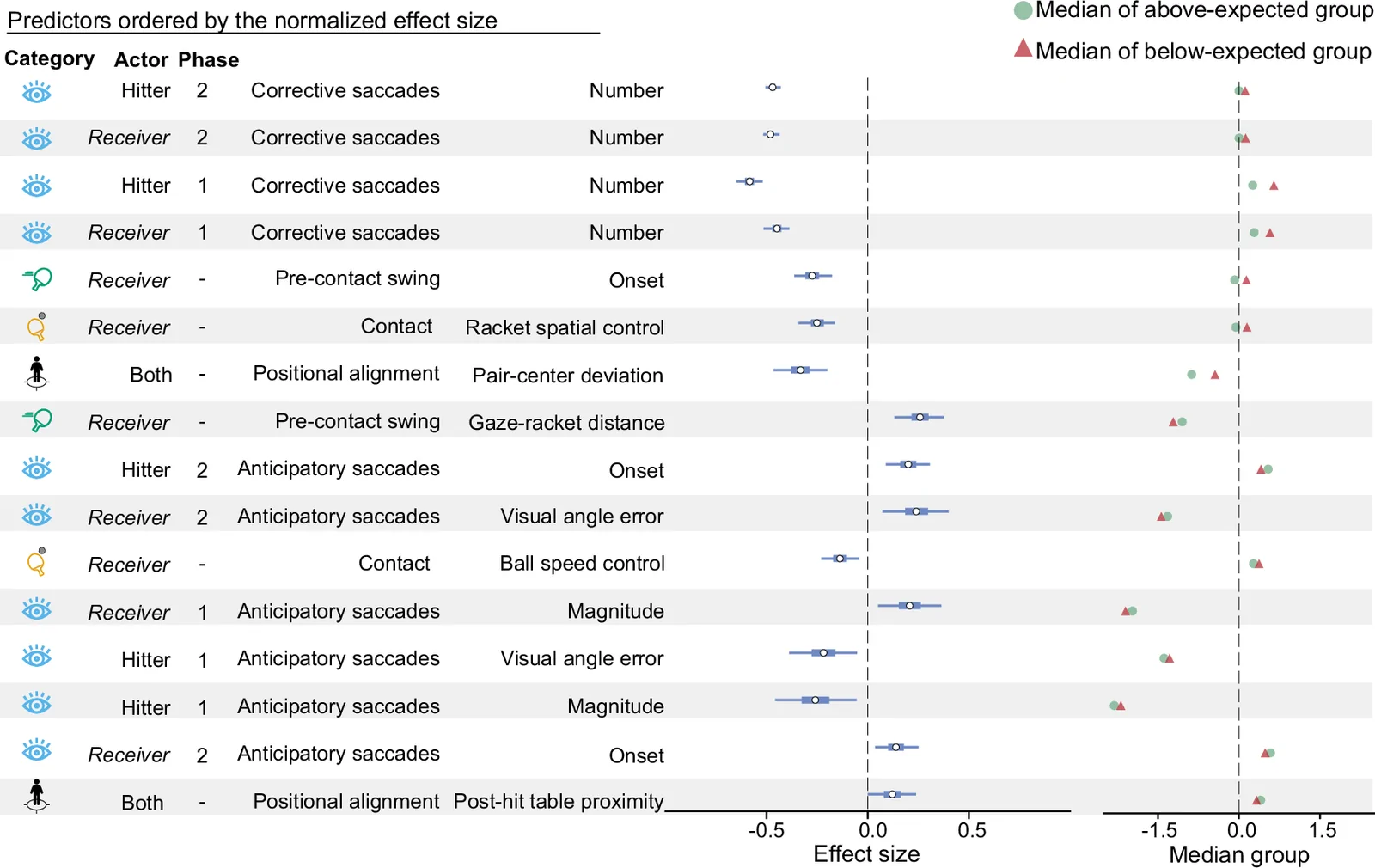
Median predictor values for the above- and below-expected groups (right), and Bayesian effect sizes (left), defined as the posterior mean differences between groups expressed in standard deviation units. Error bars and boxes in the effect size plots represent 95% highest density intervals (HDIs) and the interquartile ranges of the posterior distributions, respectively. For static, heart rate, and motion energy predictors, one value was recorded per pair (*n* = 64 observations). For eye movement, pre-contact swing, contact, and positional alignment predictors, values reflect the total number of events across pairs (*n* = 9056 observations).

Figure 5a presents only the predictors that showed credible differences in the Bayesian *t*-test, ordered by the normalized effect size. Credibility was defined as 95% highest density intervals (HDIs) that do not include zero, reflecting high certainty in the presence of an effect.

The above-expected and below-expected groups differed credibly in eye movement, pre-contact swing, contact behavior, and positional alignment. They showed no credible differences in static characteristics (including skill difference and mean), heart rate, movement intensity, or self-assessment (see Supplementary Information). The most pronounced effects appeared in corrective saccades: both hitters and receivers in the above-expected group made fewer of them during Phase 1, while the ball traveled toward the wall (hitters: Median effect size = −0.58, 95% HDI [−0.65, −0.52]; receivers: Median = −0.45, 95% HDI [−0.51, −0.39]), and during Phase 2, while the ball traveled toward the receiver (hitters: Median =  −0.47, 95% HDI [−0.51, −0.43]; receivers: Median = −0.48, 95% HDI [−0.52, −0.44]).

Hitters and receivers in the above-expected group also differed from the below-expected group in their anticipatory looking, though in subtler ways. Hitters made smaller (Median = −0.26, 95% HDI [−0.46, −0.05]) and more precise (Median =  −0.22, 95% HDI [−0.39, −0.05]) anticipatory saccades during Phase 1, and they initiated these saccades later in Phase 2 (Median = 0.20, 95% HDI [0.09, 0.31]), just before the ball bounced and pursuit began. Receivers, by contrast, made larger anticipatory saccades in Phase 1 (Median = 0.21, 95% HDI [0.05, 0.36]), again alongside fewer corrective saccades (Median = −0.45, 95% HDI = [−0.51, −0.39]). In Phase 2 they too initiated saccades later (Median = 0.14, 95% HDI [0.04, 0.25]) and with lower precision (Median = 0.24, 95% HDI [0.07, 0.40]), once more with fewer corrective saccades (Median = −0.48, 95% HDI = [−0.52, −0.44]), which may point to reduced reliance on visual feedback.

Additionally, receivers in the above-expected group were better at controlling the ball and more prepared to return it. They began their pre-contact swings earlier (Median = −0.27, 95% HDI [−0.36, −0.18]) and kept a larger gaze-to-racket distance, that is, a larger gap between gaze position at pursuit offset and the racket at contact (Median = 0.26, 95% HDI [0.13, 0.38]). They controlled the ball’s position more tightly, with smaller deviations between the wrist-to-racket centroid and the contact point (Median = −0.25, 95% HDI [−0.34, −0.16]), and its speed more tightly, with smaller changes in ball speed from before to after contact (Median = −0.14, 95% HDI [−0.23, −0.04]).

Finally, participants in the above-expected group showed better positional alignment. They produced smaller pair-centered deviations, hitting the ball closer to the midpoint between themselves and their partner (Median = −0.33, 95% HDI [−0.46, −0.20]), and they remained closer to the table after hitting, retreating less than participants in the below-expected group before their partner returned the ball (Median = 0.12, 95% HDI [0.00, 0.24]).

### Analysis 3: internal models and prediction during coordination

To directly test whether participants’ predictions were driven primarily by prior expectations, reflecting internal models (proactive strategy), or by sensory information available immediately before the hit (reactive strategy), we applied a Bayesian generative model. This model estimated participants’ anticipatory accuracy during Phase 1 of the joint-action task, measured as visual-angle errors.

Formally, predictions were modeled as a weighted mixture:

$$\mu =(1-w)P+w\hat{X},$$

where *P* represents prior expectations and $\hat{X}$ represents latent sensory evidence (see Section “Methods” for details). Lower *w* values indicated stronger reliance on prior expectations (proactive strategy), whereas higher *w* values indicated stronger reliance on sensory evidence (reactive strategy).

**Fig. 6:**
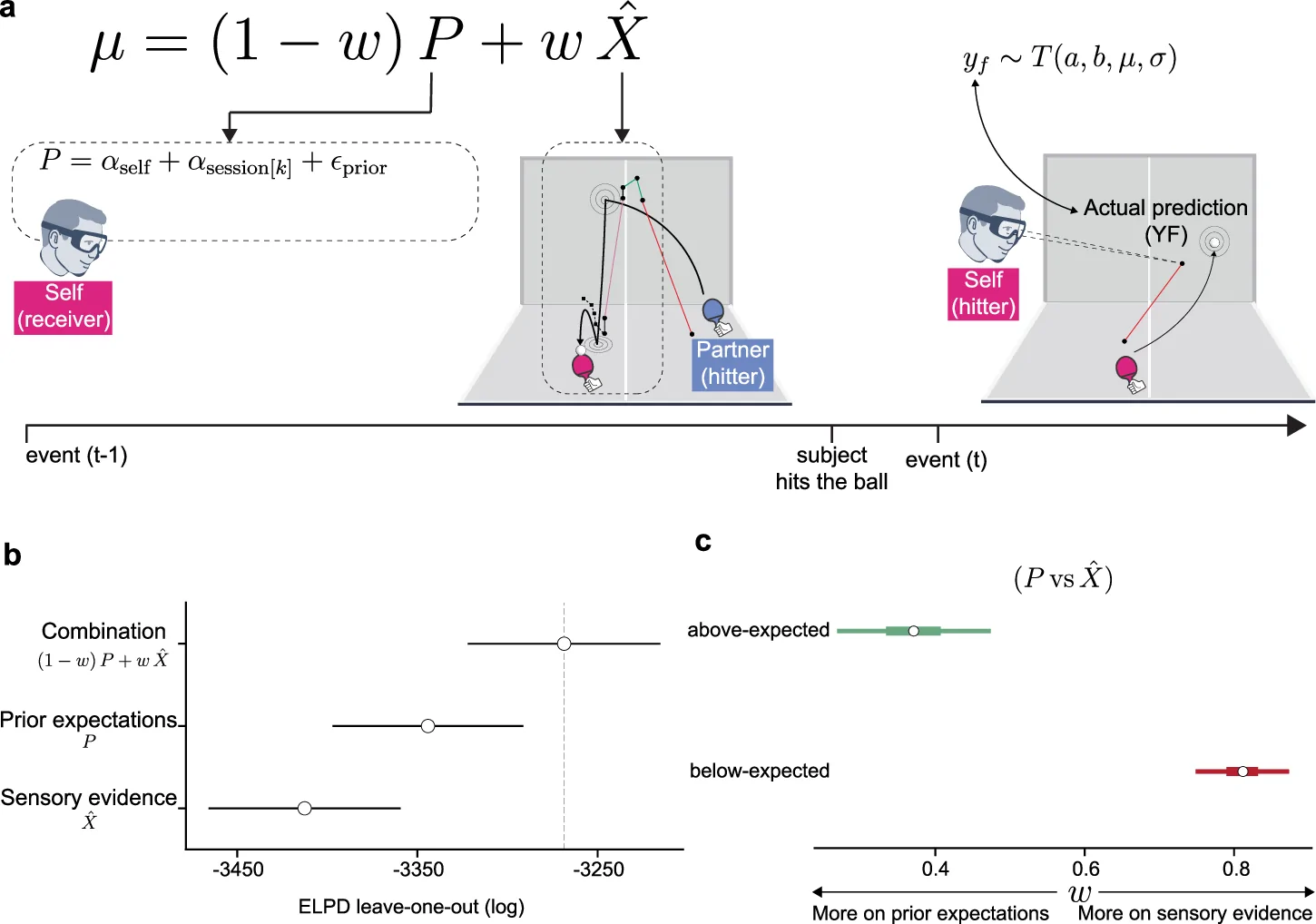
Model comparison and sensory-evidence weighting across above- and below-expected groups. **a** Conceptual illustration of the Bayesian generative model examining how individuals integrate prior expectations (*P*) and sensory evidence ($\hat{X}$) during the joint-action task. **b** Model comparison (*n* = 3330 observations) of exclusive reliance on *P*, exclusive reliance on $\hat{X}$, and reliance on their combination ($P+\hat{X}$), evaluated using expected log pointwise predictive density (ELPD). Circles and error bars indicate means and standard deviations of ELPD scores. **c** Estimated sensory-evidence weights for participants in the above-expected and below-expected groups. Circles denote posterior medians, error bars represent 95% highest-density intervals (HDIs), and boxes indicate interquartile ranges of the posterior distributions.

We compared three models to test whether participants relied exclusively on prior expectations (*P*), exclusively on sensory evidence ($\hat{X}$), or on a combination of both. The model comparison (Fig. 6b) indicated that participants relied on both sources simultaneously, but placed greater weight on prior expectations (*P*) than on sensory evidence ($\hat{X}$), as reflected in the ELPD scores. Moreover, participants in the above-expected group assigned less weight (Median posterior of *w* = 0.37, HDI 95% = [0.27, 0.47]) to sensory evidence than those in the below-expected group (Median = 0.81, HDI 95% = [0.75, 0.87]), indicating a stronger reliance on prior expectations (Fig. 6c).

## Discussion

Although most studies of coordination rely on simplified laboratory tasks and infer predictive processing indirectly from reaction times^25,29,30^, we examined coordination in a dynamic turn-taking ball-hitting task requiring continuous full-body interaction and measured prediction through anticipatory eye movements. We tested whether anticipatory gaze behavior and motor performance predict successful coordination. Using multimodal behavioral and physiological measurements together with machine learning and Bayesian modeling, we found that successful coordination depended strongly on aligned predictive control between partners, particularly in how individuals predicted the consequences of their own actions. Partners whose self-predictions were more closely aligned coordinated more successfully, suggesting that effective coordination emerged when individuals generated compatible expectations about the outcomes of their own actions.

This finding is broadly consistent with previous work linking predictive gaze behavior to internal models in motor control. During object manipulation and ball sports, gaze typically precedes future contact locations, allowing individuals to prepare upcoming actions efficiently^52,53,55,57,58,81^. Our results extend these findings from individual sensorimotor control to joint action by showing that similarity in anticipatory control across individuals predicts coordination success, even after controlling for individual skills. The importance of aligned predictive styles is also consistent with findings from musical duet performance^12^, where coordination is enhanced when anticipated action consequences match an individual’s own sensorimotor expectations. Similarly, dyads in our task achieved higher levels of coordination when their predictions about the consequences of their own actions were more closely aligned.

Our findings highlight an additional distinction that is often difficult to examine in coordination research: the difference between predicting the consequences of one’s own actions and predicting those of a partner. Most coordination paradigms assess performance through joint outcomes (e.g., refs. ^49,51^), making these two forms of prediction difficult to disentangle empirically. Because our task alternated self-generated and partner-generated ball trajectories, we were able to examine anticipatory gaze behavior separately for each.

As hypothesized, well-coordinated and less-coordinated pairs differed in both anticipatory gaze behavior and movement dynamics during interaction. Well-coordinated pairs showed greater anticipatory control during the joint-action tasks. Hitters in well-coordinated pairs predicted the bounce locations of the balls they hit more precisely and made fewer corrective saccades. Receivers in well-coordinated pairs, by contrast, often produced coarser predictions of their partner’s actions, yet still required fewer corrective saccades. Together, these findings suggest that coordination success depended less on detailed prediction of a partner’s actions and more on producing self-generated actions with predictable outcomes.

Our Bayesian generative model quantified the relative contributions of prior expectations and incoming sensory evidence to prediction error during the joint-action task. Well-coordinated pairs assigned greater weight to prior expectations derived from their own sensorimotor experience and less weight to real-time sensory evidence when predicting future ball locations after their own hits. This pattern suggests that anticipatory control relied primarily on internal models that generated expectations about future action outcomes, rather than on online prediction based solely on currently available sensory information. This interpretation is broadly consistent with predictive-processing accounts of joint action, which propose that successful interaction depends on internal generative models that anticipate future states and minimize prediction error^26–28^. Importantly, our findings extend this framework by showing that individual differences in the balance between prior-based and sensory-based prediction are associated with coordination success. Successful coordination was linked specifically to stronger reliance on internal-model-based prediction of the consequences of one’s own actions.

Our results indicated that, while prediction appeared to play an important role, demographic, physical, physiological, and relational variables contributed relatively little to coordination success in our task. This pattern contrasts with findings from other joint-action paradigms in which body morphology constrains physically demanding tasks such as joint lifting^10^, interpersonal closeness modulates shared action representations in joint Simon tasks^30^, or physiological synchrony contributes to conversational and cooperative interactions unfolding over longer timescales^36,67^. One possible explanation is that the relative importance of coordination mechanisms depends on the temporal and sensorimotor demands of the task. In rapidly unfolding interactions such as the present ball-exchange task, where participants must continuously anticipate future action outcomes on a sub-second timescale, coordination may rely more heavily on anticipatory control. In contrast, slower or more socially mediated interactions might allow greater contributions from interpersonal, physiological, or relational alignment processes.

### Limitations

Several limitations should be acknowledged. Although the ball-exchange task captures more naturalistic dynamics than many laboratory paradigms, it remains a constrained coordination task characterized by turn-taking and relatively short interaction periods. As a result, the mechanisms identified here may not generalize fully to other forms of coordination, such as long-term cooperation, communication-rich interactions, physically interdependent tasks, or larger groups. Furthermore, although our design partly dissociated prediction of one’s own actions from prediction of a partner’s actions, these processes are likely to operate in a more integrated manner during everyday social interactions.

A further potential limitation concerns statistical inference. Some participants contributed to more than one dyad, creating a degree of dependence among observations, although this was accounted for statistically where possible. In addition, while the sample was sufficient to detect the strong contribution of prediction to coordination success, it may have been less sensitive to smaller effects associated with demographic, physical, physiological, and relational variables. Consequently, the limited contributions of these factors should be interpreted cautiously until replicated in larger samples.

## Conclusion

A central challenge in understanding social interaction is identifying the mechanisms that enable successful coordination in dynamic, real-world settings. By combining multimodal behavioral measurements with machine learning and Bayesian modeling, we quantified the relative contributions of predictive, physiological, motor, demographic, and relational factors to coordination success during joint action. Across analyzes, predictive measures consistently emerged as the strongest determinants of performance. These findings suggest that successful coordination depends less on who the interacting partners are and more on how similarly they anticipate the consequences of their actions. More broadly, our framework provides a quantitative approach for disentangling the multiple mechanisms that contribute to naturalistic social coordination. One promising next step is to apply this framework to more complex collaborative domains, including physical human-robot interaction, to test whether successful coordination likewise depends on aligned predictive control across agents^82^.

## Supplementary information

Transparent Peer Review file

Supplementary Information for Successful coordination emerges from aligned self-predictions in a dynamic motor interaction

Description of Additional Supplementary Files

Movie S1

Movie S2

## Data Availability

Deidentified participant data are publicly available under a CC BY 4.0 license here: for publication.

## Code availability

Analysis code is publicly available under a CC BY 4.0 license here: for publication.
