# Supplementary material for: Successful coordination emerges from aligned self-predictions in a dynamic motor interaction
Source: Commun Psychol. 2026 Aug 20;4:119. doi: 10.1038/s44271-026-00523-7 (PMC13493972; doi:10.1038/s44271-026-00523-7)
Supplement: Supplementary file 1 [file 44271_2026_523_MOESM1_ESM.pdf]

nature portfolio

Peer Review File

Successful coordination emerges from aligned self-predictions in a dynamic motor interaction

Corresponding Author: Dr Prasetia Utama Putra

This file contains all reviewer reports in order by version, followed by all author rebuttals in order by version.

Version 0:

Reviewer comments:

Reviewer #1

(Remarks to the Author)
This study is an ambitious attempt to quantify "what makes a good team" using a naturalistic task with many converging measurements. It is a clear and well written report of a soundly executed study. That being said, I do see some outstanding issues with the analysis that I would need clarification on before I recommended this paper for publication:

1) You have a lot of predictors (even 18 for eye movement) which makes me wonder whether there is an underlying correlational structure or some latent variables. Did you attempt any dimension reduction (e.g., PCA) to try and reduce the space to less predictors. I know tree-based methods are relatively robust to multicollinearity, but I wonder whether you could be fully confident about this? I'm asking this because probably you only have twice the amount of dyads as you have predictors (which isn't a great ratio for models like xGBoost) so I would worry about overfitting. I would suggest, #1 doing PCA and extracting the latent structure of your data (there might be some things that are highly correlated so you can collapse); #2 report variance inflation diagnostics

2) You have such rich data - it seems like a missed opportunity to reduce them to a summary of the interaction (e.g., one number per parameter per phase). Have you thought about binning your data into segments and looking at how these parameters vary over time. For instance you could calculate each parameter in 30 second windows (or however many you think is useful) to see how the parameters unfold over time. For me, this is not mandatory, but would make the paper stronger. In this case, you would definitely need to do some dimension reduction.

Reviewer #4

(Remarks to the Author)
The study addresses key research questions regarding joint actions and analyses the extent to which various dyadic factors influence performance in a cooperative table tennis task. To this end, three key research questions have been formulated: (1) What characterises successful cooperation? Specifically, the study examines the extent to which anticipatory eye movements and behaviours, in combination with demographic, physical and interpersonal factors, predict better coordination during joint actions. (2) What factors distinguish well-coordinated pairs from less well-coordinated pairs? (3) In the context of joint actions, are predictions in well-coordinated pairs based more on internal models (expectation-based) or on real-time sensory information (sensory-based)? A multi-method approach was adopted to investigate these research questions. A laboratory study utilised 3D motion tracking, eye tracking, heart rate measurements and standardised questionnaires. The participants’ task was to play a game of table tennis against a wall, with both a single action and a joint action condition being implemented. Various inferential statistical methods were used to analyse the data, including hierarchical Bayesian t-tests and Bayesian generative models. Overall, the results show that successful coordination in joint actions relies less on continuous, detailed observation of one’s partner and more on stable, predictable and proactively controlled individual actions. Well-coordinated pairs were characterised by stronger and more consistent anticipatory control, basing their predictions primarily on internal models (expectation-based) rather than on real-time sensory information. This combination of anticipatory control and reliable self-stability appears to form an efficient basis for successful joint actions.
The study makes an original and relevant contribution to the field of research and is distinguished by its use of modern data analysis methods. The methodological approach can be regarded as solid overall, although some aspects remain

unresolved and certain theoretical constructs are not fully clarified in the argumentation.
Major commands:
1. Overall, the theoretical framework of the study has not yet been sufficiently developed. In particular, there is no systematic integration of the findings from relevant primary studies, which means that the theoretical derivation remains somewhat unclear in places. Greater reference to empirical literature would also enable a clearer derivation of specific hypotheses, which are currently entirely absent.
Furthermore, a more precise theoretical foundation would help to better distinguish between the two central approaches – internal models as an expectation-based prediction strategy and sensory-based real-time analysis – and to situate them conceptually. It remains unclear in which studies these mechanisms have already been empirically demonstrated, which findings are consistent, and where open research questions still exist.
Furthermore, a clearer distinction would be desirable as to whether and to what extent the use of these predictive approaches in joint actions differs systematically depending on the task at hand. Overall, a more in-depth examination of the primary literature would significantly sharpen the theoretical argumentation and considerably strengthen the derivation of the research questions and hypotheses.
2. Overall, the discussion is well-structured and presented in a clear and logical manner. At the same time, there are some inconsistencies in the content in certain places, which stem from the theoretical framework.
In particular, the findings are sometimes only partially contextualised in relation to existing primary literature, which means that the classification of the results is not consistently refined. A stronger link to established empirical work could have helped to interpret the results in a more nuanced way and to situate them more effectively within the research field.
Overall, the discussion remains coherent in its argumentation, but would have gained significantly in analytical depth in some places had the literature been integrated more closely.
3. Another key point concerns the composition of the dataset. It remains unclear to what extent individual participants took part in the joint-action condition once or twice, and how these repeated measurements were incorporated into the data structure. This lack of transparency represents a significant limitation, particularly with regard to data analysis.
This aspect is not addressed in the discussion or in the section on limitations, even though it means that some individuals may be represented multiple times in the dataset, whilst others contributed to only a single trial run. Particularly in the context of machine learning-based or more complex statistical methods, this represents a potential source of bias that may compromise the independence of the data points.
It would therefore have been desirable to explicitly reflect on this circumstance and discuss the extent to which it might limit the robustness of the results or influence the generalisability of the findings.

Minor commands:
1. The information in lines 112–114 is extremely important for understanding the variables under investigation. It would be worth emphasising this point here to help the reader better understand the results that follow.
2. 120-121: Clearly formulated hypotheses would be desirable. What do the authors expect to find?
3. 137: ECG Please also include common abbreviations.
4. Fig 1: The illustration is very good and largely self-explanatory. However, the font size is too small, which makes it difficult to read. The caption for the figure ends with “Abschnitt Eins.”. That appears to be a mistake.
5. 164-165 (“consistent with findings from previous eye movement research"): That belongs in the discussion section, not the results section.
6. Fig 2: I consider the list of parameters (b) to be, in principle, very good and useful. However, they are so brief that some of them (e.g. “the deviation between the contact point and the racket’s centroid and wrist”) are difficult to understand. It is very important for understanding the results section to understand the parameters examined (how exactly they were determined and what they represent). The font is too small here as well.
7. 186 (“LF/HF”): Please also include common abbreviations.
8. 218-220 (“Among the individual models, predictive performance ranked in the following order: eye movement, contact behavior, heart rate, pre-contact swing, static characteristics, and movement intensity.”): Is that the result of an analysis, or is it a ranking based on the literature?
9. Fig 3: I’m not entirely sure what the illustrations show. Could you describe them in a bit more detail? Please note the size (text and diagram) here as well.
10. 236: Is that really the right headline? Wouldn't ‘How do well-coordinated pairs differ from less coordinated pairs?’ be better?
11. 391-395: Overall, the limitations discussed do not appear to be sufficiently comprehensive and could be explored in much greater depth.
Recommendation: Major Revision
The manuscript addresses an interesting and methodologically strong study on dyadic coordination in joint action and makes use of advanced multimodal data and analytical approaches.
However, substantial revisions are needed, particularly regarding the theoretical framework, which lacks sufficient integration of the relevant literature and clear conceptual grounding, as well as the absence of explicit hypotheses. In addition, issues concerning data structure transparency and potential non-independence of observations need to be addressed. The discussion would also benefit from a deeper engagement with existing empirical work and a more comprehensive treatment of limitations.
Overall, the study is promising, but requires major revision before it can be considered for publication.

Reviewer #5

(Remarks to the Author)
The researcher explored the following two central questions using a dynamic, turn-taking ball-hitting task combined with

multimodal behavioral and physiological measurements: What factors influence successful coordination? And what distinguishes teams that coordinate effectively from those that struggle? Personally, I find this to be a very timely and interesting study. The research questions are meaningful, the methods are sound, and the writing is generally good. I only have a few minor suggestions for the authors to consider:

Change the title. There is a considerable gap between the research content and the current title. The study focuses primarily on coordination in motor tasks, whereas the current title covers a much broader scope.

In the introduction, could the authors derive specific hypotheses based on existing theories or previous studies? If not, please explain why specific hypotheses were not proposed.

Sample size. The authors have already provided a justification for the sample size. However, given that this study involves relatively complex and multiple statistical analysis methods, I suggest briefly discussing this in the limitations section.

Data structure. I noticed that sometimes three participants are tested simultaneously, and then paired in different combinations for the coordination task. Does this involve a situation where one participant takes part in two coordination tasks? Or do all participants complete two coordination tasks? My question is whether this differs from a two-person simultaneous experiment, and whether this could affect the data analysis results.

Expand the discussion. The authors could briefly discuss the mechanisms underlying coordination activities that differ from table tennis to help stimulate readers' further thinking.

Version 1:

Reviewer comments:

Reviewer #1

(Remarks to the Author)
Thank you for this. You have clarified all of my concerns — great work!

Reviewer #4

(Remarks to the Author)
The revised manuscript has improved substantially compared to the previous version. In particular, the theoretical framing has been strengthened, and several of the concerns raised in the initial review have been addressed. Nevertheless, given the complexity of the study design and the analyses performed, some aspects would benefit from further clarification before publication.

Major comments

Hypotheses and operationalisation (Lines 135–145)
It is very helpful that explicit hypotheses have now been added at the end of the Introduction. Given the complexity of the analyses, I would additionally recommend linking each hypothesis more directly to the variables used in the analyses. Specifically, it would be helpful to clarify how the conceptual hypotheses are translated into the empirical models (i.e., which independent and dependent variables are expected to be related, and in what direction). This would make the analytical approach easier for readers to follow.

Sample structure and repeated participation (Lines 187–191)
The revised manuscript now provides a clearer explanation that some participants contributed data in more than one dyad, which is a welcome addition. However, it would be helpful to report the exact numbers of participants who were included in one dyad versus those who participated in two dyads. This would further improve transparency regarding the dataset structure.

Table tennis expertise
In the Results section, it remains unclear whether differences associated with table tennis expertise were examined. Given the nature of the task, it would be useful to clarify whether expertise was analysed and, if so, whether it influenced any of the reported outcomes.

Discussion and hypotheses
I recommend returning more explicitly to the hypotheses at the beginning of the Discussion. The interpretation of the findings would benefit from a clearer structure that systematically addresses whether and how the individual hypotheses were supported by the results.

Minor comments

Line 103: The parenthesis in “(e.g., …” is not closed.
Line 157: Please also report the age range of the participants.

Reviewer #5

(Remarks to the Author)
The authors have adequately addressed my comments and concerns. I appreciate their careful revisions and wish them the best of luck with the manuscript.

Open Access This Peer Review File is licensed under a Creative Commons Attribution 4.0 International License, which permits use, sharing, adaptation, distribution and reproduction in any medium or format, as long as you give appropriate credit to the original author(s) and the source, provide a link to the Creative Commons license, and indicate if changes were made.
In cases where reviewers are anonymous, credit should be given to 'Anonymous Referee' and the source.
The images or other third party material in this Peer Review File are included in the article's Creative Commons license, unless indicated otherwise in a credit line to the material. If material is not included in the article's Creative Commons license and your intended use is not permitted by statutory regulation or exceeds the permitted use, you will need to obtain permission directly from the copyright holder.
To view a copy of this license, visit https://creativecommons.org/licenses/by/4.0/

Response to Reviewers

Dear Editor,

We sincerely thank you and the reviewers for taking the time to review our manuscript and for providing valuable and insightful comments. Your feedback has been instrumental in improving the quality of the current version. We have carefully considered each point and have made our best efforts to address all comments. We hope that the revised manuscript meets your expectations. We also welcome any further constructive feedback you may have.

Below, we provide a point-by-point response to the comments. All modifications in the manuscript have been highlighted in yellow.

Response to Reviewer 1

General comment. This study is an ambitious attempt to quantify "what makes a good team" using a naturalistic task with many converging measurements. It is a clear and well-written report of a soundly executed study. That being said, I do see some outstanding issues with the analysis that I would need clarification on before I recommended this paper for publication.

Comment 1.1

You have a lot of predictors (even 18 for eye movement) which makes me wonder whether there is an underlying correlational structure or some latent variables. Did you attempt any dimension reduction (e.g., PCA) to try and reduce the space to fewer predictors? I know tree-based methods are relatively robust to multicollinearity, but I wonder whether you could be fully confident about this. I am asking because you probably only have twice the amount of dyads as you have predictors, which is not a great ratio for models like XGBoost, so I would worry about overfitting. I would suggest: (1) doing PCA and extracting the latent structure of your data (there might be some things that are highly correlated so you can collapse); (2) reporting variance inflation diagnostics.

Response 1.1

We thank the reviewer for raising this concern about overfitting and multicollinearity. To address it, we report results from three complementary checks. The first was already part of our original analysis pipeline. The second and third were added in direct response to this comment and are now included in the Supplementary Information under the section "Variance inflation and PCA analysis".

(1) **Out-of-sample evaluation (original analysis).** Our dataset includes 64 dyads and 38 predictors. We used three-fold cross-validation, holding out 33% of the dyads in each fold

for evaluation. On these held-out samples, the model achieved an MCC of 0.41 (95% CI [0.38, 0.43]). This indicates that the model generalizes to data it did not see during training, rather than fitting noise specific to the training set. In addition, when computing SHAP values, we used an algorithm that accounts for dependencies between predictors (Aas et al., 2021).

(2) **Variance inflation diagnostics (added to Supplementary Information).** We computed variance inflation factors for the 38 predictors. More than half showed substantial inflation, confirming the reviewer's intuition that there is meaningful correlational structure in the feature space.

(3) **PCA-reduced model comparison (added to Supplementary Information).** Given the inflation observed above, we tested whether collapsing the predictor space with PCA would improve generalization. We extracted principal components from the 38 predictors and trained XGBoost separately on 2, 5, 10, and 15 components, using the same three-fold cross-validation scheme. The best PCA-reduced model reached MCC = 0.17, which is 0.24 lower than the model trained on the original predictors. If multicollinearity were the source of overfitting, the reduced models should have generalized better than the full model. We observed the opposite pattern.

Together, these three results indicate that the model effectively handles the multicollinearity present among the predictors, and that retaining the full feature set yields better out-of-sample performance than applying dimension reduction in this dataset. Furthermore, parsimonious models that relied solely on important eye-movement predictors achieved a classification accuracy of 75%, suggesting that individual eye-movement measures carry meaningful predictive information for coordination success in a joint action task.

Comment 1.2

You have such rich data; it seems like a missed opportunity to reduce them to a summary of the interaction (e.g., one number per parameter per phase). Have you thought about binning your data into segments and looking at how these parameters vary over time? For instance, you could calculate each parameter in 30-second windows (or however many you think is useful) to see how the parameters unfold over time. For me, this is not mandatory, but would make the paper stronger. In this case, you would definitely need to do some dimension reduction.

Response 1.2

We are glad to point out that an analysis of this kind is already included in the Supplementary Information under the section "Change of behavior". We summarize the approach and findings below, and we clarify why these results are in the Supplement rather than the main text.

We segmented each interaction into time windows ($n = 10$) and recomputed the predictors within each window. This allowed us to track how each parameter evolved across the course of the joint action, rather than collapsing the entire interaction into a single summary value per dyad.

The results showed two distinct temporal patterns:

- **Stable group-level differences from the outset.** Several predictors distinguished the above-expected and below-expected groups from the very first window, and these differences remained consistent across the full interaction. This pattern was observed for the number of corrective saccades during Phase 1, for both hitters and receivers, and for the timing of the pre-contact swing.

- **Gradual divergence over time.** Other predictors showed comparable values between the two groups at the start of the interaction, but diverged progressively as the task unfolded. Pair-centered deviation and post-hit table proximity followed this pattern.

We chose to keep this analysis in the Supplementary Information rather than the main manuscript because it addresses a question that is adjacent to, but distinct from, the central hypothesis of the study. The main analyses focus on identifying which behavioral markers predict dyadic performance, whereas the temporal analysis speaks to how partners adapt to one another over the course of an interaction. We agree with the reviewer that this second question is valuable in its own right, and we view a fuller treatment of within-interaction adaptation, including the transition from individual to joint task performance, as a promising direction for future work.

Response to Reviewer 4

Comment 4.1

Overall, the theoretical framework of the study has not yet been sufficiently developed. In particular, there is no systematic integration of the findings from relevant primary studies, which means that the theoretical derivation remains somewhat unclear in places. Greater reference to empirical literature would also enable a clearer derivation of specific hypotheses, which are currently entirely absent. Furthermore, a more precise theoretical foundation would help to better distinguish between the two central approaches, namely internal models as an expectation-based prediction strategy and sensory-based real-time analysis, and to situate them conceptually. It remains unclear in which studies these mechanisms have already been empirically demonstrated, which findings are consistent, and where open research questions still exist. Furthermore, a clearer distinction would be desirable as to whether and to what extent the use of these predictive approaches in joint actions differs systematically depending on the task at hand. Overall, a more in-depth examination of the primary literature would significantly sharpen the theoretical argumentation and considerably strengthen the derivation of the research questions and hypotheses.

Response 4.1

We thank the reviewer for this constructive comment. We agree that the original Introduction did not sufficiently integrate the primary literature and did not make the theoretical basis of our predictions explicit. We have substantially restructured the Introduction to address each of these concerns.

First, we broadened the review of empirical and theoretical work on prediction in joint action. The revised text now traces how predictive accounts have developed across several research traditions, including motor control (Wolpert et al., 1995, 2003), minimal architectures for joint action (Vesper et al., 2010; Sebanz and Knoblich, 2009), the Predictive Joint-Action Model (Pesquita et al., 2018), and the free-energy principle (Friston, 2010; Parr and Friston, 2019). By linking those frameworks to previous empirical findings, we clarify which claims are currently supported by evidence and which questions remain open.

Second, we now draw a clearer distinction between two candidate mechanisms for prediction during coordination. The first is coordination through online extrapolation of currently available sensory information, in which individuals project task-relevant cues from ongoing actions and environmental dynamics, as when a player tracks a ball's trajectory (Bosco et al., 2012; La Scaleia et al., 2014) or a musician extends an unfolding rhythmic pattern (Jones et al., 2017). The second is coordination supported by internal models that generate expectations about upcoming actions

and their sensory consequences, as established in motor control and extended to social interaction through simulation accounts (Wolpert et al., 1995, 2003; Keller et al., 2007; Sabu et al., 2020). For each mechanism we now summarize the supporting empirical evidence to identify questions that remain unresolved.

Third, we added a discussion of how task demands may shape the relative contribution of these mechanisms. We note that highly rhythmic tasks can sustain coordination through mutual coupling between agents, leading to stable phase-locked patterns without explicit anticipation of future actions (Richardson et al., 2007; Schmidt and O'Brien, 1997; Buchanan and Cordova, 2022). Whether such mechanisms are sufficient in naturalistic interactions, where coordination unfolds under irregular timing, changing environmental conditions, and uncertainty about future events, remains an open question. This gap motivates the use of a dynamic, less constrained task in the present study.

Fourth, we now distinguish between prediction of one's own action consequences and prediction of a partner's actions. We discuss the small set of studies that have attempted to separate these contributions, in particular the finding that pianists synchronize more accurately with recordings of their own earlier performances than with recordings by other pianists despite comparable temporal structure (Keller et al., 2007). We explain why the relative contributions of self-prediction and partner-prediction remain largely unresolved, because most paradigms assess only joint outcomes.

Finally, we replaced the previous question-based framing with three explicit hypotheses derived from this revised theoretical foundation:

- **H1:** Anticipatory gaze behavior and motor performance measured during individual-action tasks will predict coordination success during joint action (Hayhoe et al., 2012; Putra and Kano, 2024).
- **H2:** Well-coordinated pairs will differ from less-coordinated pairs in anticipatory gaze behavior and movement dynamics during interaction (Keller et al., 2007; Sacheli et al., 2018).
- **H3:** Successful coordination will depend more strongly on internal-model-based prediction than on online tracking of current sensory evidence alone (Pesquita et al., 2018; Friston, 2010).

In addition, we identify an exploratory question on the relative roles of self-prediction and partner-prediction. We did not formulate a directional hypothesis here because the prior evidence is limited and the two forms of prediction are typically difficult to separate.

Together, these revisions provide a clearer theoretical foundation for the study and strengthen the link between previous literature, the hypotheses, and the analyses.

Comment 4.2

Overall, the discussion is well-structured and presented in a clear and logical manner. At the same time, there are some inconsistencies in the content in certain places, which stem from the theoretical framework. In particular, the findings are sometimes only partially contextualised in relation to existing primary literature, which means that the classification of the results is not consistently refined. A stronger link to established empirical work could have helped to interpret the results in a more nuanced way and to situate them more effectively within the research field. Overall, the discussion remains coherent in its argumentation, but would have gained significantly in analytical depth in some places had the literature been integrated more closely. Deepen the discussion by connecting your results more closely to established empirical studies.

Response 4.2

We thank the reviewer for this suggestion. In the revised Discussion, we more closely connect our findings to previous empirical work on predictive processing, motor control, and joint action.

In particular, we now discuss our findings in relation to studies of anticipatory gaze during interception tasks, predictive adaptation in musical duet performance, and predictive-processing accounts of joint action. We also expanded the discussion of self-prediction versus partner-prediction and considered how the relative importance of predictive, physiological, relational, and demographic factors may vary across coordination contexts.

These additions place the present findings more explicitly within the broader empirical literature and help clarify both their theoretical implications and their relationship to previous work.

First, we situated our findings on anticipatory gaze within the literature on visually guided interception. The result that anticipatory saccades predict coordination success is now discussed in relation to studies showing that skilled performers fixate future contact points well before ball arrival in cricket (Mann et al., 2013) and in laboratory interception tasks (Hayhoe et al., 2012; Diaz et al., 2013; Putra and Kano, 2024). We also point out where our results extend this prior work, namely by showing that gaze patterns previously linked to individual interception performance also carry information about how well two people coordinate, even after controlling for individual skills.

Second, we connected our findings on predictive movement timing to research on musical duet performance. The contribution of pre-contact swing timing to coordination success is now interpreted in light of work showing that pianists adjust their movements based on internal models of the partner's actions (Keller et al., 2007) and that coordination is enhanced when anticipated action consequences match an individual's own sensorimotor expectations. We discuss both the similarities and the differences between rhythmically constrained tasks and the more variable timing of the present task.

Third, we placed our results within current predictive-processing accounts of joint action. We now relate the finding that internal-model-based prediction outweighs online sensory tracking to the Predictive Joint-Action Model (Pesquita et al., 2018), free-energy formulations of social interaction (Friston, 2010; Parr and Friston, 2019), and the proposal that successful interaction depends on internal generative models that anticipate future states and minimize prediction error. We make clear which aspects of our data support these accounts and which remain open.

Fourth, we expanded the comparison between self-prediction and partner-prediction by drawing on the small number of studies that have attempted to separate the two. In particular, we discuss the finding that pianists synchronize more accurately with their own earlier recordings than with recordings by other pianists (Keller et al., 2007), and we relate this to our observation that anticipatory gaze was more closely tied to the consequences of self-generated actions than to partner-generated actions.

Finally, we revised the closing section of the Discussion to consider how the relative weight of predictive, physiological, relational, and demographic factors may vary across coordination contexts. The contrast with studies emphasizing body morphology in joint lifting (Isenhower et al., 2010), interpersonal closeness in joint Simon tasks (Shafaei et al., 2020), and physiological synchrony in conversational and cooperative interactions (Prochazkova et al., 2020; Gordon et al., 2020) is now made explicit.

Comment 4.3

Another key point concerns the composition of the dataset. It remains unclear to what extent individual participants took part in the joint-action condition once or twice, and how these repeated measurements were incorporated into the data structure. This lack of transparency represents a significant limitation, particularly with regard to data analysis. This aspect is not addressed in the discussion or in the section on limitations, even though it means that some individuals may be represented multiple times in the dataset, whilst others contributed to only a single trial run. Particularly in the context of machine learning-based or more complex statistical methods, this represents a potential source of bias that may compromise the independence of the data points.

Response 4.3

We thank the reviewer for raising this important point about the dependence structure of the data. The reviewer is correct that some participants contributed to more than one dyad, and we agree that the manuscript benefits from a clearer description of how the dataset was assembled and how repeated observations were treated in the analyses. We address this in three places in the revised manuscript: the Experimental Procedures, a dedicated methodological subsection, and the Discussion.

1. Clarification of the data collection structure. Each session was scheduled with three participants, with the design intention that each individual would perform the joint-action task twice with two different partners. In 11 of the 34 sessions, one participant cancelled close to the scheduled time, and the session proceeded with the two participants who were present. These reduced sessions therefore yielded a single dyad rather than three. We now state this explicitly in the Experimental Procedures section:

> "Sessions with two participants involved a single joint-action task, producing one dyad. Sessions with three participants involved three joint-action tasks covering all pairwise combinations (AB, AC, and BC), so that each participant completed the joint task twice with two different partners."

2. Treatment of repeated observations across analyses. We added a methodological subsection titled "Handling of repeated individual observations" that describes how the data structure was incorporated into each analysis:

> "As described in the experimental procedures, sessions comprised either two or three participants depending on whether a cancellation occurred (see Supplementary Information). The number of available partners determined whether a given individual contributed to one or two dyads in the joint-action condition.
>
> Analysis 1 was conducted at the dyad level. Each predictor was constructed as a combination of the two individuals' values within a pair, and dyads were treated as the unit of analysis. Because the predictors describe pair-level properties that vary with partner identity, two dyads involving the same participant are not redundant: they correspond to different interaction conditions and produce different predictor values.
>
> Analyses 2 and 3 were conducted at the individual level. Repeated observations from the same participant were accounted for by including by-participant and by-session random intercepts in the models. This specification absorbed individual and session baseline differences into the model and ensured that the estimated effects were not biased by the unequal number of observations per participant."

3. Acknowledgment in the Discussion. Even with the conceptual and statistical safeguards described above, some residual dependence remains, and we now acknowledge this in the limitations section:

> "Some participants contributed to more than one dyad, creating a degree of dependence among observations, although this was accounted for statistically where possible."

Comment 4.4

A set of detailed editorial and presentational suggestions:

- *Emphasize the information in lines 112–114 to help readers understand the variables.*
- *Add explicit hypotheses at lines 120–121.*
- *Define the abbreviation ECG at line 137.*
- *Increase the font size in Figure 1 and fix the erroneous caption text "Abschnitt Eins".*
- *Move the sentence at lines 164–165 about prior eye movement research from the results section to the discussion.*
- *Expand the parameter descriptions in Figure 2 and increase the font size.*
- *Define the abbreviation LF/HF at line 186.*
- *Clarify at lines 218–220 whether the ranking of individual models reflects an analysis result or a literature-based ordering.*
- *Add more description to Figure 3 and increase the font and diagram size.*
- *Reconsider the section heading at line 236 to better match the content.*

Response 4.4

We thank the reviewer for these detailed editorial and presentational suggestions. Each point has improved the clarity of the manuscript and the accessibility of the figures. We respond to each item in turn below.

Emphasis on the variables introduced in lines 112 to 114. We have revised this passage to make the role of each variable more transparent to the reader.

> " Coordination success was defined as performing better together than expected from individual skill alone. We estimated expected performance as the average of the two partners' individual-task scores. Pairs whose joint performance exceeded this value were considered successful, indicating that their coordination produced gains beyond the sum of each person's solo ability. "

We also explain this in Method section.

> " To evaluate joint performance relative to individual skill, we estimated the expected joint-action accuracy for each pair using a Bayesian Gaussian linear model, based on the mean of the pair's individual-action scores. Pairs performing above this expected value were classified as "above-expected," and those below as "below-expected."
>
> "

Explicit hypotheses at lines 120 to 121. We have added explicit hypotheses at the requested location. The revised passage now reads:

> "We addressed three central hypotheses. First, based on previous findings linking anticipatory gaze and motor control to successful performance in ball-interception tasks (Hayhoe et al., 2012; Putra and Kano, 2024), we hypothesized that anticipatory gaze behavior and motor performance measured during individual-action tasks would predict coordination success during joint action. Second, based on studies showing that skilled coordination depends on predictive adaptation between interacting individuals (Keller et al., 2007; Sacheli et al., 2018), we hypothesized that well-coordinated pairs would differ from less-coordinated pairs in both anticipatory gaze behavior and movement dynamics during interaction. Third, based on predictive-processing accounts of joint action (Pesquita et al., 2018; Friston, 2010), we hypothesized that successful coordination would depend more strongly on internal-model-based prediction than on online tracking of current sensory evidence alone.
>
> Our paradigm also allowed us to address an additional question for which we did not make a strong prediction: whether successful coordination is more closely associated with anticipating the consequences of one's own actions or those of a partner. Although both forms of prediction have been implicated in joint action (Wolpert et al., 2003; Keller et al., 2007; Novembre et al., 2014; Sebanz et al., 2006), they are typically difficult to disentangle because they contribute to the same joint outcome. In our task, however, anticipatory gaze following self-generated versus partner-generated hits offered insight into these potentially distinct forms of predictive processing during ongoing coordination."

Definition of ECG at line 137. The abbreviation ECG (electrocardiogram) is now defined on first use. The definition has also been incorporated into the caption of Figure 1 so that readers consulting the figure independently of the main text encounter the full term.

> " During the experiments, participants wore a motion-capture suit, Tobii Glasses 3, and an ECG (electrocardiogram) sensor. "

Figure 1: font size and caption correction. We have increased the minimum font size of all in-figure text from 8 pt to 9 pt to improve legibility. The erroneous caption text "Abschnitt Eins" was a copyright tag for Figure 1a that belongs to the "Abschnitt Eins" company. It has been corrected.

Relocation of the sentence at lines 164 to 165. We agree that this sentence belongs in the Discussion rather than the Results, because it concerns prior literature rather than the present findings. The sentence has been moved to the Discussion and now reads:

> "During object manipulation and ball sports, gaze typically precedes future contact locations, allowing individuals to prepare upcoming actions efficiently Flanagan and Johansson (2003); Land and McLeod (2000); Mann et al. (2013); Hayhoe et al. (2012); Diaz et al. (2013); Putra and Kano (2024)."

Figure 2: expanded parameter descriptions and font size. The minimum in-figure font size has been increased to 9 pt, matching the change applied to Figure 1. We have also expanded the parameter descriptions in the caption so that each variable shown in the figure is defined alongside the panel in which it appears:

> "(a) Participants' typical eye movement and racket maneuvers from a first-person view. The red line represents anticipatory saccades in phase 1, with onset (start of the

line) and offset (end of the line). Green lines indicate corrective saccades occurring after anticipatory saccades, while magenta represents anticipatory saccades in phase 2. Pursuit is shown as squares with a dotted line, and fixations as black-filled circles. (b) Predictors used in this study, categorized into six groups: eye movement, pre-contact swing, contact, heart rate, motion energy, and static characteristics. The eye movement group contains 9 predictors, pre-contact swing 2, contact 3, heart rate 1, motion energy 1, and static characteristics 5. In total, 21 predictors were extracted from three modalities: eye movement, body kinematics, and heart rate."

Definition of LF/HF at line 186. The abbreviation is now defined on first use. The revised sentence reads:

> "Low to high frequency (LF/HF) ratio was used for a heart rate measure."

Clarification of the model ranking at lines 218 to 220. We have clarified that the ranking reflects an empirical result of our analysis rather than an ordering imposed from prior literature. The revised passage reads:

> "Among the single-category models, predictive performance based on accuracy and MCC scores ranked as follows: eye movement, contact behavior, heart rate, pre-contact swing, static characteristics, and movement intensity."

Figure 3: expanded description, font size, and diagram size. The minimum in-figure font size has been increased to 9 pt, and the diagram itself has been enlarged to occupy a larger fraction of the page. The caption has been expanded to provide a fuller description of the elements shown:

> "Test performance of XGBoost models trained on predictor categories from the individual-action task to classify pairs as above- or below-expected in joint-action coordination (n = 64 observations). Predictors included similarity and mean metrics. For the static characteristics category, the mean was computed only for skill, whereas the remaining static predictors are represented as between-partner differences. Accuracy (a), and the Matthews Correlation Coefficient (MCC) (b), were estimated on held-out test data using three-fold cross-validation. Results are ordered from lowest (top) to highest (bottom). Circles indicate the scores on the test data, error bars indicate 95% bootstrap confidence intervals centered on the point estimates, and scatter points represent the distribution of scores across individual bootstrap samples. The 'Full model' combines all predictor categories."

Section heading at line 236. We agree that the previous heading did not fully convey the content of the section. It has been revised to:

> "Analysis 2: Behaviors distinguishing well- and less-coordinated pairs"

This new heading reflects the focus of the section on the behavioral contrasts between the well-coordinated dyads and the less-coordinated dyads during joint action.

Response to Reviewer 5

Comment 5.1

Change the title. There is a considerable gap between the research content and the current title. The study focuses primarily on coordination in motor tasks, whereas the current title covers a much broader scope.

Response 5.1

We agree that the previous title implied a scope broader than the empirical focus of the manuscript, which centers on coordination within a dynamic motor interaction. The revised title now reflects both the paradigm employed and the principal theoretical claim of the study:

> **"Successful coordination emerges from aligned self-predictions in a dynamic motor interaction"**

This formulation explicitly identifies the behavioral context (a motor interaction) and the proposed mechanism (alignment of self-predictions between partners), and so it aligns the title with the content of the manuscript.

Comment 5.2

In the introduction, could the authors derive specific hypotheses based on existing theories or previous studies? If not, please explain why specific hypotheses were not proposed.

Response 5.2

In the revised manuscript, we now derive three explicit hypotheses from previous empirical and theoretical work and present them at the end of the Introduction.

> " The first hypothesis predicts that anticipatory gaze behavior and motor performance measured during individual-action tasks will predict coordination success during joint action. The second predicts that well-coordinated pairs will differ from less-coordinated pairs in anticipatory gaze behavior and movement dynamics during interaction. The third predicts that successful coordination will depend more strongly on internal-model-based prediction than on online tracking of current sensory evidence alone.
>
> In addition, we identify an exploratory question concerning whether successful coordination is more closely associated with prediction of one's own action consequences or those of a partner. This question was not associated with a strong a priori prediction, because existing literature implicates both forms of prediction in joint action.
> "

Comment 5.3

Sample size. The authors have already provided a justification for the sample size. However, given that this study involves relatively complex and multiple statistical analysis methods, I suggest briefly discussing this in the limitations section.

Response 5.3

We thank the reviewer for this constructive suggestion. We agree that the combination of a moderate sample size with multiple analyses of differing complexity warrants explicit discussion. To address this concern transparently, we have taken two complementary steps in the revised manuscript.

First, in the Methods section, we describe how the analytical pipeline accommodates the structural complexity of the dataset. In particular, the subsection "Handling of repeated individual observations" specifies the unit of analysis used in each model and the random-effects structure employed for the individual-level analyses.

Second, in the Discussion, we have added a limitation paragraph that links sample size to interpretive scope. The paragraph notes that some participants contributed to more than one dyad and that the study may have been less sensitive to smaller effects. The revised text reads:

> "A further potential limitation concerns statistical inference. Some participants contributed to more than one dyad, creating a degree of dependence among observations, although this was accounted for statistically where possible. In addition, while the sample was sufficient to detect the strong contribution of prediction to coordination success, it may have been less sensitive to smaller effects associated with demographic, physical, physiological, and relational variables. Consequently, the limited contributions of these factors should be interpreted cautiously until replicated in larger samples."

Comment 5.4

Data structure. I noticed that sometimes three participants are tested simultaneously, and then paired in different combinations for the coordination task. Does this involve a situation where one participant takes part in two coordination tasks? Or do all participants complete two coordination tasks? My question is whether this differs from a two-person simultaneous experiment, and whether this could affect the data analysis results.

Response 5.4

We thank the reviewer for raising this point, which closely parallels a concern expressed by Reviewer 4. Because both reviewers identified the same issue, we have addressed it once and comprehensively in two locations of the revised manuscript.

In the Methods, we have added the subsection "Handling of repeated individual observations". This subsection clarifies that three-participant sessions yielded three dyads covering all pairwise combinations, whereas two-participant sessions (occurring when one participant cancelled near the scheduled time) yielded a single dyad. It also specifies the analytical strategy for each of the three analyses: Analysis 1 was conducted at the dyad level with the dyad treated as the unit of analysis, while Analyses 2 and 3 were conducted at the individual level with by-participant and by-session random intercepts to absorb baseline differences and to prevent bias arising from the unequal number of observations per participant.

In the Discussion, we have added a limitation paragraph that acknowledges the residual dependence remaining in the data and frames the implications for interpretation and generalizability.

We refer the reviewer to our response to Reviewer 4, Comment 4.3, for the full text of the revised passages.

Comment 5.5

Expand the discussion. The authors could briefly discuss the mechanisms underlying coordination activities that differ from table tennis to help stimulate readers' further thinking.

Response 5.5

We thank the reviewer for this suggestion, which has improved the conceptual scope of the Discussion. We have added a paragraph that situates the present findings within the broader literature on joint action and identifies the conditions under which the mechanisms emphasized here may differ from those operative in other coordination contexts. The added text reads:

> "Our results indicated that, while prediction appeared to play an important role, demographic, physical, physiological, and relational variables contributed relatively little to coordination success in our task. This pattern contrasts with findings from other joint-action paradigms in which body morphology constrains physically demanding tasks such as joint lifting Isenhower et al. (2010), interpersonal closeness modulates shared action representations in joint Simon tasks Shafaei et al. (2020), or physiological synchrony contributes to conversational and cooperative interactions unfolding over longer timescales Prochazkova et al. (2020); Gordon et al. (2020). One possible explanation is that the relative importance of coordination mechanisms depends on the temporal and sensorimotor demands of the task. In rapidly unfolding interactions such as the present ball-exchange task, where participants must continuously anticipate future action outcomes on a sub-second timescale, coordination may rely more heavily on anticipatory control. In contrast, slower or more socially mediated interactions might allow greater contributions from interpersonal, physiological, or relational alignment processes."

This expanded passage articulates a testable framework for how coordination mechanisms may scale with the temporal and sensorimotor demands of the task, and it provides the reader with concrete points of comparison across the existing literature.

References

Aas, K., Jullum, M., and Løland, A. (2021). Explaining individual predictions when features are dependent: More accurate approximations to shapley values. *Artificial Intelligence*, 298:103502.

Bosco, G., Delle Monache, S., and Lacquaniti, F. (2012). Catching what we can't see: manual interception of occluded fly-ball trajectories. *PLoS One*, 7(11):e49381.

Buchanan, J. J. and Cordova, A. (2022). Individual goals interact with dyad goals to constrain and facilitate the formation of interpersonal patterns of coordination. *Human Movement Science*, 83:102949.

Diaz, G., Cooper, J., Rothkopf, C., and Hayhoe, M. (2013). Saccades to future ball location reveal memory-based prediction in a virtual-reality interception task. *Journal of vision*, 13(1):20–20.

Flanagan, J. R. and Johansson, R. S. (2003). Action plans used in action observation. *Nature*, 424(6950):769–771.

Friston, K. (2010). The free-energy principle: a unified brain theory? *Nature reviews neuroscience*, 11(2):127–138.

Gordon, I., Gilboa, A., Cohen, S., Milstein, N., Haimovich, N., Pinhasi, S., and Siegman, S. (2020). Physiological and behavioral synchrony predict group cohesion and performance. *Scientific Reports*, 10:8484.

Hayhoe, M. M., McKinney, T., Chajka, K., and Pelz, J. B. (2012). Predictive eye movements in natural vision. *Experimental brain research*, 217:125–136.

Isenhower, R. W., Richardson, M. J., Carello, C., Baron, R. M., and Marsh, K. L. (2010). Affording cooperation: Embodied constraints, dynamics, and action-scaled invariance in joint lifting. *Psychonomic Bulletin & Review*, 17(3):342–347.

Jones, A., Hsu, Y.-F., Granjon, L., and Waszak, F. (2017). Temporal expectancies driven by self-and externally generated rhythms. *NeuroImage*, 156:352–362.

Keller, P. E., Knoblich, G., and Repp, B. H. (2007). Pianists duet better when they play with themselves: on the possible role of action simulation in synchronization. *Consciousness and cognition*, 16(1):102–111.

La Scaleia, B., Lacquaniti, F., and Zago, M. (2014). Neural extrapolation of motion for a ball rolling down an inclined plane. *PLoS One*, 9(6):e99837.

Land, M. F. and McLeod, P. (2000). From eye movements to actions: how batsmen hit the ball. *Nature neuroscience*, 3(12):1340–1345.

Mann, D. L., Spratford, W., and Abernethy, B. (2013). The head tracks and gaze predicts: how the world's best batters hit a ball. *PLoS One*, 8(3):e58289.

Novembre, G., Ticini, L. F., Schütz-Bosbach, S., and Keller, P. E. (2014). Motor simulation and the coordination of self and other in real-time joint action. *Social cognitive and affective neuroscience*, 9(8):1062–1068.

Parr, T. and Friston, K. J. (2019). Generalised free energy and active inference. *Biological cybernetics*, 113(5):495–513.

Pesquita, A., Whitwell, R. L., and Enns, J. T. (2018). Predictive joint-action model: A hierarchical predictive approach to human cooperation. *Psychonomic Bulletin & Review*, 25:1751–1769.

Prochazkova, E., Sjak-Shie, E., Behrens, F., Lindh, D., and Kret, M. E. (2020). Physiological synchrony is associated with cooperative success in real-life interactions. *Scientific Reports*, 10:19078.

Putra, P. U. and Kano, F. (2024). Quantifying the effect of anticipatory eye movement on successful ball hitting using fine-scale tracking and shap-analysis. In ETRA 2024 Organizing Committee, editor, *Proceedings of the 2024 ACM Symposium on Eye Tracking Research & Applications*, pages 1–9, Glasgow, United Kingdom. ACM.

Richardson, M. J., Marsh, K. L., Isenhower, R. W., Goodman, J. R., and Schmidt, R. C. (2007). Rocking together: Dynamics of intentional and unintentional interpersonal coordination. *Human movement science*, 26(6):867–891.

Sabu, S., Curioni, A., Vesper, C., Sebanz, N., and Knoblich, G. (2020). How does a partner's motor variability affect joint action? *PLOS ONE*, 15(10):e0241417.

Sacheli, L. M., Arcangeli, E., and Paulesu, E. (2018). Evidence for a dyadic motor plan in joint action. *Scientific reports*, 8(1):5027.

Schmidt, R. C. and O'Brien, B. (1997). Evaluating the dynamics of unintended interpersonal coordination. *Ecological Psychology*, 9(3):189–206.

Sebanz, N., Bekkering, H., and Knoblich, G. (2006). Joint action: bodies and minds moving together. *Trends in cognitive sciences*, 10(2):70–76.

Sebanz, N. and Knoblich, G. (2009). Prediction in joint action: What, when, and where. *Topics in cognitive science*, 1(2):353–367.

Shafaei, R., Bahmani, Z., Bahrami, B., and Vaziri-Pashkam, M. (2020). Effect of perceived interpersonal closeness on the joint simon effect in adolescents and adults. *Scientific Reports*, 10(1):18107.

Vesper, C., Butterfill, S., Knoblich, G., and Sebanz, N. (2010). A minimal architecture for joint action. *Neural networks*, 23(8-9):998–1003.

Wolpert, D. M., Doya, K., and Kawato, M. (2003). A unifying computational framework for motor control and social interaction. *Philosophical Transactions of the Royal Society of London. Series B: Biological Sciences*, 358(1431):593–602.

Wolpert, D. M., Ghahramani, Z., and Jordan, M. I. (1995). An internal model for sensorimotor integration. *Science*, 269(5232):1880–1882.

Response to Reviewers (Second Revision)

Dear Editor,

Thank you again for handling our manuscript and for giving us the opportunity to submit a further revised version. We are grateful to the reviewers for their continued time and for their positive assessment of our previous revision. We have carefully considered the remaining comments and respond to each one below.

All new changes in the manuscript have been highlighted in yellow.

Response to Reviewer 1

Comment 1.1

Thank you for this. You have clarified all of my concerns. Great work!

Response 1.1

We thank the reviewer for the detailed feedback throughout the review process and for the positive assessment of our revisions.

Response to Reviewer 4

General comment. The revised manuscript has improved substantially compared to the previous version. In particular, the theoretical framing has been strengthened, and several of the concerns raised in the initial review have been addressed. Nevertheless, given the complexity of the study design and the analyses performed, some aspects would benefit from further clarification before publication.

Comment 4.1

Hypotheses and operationalisation (Lines 135–145). *It is very helpful that explicit hypotheses have now been added at the end of the Introduction. Given the complexity of the analyses, I would additionally recommend linking each hypothesis more directly to the variables used in the analyses. Specifically, it would be helpful to clarify how the conceptual hypotheses are translated into the empirical models (i.e., which independent and dependent variables are expected to be related, and in what direction). This would make the analytical approach easier for readers to follow.*

Response 4.1

We thank the reviewer for this helpful suggestion. We agree that the link between each conceptual hypothesis and its empirical model should be explicit. We have revised the analysis framework under Methods section so that each hypothesis now opens with the question it addresses and states the variables involved, including the dependent measure and the predictors expected to relate to it. The revised passages read as follows.

> " To test the first hypothesis, we trained an XGBoost classifier Chen and Guestrin (2016) to predict a pair's performance in the joint-action task using several measures collected during the individual-action task: eye-movement behavior, pre-contact swing and contact, heart rate, and movement energy. We also added static participant characteristics. The classifier's response variable was whether the pair performed above or below the level expected from their two individual scores. Because the model weighs all of these measures together, it lets us ask not only whether anticipatory gaze behavior and motor response measured during the individual-action task carry information about how well a pair coordinates in the joint-action task, but also how important they are relative to the other variables. "

> " The second hypothesis concerns whether coordination quality is visible in behavior during the interaction itself. To test it, we compared well-coordinated and less-coordinated pairs on their anticipatory gaze behavior and movement dynamics during the joint-action task, together with their static characteristics. We tested these group differences using Bayesian t-tests Kruschke (2013), which quantify uncertainty and allow each difference to be interpreted in probabilistic terms. "

> " The third hypothesis asks what drives accurate gaze anticipation during successful coordination. To address it, we fitted a hierarchical Bayesian generative model to each participant's Phase-1 visual-angle error in the joint-action task (y_f). The model expressed this error in terms of the participant's prior expectation (P) and the incoming sensory evidence ($\hat{X}$), allowing us to examine the relative importance of each. "

Comment 4.2

Sample structure and repeated participation (Lines 187–191). *The revised manuscript now provides a clearer explanation that some participants contributed data in more than one dyad, which is a welcome addition. However, it would be helpful to report the exact numbers of participants who were included in one dyad versus those who participated in two dyads. This would further improve transparency regarding the dataset structure.*

Response 4.2

We thank the reviewer for this suggestion. We have added a new table to the Supplementary Information, "Table S4. Sessions per participant," which lists, for each participant, the sessions they took part in and how many dyads they contributed to.

Comment 4.3

Table tennis expertise. *In the Results section, it remains unclear whether differences associated with table tennis expertise were examined. Given the nature of the task, it would be useful to clarify whether expertise was analyzed and, if so, whether it influenced any of the reported outcomes.*

Response 4.3

We thank the reviewer for raising this point. Table tennis expertise was indeed considered, and our analysis was designed to account for it directly.

Rather than modeling the raw joint-action score, we defined our outcome in a way that removes baseline skill from the start. For each pair, we estimated the expected joint-action score from the two partners' individual scores, and then classified pairs by whether they performed above or below that expected value. Because this outcome is defined relative to individual skill, differences in expertise are already controlled for before any predictor is tested.

We also examined skill more directly. In Analysis 1, we included the mean skill and the between-partner difference in skill as predictors, and in Analysis 2 we compared these same measures between the above- and below-expected groups; where in both analyses these predictors contributed minimally. Moreover, we now make this explicit in the Results section:

> " On average, participants successfully hit the ball back in 90.82% (SD = 10.53%) of events during the individual-action task, and in 85.21% (SD = 11.33%) of events during the joint-action task. To examine joint performance relative to individual skill, we estimated the expected joint-action skill for each pair using a Bayesian Gaussian linear model, based on the mean of the pair's individual-action scores. Pairs performing above this expected value were classified as 'above-expected,' and those below as 'below-expected.' Because this classification is defined relative to individual skill, baseline expertise is already accounted for before any predictor is evaluated. To examine skill effects further, we also included the mean and between-partner difference in skill as predictors in Analysis 1 and compared them between groups in Analysis 2. "

Comment 4.4

Discussion and hypotheses. *I recommend returning more explicitly to the hypotheses at the beginning of the Discussion. The interpretation of the findings would benefit from a clearer structure that systematically addresses whether and how the individual hypotheses were supported by the results.*

Response 4.4

We agree with the reviewer. We have restructured the opening of the Discussion so that each hypothesis is stated first, followed by the results that bear on it and a statement of whether it was supported. The three hypotheses are now addressed in the same order in which they were introduced. The revised passages open as follows:

> " We tested whether anticipatory gaze behavior and motor performance predict successful coordination. Using multimodal behavioral and physiological measurements together with machine learning and Bayesian modeling, . . . "

> " As hypothesized, well-coordinated and less-coordinated pairs differed in both anticipatory gaze behavior and movement dynamics during interaction. Well-coordinated pairs showed greater anticipatory control . . . "

> " Our Bayesian generative model quantified the relative contributions of prior expectations and incoming sensory evidence to prediction error during joint-action task. Well-coordinated pairs . . . "

Minor comments

Comment 4.5

- *Line 103: The parenthesis in "(e.g., ... " is not closed.*
- *Line 157: Please also report the age range of the participants.*

Response 4.5

The parenthesis has been corrected and the range of participants has been added to under Participants and Datasets section.

> " A total of 91 participants (36 female, 54 male, and one non-binary; mean age = 25.29 years, SD = 7.09, range = 19–45) "

Response to Reviewer 5

Comment 5.1

The authors have adequately addressed my comments and concerns. I appreciate their careful revisions and wish them the best of luck with the manuscript.

Response 5.1

We thank the reviewer for the detailed feedback throughout the review process and for the positive assessment of our revisions.

References

Chen, T. and Guestrin, C. (2016). Xgboost: A scalable tree boosting system. In KDD 2016 Organizing Committee, editor, *Proceedings of the 22nd ACM SIGKDD International Conference on Knowledge Discovery and Data Mining*, pages 785–794, San Francisco, CA, USA. ACM.

Kruschke, J. K. (2013). Bayesian estimation supersedes the t test. *Journal of Experimental Psychology: General*, 142(2):573.
