# Supplementary material for: Successful coordination emerges from aligned self-predictions in a dynamic motor interaction
Source: Commun Psychol. 2026 Aug 20;4:119. doi: 10.1038/s44271-026-00523-7 (PMC13493972; doi:10.1038/s44271-026-00523-7)
Supplement: Supplementary file 2 [file 44271_2026_523_MOESM2_ESM.pdf]

Supplementary Information for

Successful coordination emerges from aligned self-predictions in a dynamic motor interaction

Prasetia Utama Putra and Fumihiro Kano

Prasetia Putra.
E-mail: prasetiautama2@gmail.com, prasetia.putra@uni-konstanz.de, pputra@ab.mpg.de
Fumihiro Kano
E-mail: fkanou@gmail.com, fumihiro.kano@uni-konstanz.de, fkano@ab.mpg.de

This PDF file includes:

Supporting text
Figs. S1 to S19
Tables S1 to S6
Legends for Movies S1 to S2
SI References

Other supporting materials for this manuscript include the following:

Movies S1 to S2

Supporting Information Text

Analysis 1: Additional results

Misclassified instances. Using all predictor categories, the XGBoost model achieved an Area Under the Curve (AUC) of 0.72 (95% CI: [0.71, 0.74]). A total of 19 out of 64 instances were misclassified (Figure S5).

Model comparison results. Comparison between linear and non-linear models using bagging and boosting algorithms (Figure S6) revealed that the boosting algorithm (XGBoost) outperformed the others, as indicated by higher accuracy and Matthews Correlation Coefficient (MCC) scores. In contrast, the linear model (logistic regression) yielded the lowest performance, with the lowest accuracy and MCC values among the three approaches.

Bayesian t-test for top-15 predictors based on SHAP-value. Bayesian t-tests on the top 15 predictors (Figure S8) revealed significant differences, based on Wasserstein distance, in the visual angle error of anticipatory saccades in Phase 1, the mean number of corrective saccades in Phases 1 and 2, and the mean difference in distance. No significant differences were observed for the remaining predictors.

XGBoost performance with top features. Comparison of accuracy and MCC scores for the XGBoost models (Figure S8) shows that the model using the top 2 predictors achieved the highest performance. In contrast, the full model as well as models using only the top 1 or top 5 predictors showed similarly lower performance compared to the top-2 model.

Variance inflation and PCA analysis. The variance inflation analysis S1 revealed substantial multicollinearity among several predictors, particularly for subject skill, corrective saccades, and pre-contact swing onset. The highest VIF values were observed for the mean and difference measures of subject skill. Mean-based coordination and gaze-control variables generally showed higher collinearity than difference-based variables, suggesting that shared dyadic performance characteristics overlap more strongly than interpersonal asymmetries.

To verify the robustness of the XGBoost model against multicollinearity, a complementary analysis was conducted using Principal Component Analysis (PCA). The predictors from Analysis 1 were normalized and reduced to latent variables. Four configurations were tested, using 2, 5, 10, and 15 components, and model performance was evaluated with accuracy and the Matthews correlation coefficient (MCC). Hyperparameters were optimized using the Optuna framework. Within this design, superior performance with latent variables would indicate that XGBoost alone cannot adequately mitigate multicollinearity in the dataset.

The PCA-based models S2 achieved 15.63% lower accuracy and 0.31 lower MCC than the models trained on the original predictors. This result indicates that XGBoost effectively handles multicollinearity in the present data, whereas dimensionality reduction discards information relevant to classification and degrades model performance.

Analysis 2: Additional results

Sensitivity analysis. A comparison between the Skew-StudentT and StudentT models (Figure S13a) in Analysis 2 shows that the Skew-StudentT consistently achieves better predictive performance, with LOO differences exceeding 10 in both above- and below-expected groups. Small negative LOO differences were observed for pre-contact swing onset in the below-expected group (–0.6) and racket spatial control in the above-expected group (–1.5). However, these small values suggest negligible performance differences in these cases.

Models with narrower priors for subject and session intercepts ($N(0, 0.1)$) outperformed those with wider priors ($N(0, 0.5)$), as indicated by positive LOO differences across both groups. Small negative LOO differences appeared for the number of corrective saccades in Phase 2 for both the hitter (–6.0) and receiver (–5.7) in the above-expected group, and for motion energy (–0.1) and skill (–1.2) in the below-expected group. Again, the small magnitudes indicate that the performance gaps are negligible in these specific comparisons.

Models convergence. Effect size samples and R-hat values (Figure S14) from the Bayesian models in Analyses 2 and 3 indicated good convergence across all models, supporting the reliability and robustness of the results. One exception was the model estimating the magnitude of the hitter's anticipatory saccades in Phase 1, which had an R-hat value close to 1.02. However, its effective sample sizes (ESS) remained adequate, with ess-bulk = 442 and ess-tail = 937, suggesting that the model still achieved acceptable convergence.

Insignificant predictors. No significant differences were observed in static characteristics, motion intensity, heart rate, and several eye-movement and contact-related predictors (Figure S15). However, distinct patterns emerged in three variables: the hitter's anticipatory saccade onset in Phase 1, the receiver's pursuit duration in Phase 3, and the age difference between participants. In the above-expected group, hitters initiated their anticipatory saccades later after hitting the ball and receivers pursued the ball for a shorter duration before striking it. Additionally, participants in this group had a larger age difference compared to those in the below-expected group.

Change of behavior. We examined how participants' behaviors evolved over time using a longitudinal Bayesian model (n windows = 10). To account for the distributional characteristics of the data, we modeled continuous responses using a Skewed Student's t-distribution and discrete count data—specifically, the number of corrective saccades—using a Poisson distribution.

Two distinct temporal patterns emerged from the analysis (Figure S16). Some predictors revealed stable group-level differences from the outset that remained consistent across time. These included the number of corrective saccades in Phase 1 for both hitters and receivers, as well as the timing of the pre-contact swing. In contrast, other predictors—such as pair-centered deviation and post-hit table proximity—initially exhibited comparable values between groups but diverged gradually as the task progressed.

These findings suggest that well-coordinated pairs were equipped with distinct predictive mechanisms from the beginning, enabling them to anticipate and optimize their actions under temporal constraints. Notably, these mechanisms appeared resilient to short-term adaptation, implying that successful coordination in fast paced task may depend on similar internal models rather than rapid trial-based learning. Conversely, the divergence observed in less-coordinated pairs suggests that they struggled to maintain consistent spatial alignment during the rally, as evidenced by increasing deviations in ball bounce proximity and ineffective repositioning after hitting. This failure to sustain coordination may reflect less robust predictive control, reinforcing the idea that anticipatory mechanisms play a critical role in dynamic joint action.

Analysis 3: Additional results

Change overtime of w . To examine the temporal dynamics of sensory-evidence weighting, we divided each session into four segments: Segment 1 (events 1–15), Segment 2 (16–30), Segment 3 (31–50), and Segment 4 (51 to the end). The results (Figure S17) show no significant change in sensory-evidence weight across segments for the below-expected group. In contrast, the above-expected group exhibited a moderate decline in weighting during Segments 3 and 4. This pattern suggests that, over time, individuals in the above-expected group relied less on incoming sensory evidence and more on prior expectations, indicating a shift toward more automatic behavior in the later phases of the task.

Extra analysis

Difference in behavior: individual-action vs joint-action tasks. We examined how participants in the above-expected group changed their behavior between the individual-action and the joint-action task, compared to those in the below-expected group. For each predictor, we calculated the differences in both mean and variance (standard deviation) between the two task conditions. A positive mean difference indicated a higher value in the joint-action task relative to the individual-action task, while a positive variance difference reflected greater variability during joint action (Figure S18 **a, b**).

The only significant differences we observed were in pre-contact swing timing and ball control variability. Individuals in the above-expected group initiated their pre-contact swings earlier during the joint-action task compared to the individual-action task. They also exhibited lower variability in both ball speed control (i.e., smaller changes in ball speed before and after contact) and ball spatial control (i.e., smaller deviations between the wrist-to-racket centroid and the contact point).

Self-assessment of performance as predictors. After completing the tasks, participants rated their performance on questionnaires assessing four domains: their own performance, their team's performance, their role as facilitator during joint-action, and their performance in the individual-action task. Ratings were given on a 7-point scale.

We used Bayesian t-tests with an ordered probit response distribution to compare questionnaire responses between individuals in the above- and below-expected groups. No significant differences emerged (Figure S19). However, descriptive trends indicated that participants in the above-expected group tended to rate their own, their partner's, and their team's performance more positively than those in the below-expected group.

In summary, while some individuals reported perceiving higher levels of joint-action performance, these self-assessments did not provide a reliable predictor of actual task performance in the experimental setting.

Materials and Methods

Materials. A machine learning algorithm typically requires an effect size of 0.5 to achieve robust accuracy in two-class classification (1). Here, we used Bayesian estimation to determine the sample size needed to detect this effect reliably. Assuming one observation per session (two individuals forming one dyad), a feature correlation of 0.5, and within-individual differences, we simulated datasets with number of dyads ranging from 10 to 100 sessions. The results (Figure S1) indicated that a minimum of 20 sessions is needed to detect an effect size of 0.5 with a median p-value below 0.01. To account for potential data loss and observational noise, we collected data from 71 sessions in this study.

Methods.

Data measurement and pre-processing. Using a motion capture system integrated with wearable eye-tracking, we quantified the kinematics of the ball, racket, participants' body, and gaze direction, as well as the position of the wall and table (Figure S2). Each participant wore a motion suit with 37 markers, which enabled reconstruction of 19 body segments. To track the racket, wall, and table, we attached four markers to each object. The racket markers were arranged differently to distinguish between rackets. Gaze direction was measured with Tobii Glasses 3, which we synchronized with the motion capture system using

two sets of reflective markers (Tobii segment) placed on the left and right sides of the glasses. The global coordinates of gaze vectors were computed by transforming local gaze coordinates into the global reference frame using the following formula:

$$\begin{aligned}&\hat{P} = (T \cdot P_{\text{local}}) + \overrightarrow{t}\\ &T \in \mathbb{R}^{4 \times 4}\\ &P_{local} \in \mathbb{R}^{3}\\ &\overrightarrow{t} \in \mathbb{R}^{3}\end{aligned} \qquad [1]$$

where P_{local} denotes the gaze vector in local coordinates, T is a rotation matrix derived from the orientation of the Tobii segment, and $\overrightarrow{t}$ is the translation vector representing the segment's global position.

We applied modality-specific preprocessing techniques to remove noise and interpolate missing data (Figure S2 b). Following preprocessing, we performed a data sanity check to ensure consistency before extracting relevant predictors.

Quantification of eye-movement predictors. After transforming the local gaze vector into the global coordinate system, we extracted three predictors from the gaze data (Figure S3a): anticipatory saccades, corrective saccades, and smooth pursuit. Saccades were detected based on the velocity and acceleration of the gaze vector, and subsequently classified as either anticipatory or corrective (Figure S3b). Smooth pursuit during Phase 3 was identified by integrating gaze velocity and acceleration with the distance between the gaze point and the ball trajectory in spherical coordinates (Figure S3c).

Bayesian GLM to categorize pairs. The categorization into above-expected and below-expected groups was derived using a Bayesian Gaussian Linear Model (GLM) (Eq. 2). In this model, participants' success scores in individual-action tasks served as a linear predictor of their performance in joint-action tasks:

$$\begin{aligned}&y \sim T(3, \mu, \sigma)\\ &\mu = \alpha + \beta * X\\ &\alpha \sim N(0, 5)\\ &\beta \sim N(0, 5)\\ &\sigma \sim HalfCauchy(10)\end{aligned} \qquad [2]$$

where X represents individuals' success percentage in individual-action tasks, and y denotes pairs' success percentage in joint-action tasks. Model posteriors were estimated using U-Turn Hamiltonian Monte Carlo sampling (2), with hyperparameters detailed in Table S6.

XGBoost hyperparameters (Analysis 1). For each feature category, we optimized the hyperparameter values using all available data, with accuracy as the scoring metric (Table S5). The learning_rate controls how much each decision tree contributes to the final prediction, balancing learning speed and stability. The subsample parameter determines the fraction of the training data randomly selected for each tree, which helps prevent overfitting. The max_depth defines the maximum depth of each decision tree, influencing model complexity. The alpha term serves as an L1 regularization on leaf weights to reduce overfitting. The min_child_weight specifies the minimum sum of instance weights (hessians) required in a child node, acting as a constraint on further partitioning. Finally, the max_delta_step limits how much a leaf node's prediction can change in one boosting round, improving convergence stability.

Hierarchical model structure (Analysis 1). We used hierarchical models (Figure S9) to account for variation across participants and sessions. This approach allows us to estimate uncertainty at multiple levels of the data structure. The random intercepts were modeled for 74 participants and 64 sessions, corresponding to the total number of individuals and sessions included in the analysis.

Priors in Analysis 2 and 3. In total, we used four types of likelihood distributions to model the response variables: Skew-StudentT, StudentT, Poisson, and Categorical. The priors used for each distribution are as follows:

1. Prior specific for Skew-StudentT

$$\begin{aligned}&y \sim T(a, b, \mu, \sigma)\\ &a \sim Gamma(2, 0.5)\\ &b \sim Gamma(2, 0.5)\\ &\mu_k \sim N(\overline{x}, 1) + \alpha\\ &\sigma_l \sim HalfCauchy(1)\end{aligned} \qquad [3]$$

2. Prior specific for StudentT

$$\begin{aligned} &y \sim T(v, \mu, \sigma) \\ &v_u \sim Exp(1/29) \\ &v_u \sim Deterministic(v_u + 1) \\ &\mu_k \sim N(\overline{x}, 1) + \alpha \\ &\sigma_l \sim HalfCauchy(1) \end{aligned} \quad [4]$$

3. Prior specific for Possion

$$\begin{aligned} &y \sim Poisson(\mu) \\ &\mu_k \sim HalfNormal(1) + \alpha \end{aligned} \quad [5]$$

4. Prior specific for Categorical

$$\begin{aligned} &y \sim Categorical(\mu) \\ &\mu_k \sim N(\overline{x}, 1) + \alpha \end{aligned} \quad [6]$$

5. Prior for interceptions

$$\begin{aligned} &\alpha_{\text{session}} = \sum_{k=0}^{M} \alpha_{\text{sessions},k} \quad \forall k \in \text{Sessions} \\ &\alpha_{\text{subject1}} = \sum_{j=0}^{N} \alpha_{\text{subjects},j} \quad \forall j \in \text{Subjects} \\ &\alpha_{\text{subject2}} = \sum_{j=0}^{N} \alpha_{\text{subjects},i} \quad \forall i \in \text{Subjects} \\ &\alpha_{\text{sessions}} \sim N(0, 0.1) \\ &\alpha_{\text{subjects}} \sim N(0, 0.1) \end{aligned} \quad [7]$$

6. Prior for Poisson interception

$$\begin{aligned} &\alpha_{\text{sessions}} \sim HalfNormal(0.1) \\ &\alpha_{\text{subjects}} \sim HalfNormal(0.1) \end{aligned} \quad [8]$$

Model posteriors were estimated using U-Turn Hamiltonian Monte Carlo sampling (2), with hyperparameters detailed in Table S6.

Measuring Cohen's d in Bayesian t-test (Analysis 2). Cohen's d (effect size) for the Skew-StudentT and StudentT models was calculated using the following formula:

$$d = \frac{\mu_1 - \mu_2}{\sqrt{0.5 * (\sigma_1^2 + \sigma_2^2)}} \quad [9]$$

where μ_1 and μ_2 represents the means distribution of above-and below-expected groups, and σ_1 and σ_2 their corresponding standard deviation.

For the Poisson model, the effect size was computed as (3):

$$\begin{aligned} &d = \frac{\mu_1 - \mu_2}{\sqrt{0.5 * (\sigma_1 + \sigma_2)}} \\ &\sigma_1 = \mu_1 \\ &\sigma_2 = \mu_2 \end{aligned} \quad [10]$$

Bayesian generative model structure (Analysis 3). To model how individuals used their prior information relative to sensory evidence when generating predictions about the environment in which they coordinated, we employed a Hierarchical Bayesian Generative Model with the following structure:

1. Prior specific for subject_intercept

$$\alpha_{\text{subject}} \sim N(\hat{\mu}, \hat{\sigma}), \quad [11]$$

where $\hat{\mu}$ and $\hat{\sigma}$ represent mean and variance of Phase-1 visual angle error measured in individual-action task.

2. Prior specific for session intercept

$$\alpha_{\text{session}} \sim N(0, 1) \quad [12]$$

3. Prior specific for beta sensory

$$\beta \sim N(0, 1) \quad [13]$$

4. Prior specific for variance of prior expectations

$$\sigma^2_{\text{prior}} \sim exp(N(0, 0.5)) \quad [14]$$

5. Prior specific for variance of sensory evidence

$$\sigma^2_{\text{sensed}} \sim exp(N(0, 0.5)) \quad [15]$$

6. Prior specific for Skew-Student-T

$$\begin{aligned} &y \sim T(a, b, \mu, \sigma) \\ &a \sim Gamma(2, 0.5) \\ &b \sim Gamma(2, 0.5) \\ &\mu = (1 - w)\, P + w\, \hat{X} \\ &\sigma_l \sim HalfCauchy(1) \end{aligned} \quad [16]$$

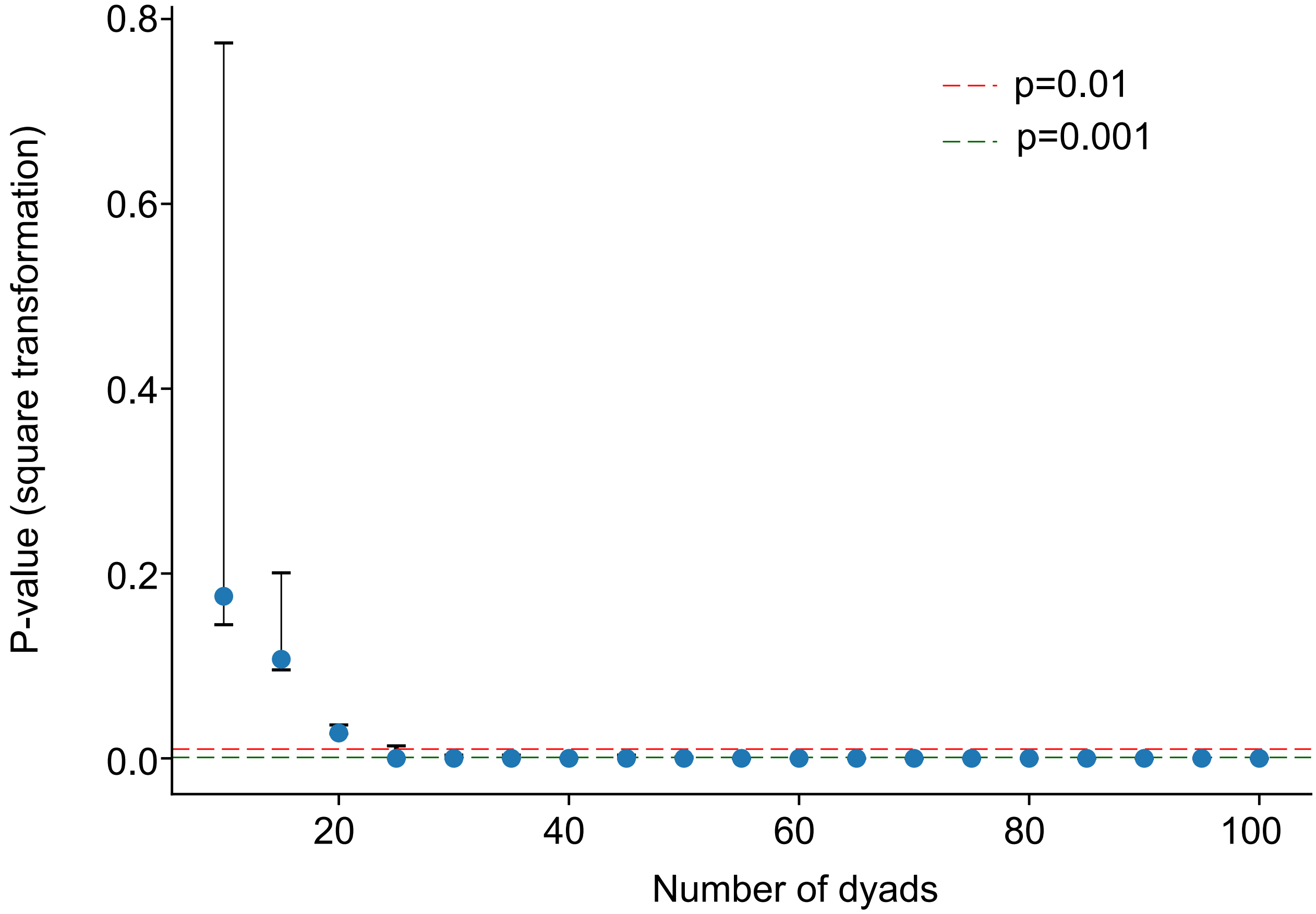

Fig. S1. Estimation of p-values across different sample sizes. Circles represent the median p-value, and error bars indicate the 95% highest density interval (HDI).

a

b

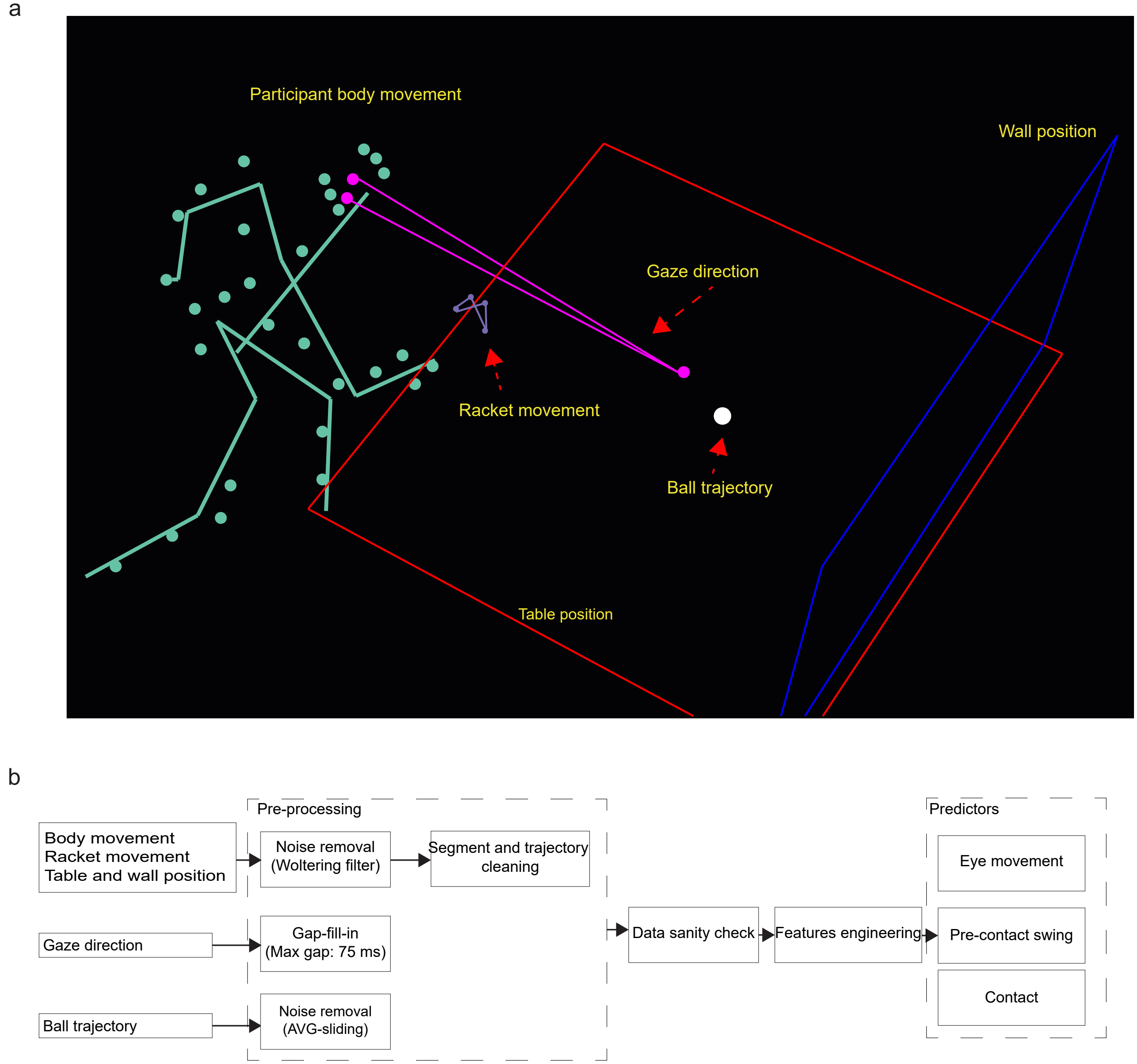

Fig. S2. Modalities measured by wearable eye-tracker and motion capture system. (a) and pre-processing step to extract predictors used in this study (b).

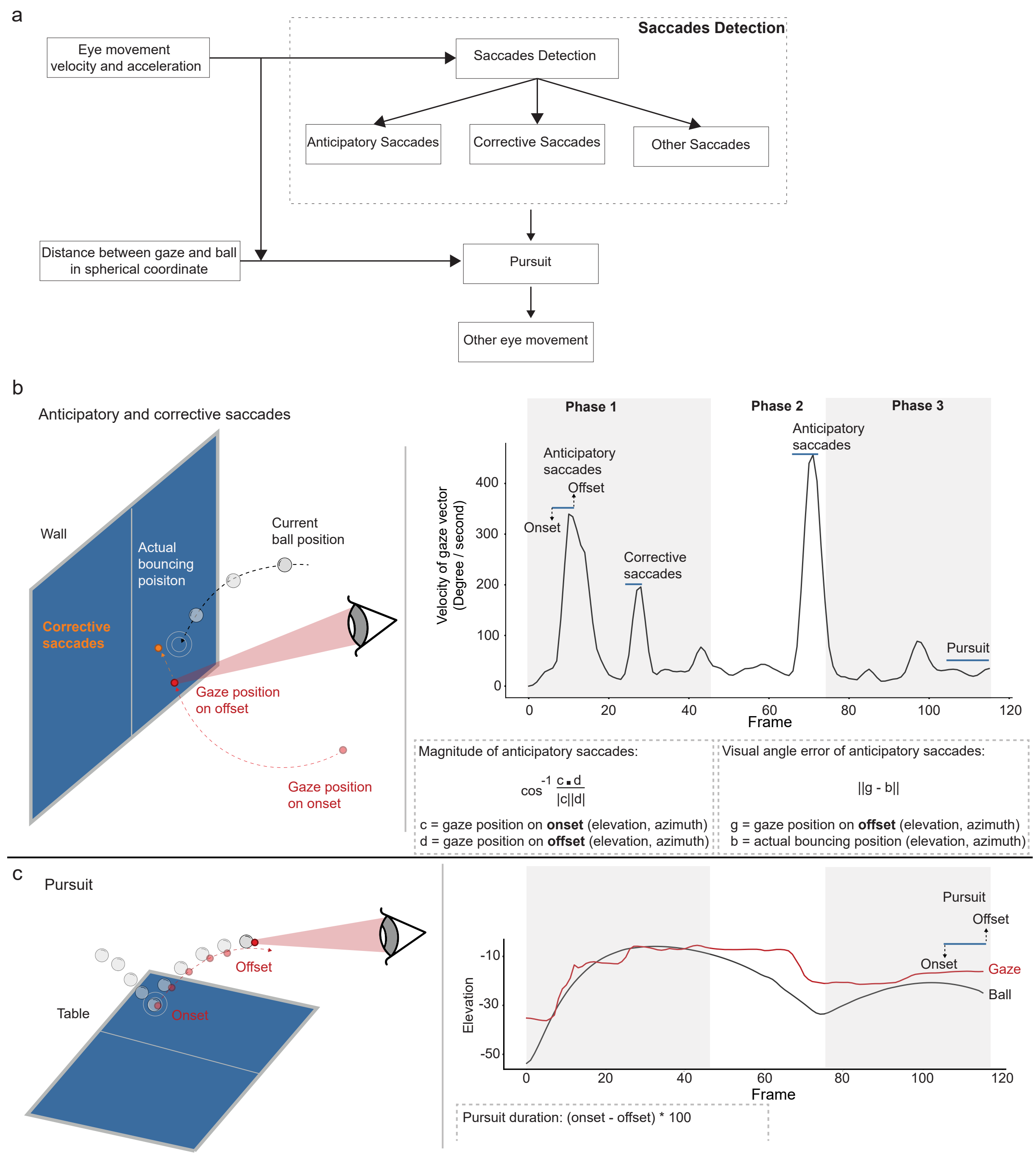

Fig. S3. Distribution of predictors used in Analysis 1. Similarity, difference, and mean metrics were computed from the individual-action task. AS and CS refer to anticipatory and corrective saccades, respectively. Phases are denoted by "P", with "P1" indicating Phase 1.

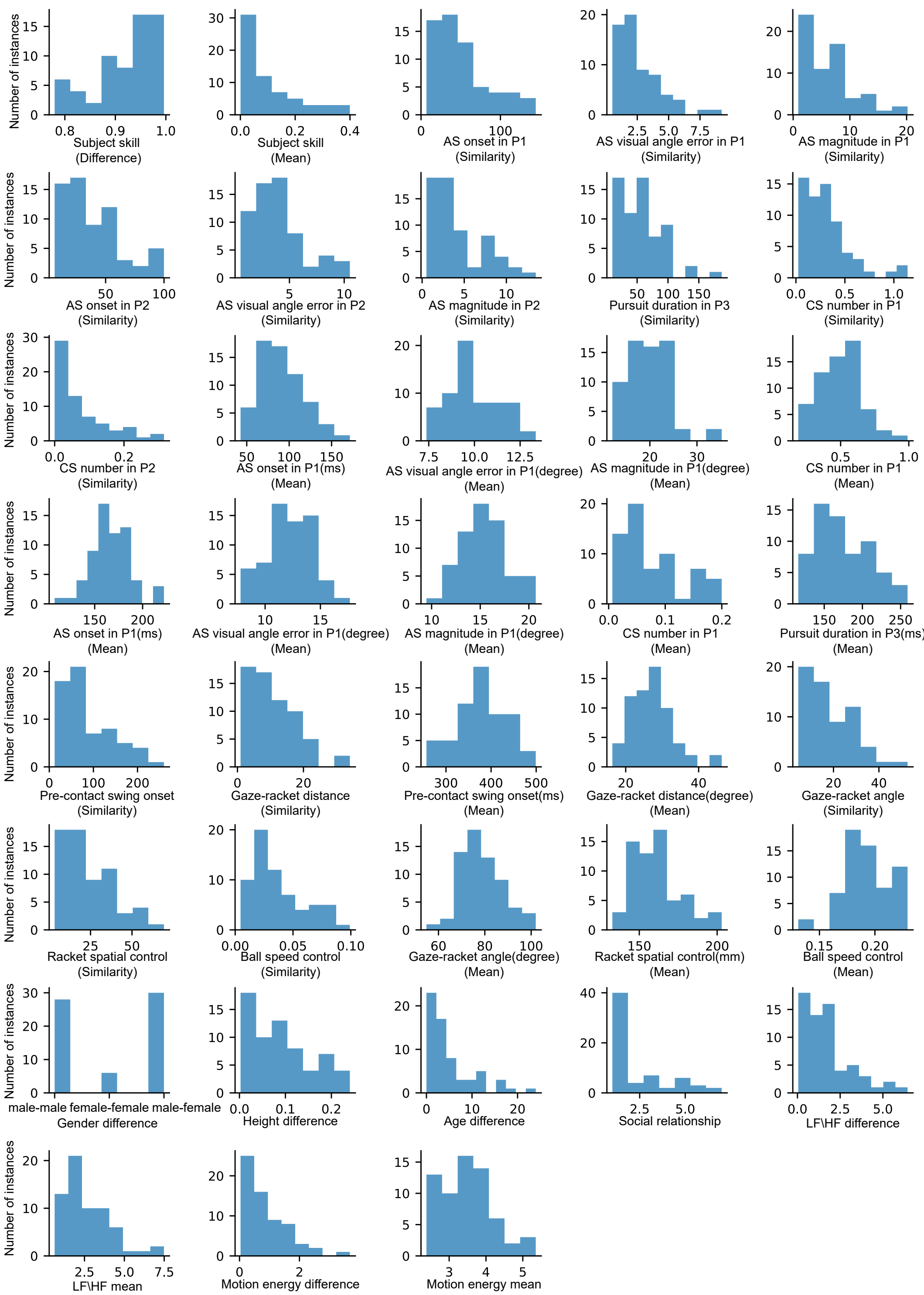

Fig. S4. Distribution of predictors used in Analysis 1. Similarity, difference, and mean metrics were computed from the individual-action task. AS and CS refer to anticipatory and corrective saccades, respectively. Phases are denoted by "P", with "P1" indicating Phase 1.

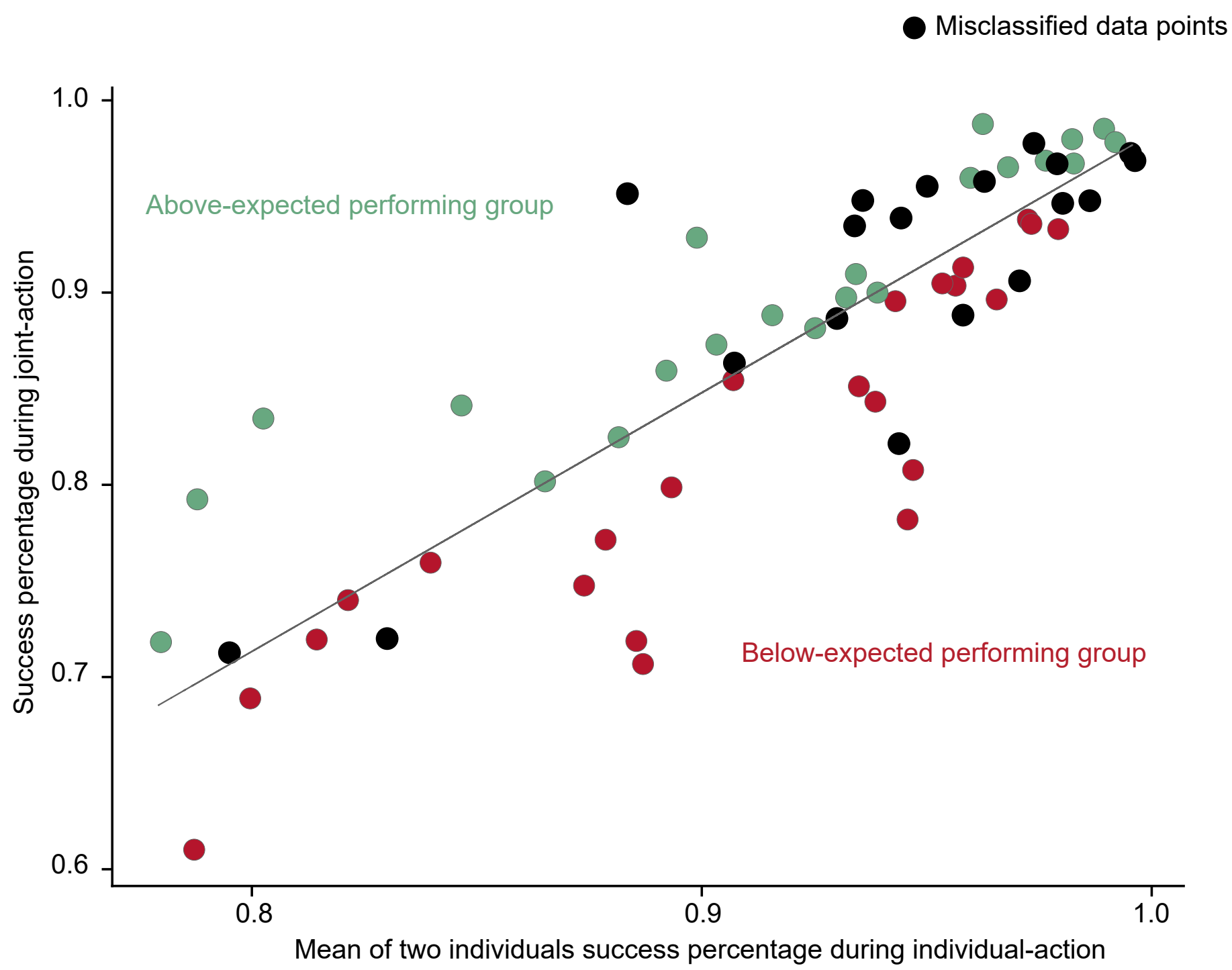

Fig. S5. Prediction results of XGBoost using all predictor categories. Misclassified instances are marked with black circles. Among them, 10 were above-expected cases, and 9 were below-expected cases.

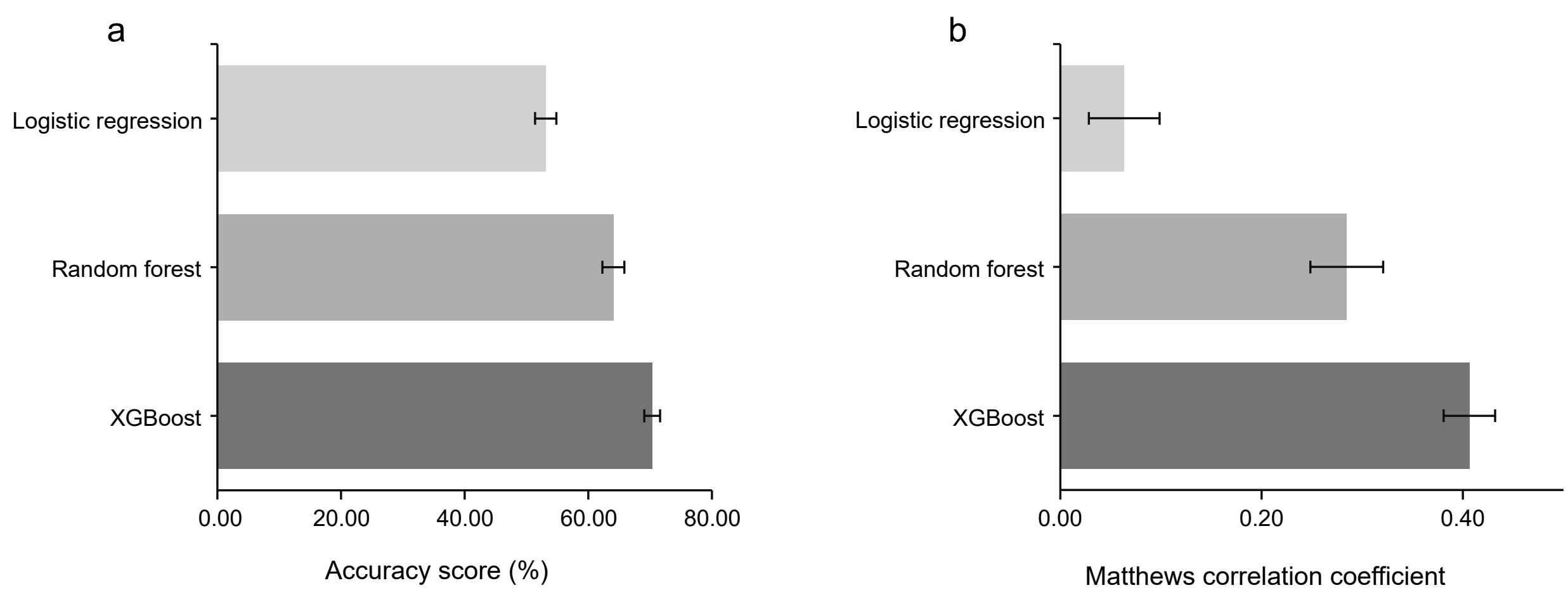

Fig. S6. Performance comparison of multiple agorithms using multimodal predictors (n = 64 observations). Comparison of linear (logistic regression), bagging (random forest), and boosting (XGBoost) algorithms using all predictor categories. The accuracy score reflects the proportion of correctly classified instances out of the total number of cases. In contrast, the Matthews Correlation Coefficient (MCC) incorporates all four elements of the confusion matrix—true positives, true negatives, false positives, and false negatives—offering a balanced and reliable measure of overall classification performance, particularly in imbalanced datasets.

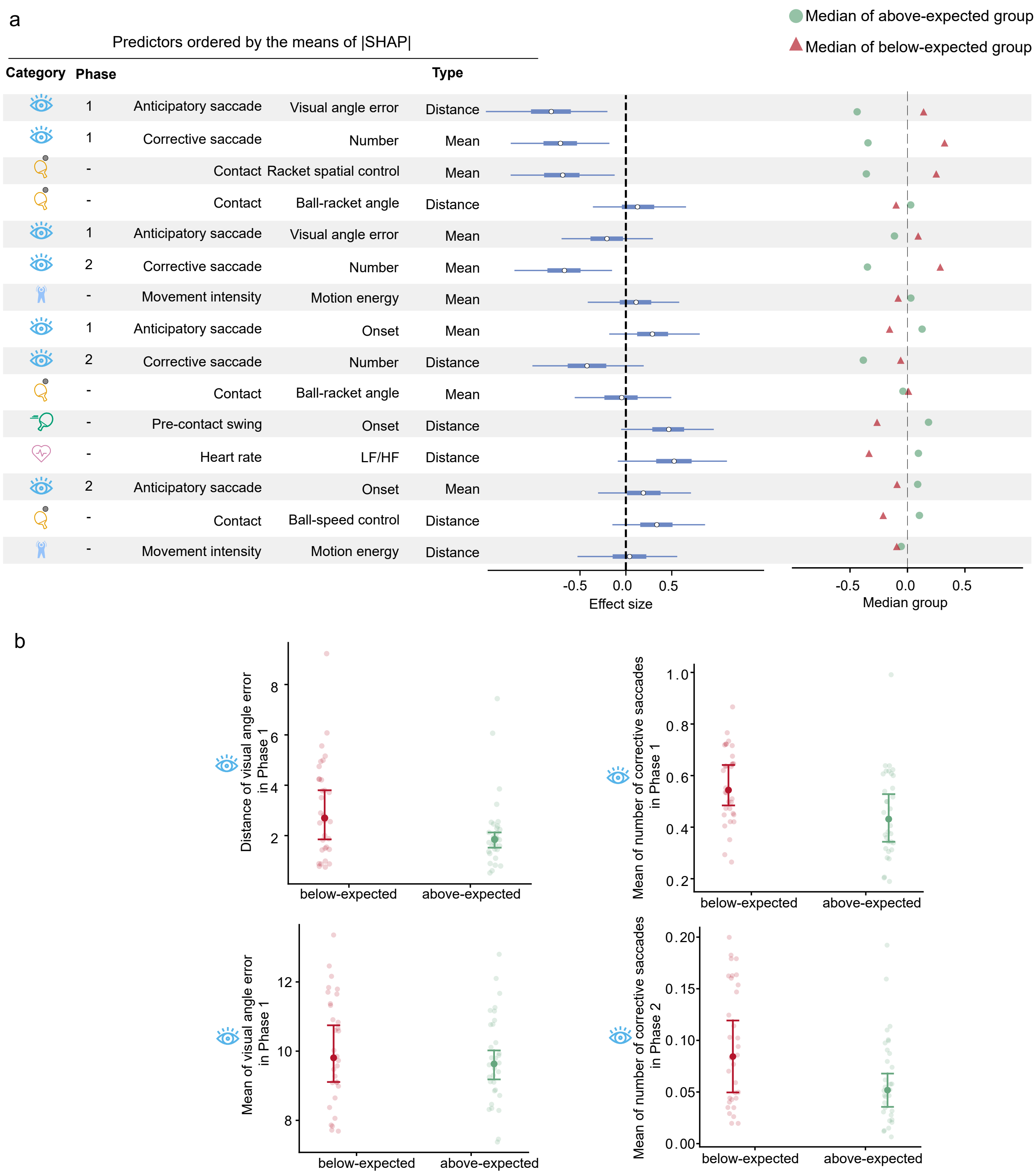

Fig. S7. Bayesian t-test for top-15 predictors (n = 64 observations). (a) Median values (right) and effect sizes (left) of the top 15 predictors ranked by mean absolute SHAP values. (b) Distributions of the significant predictors. Error bars and boxes in the effect size plots represent 95% highest density intervals (HDIs) and the interquartile ranges of the posterior distributions, respectively. Filled circles in distribution plots indicate median values; error bars represent 95% confidence intervals.

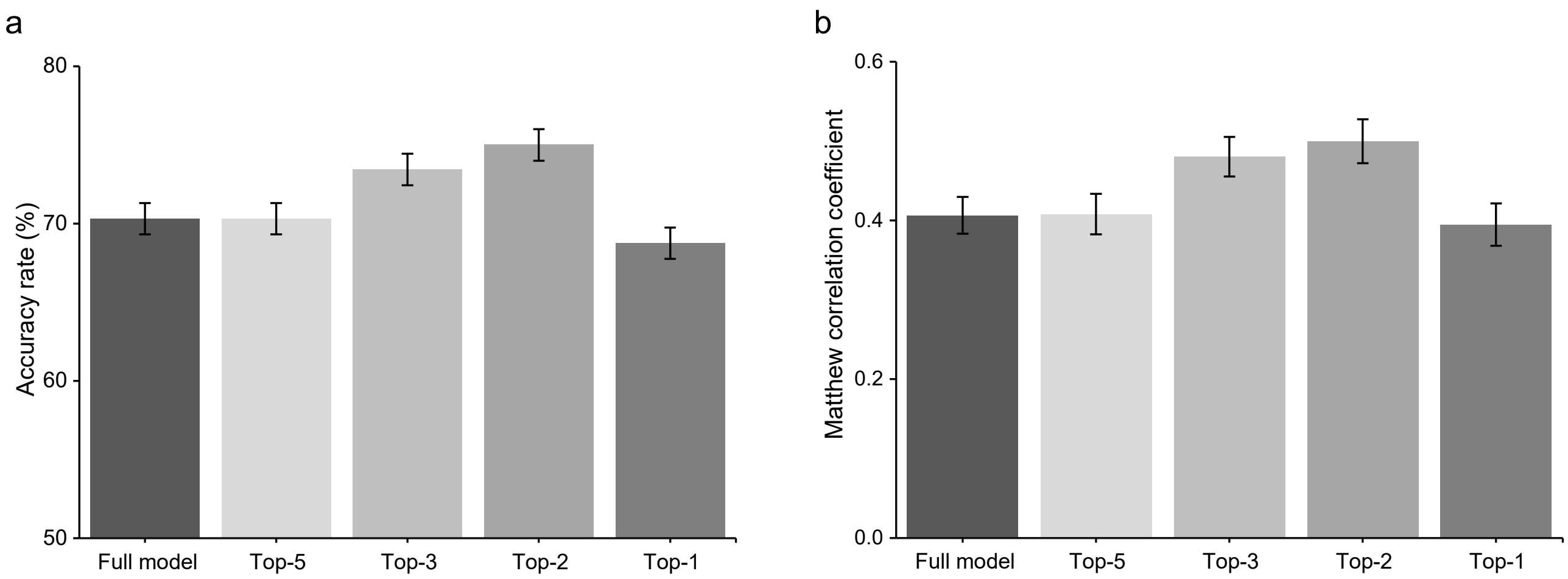

Fig. S8. Accuracy of parsimonious models. (a) Accuracy and (b) Matthews Correlation Coefficient (MCC) scores of the XGBoost model using the top 1, 2, 3, 5, and all predictors. Error bars represent 95% confidence intervals.

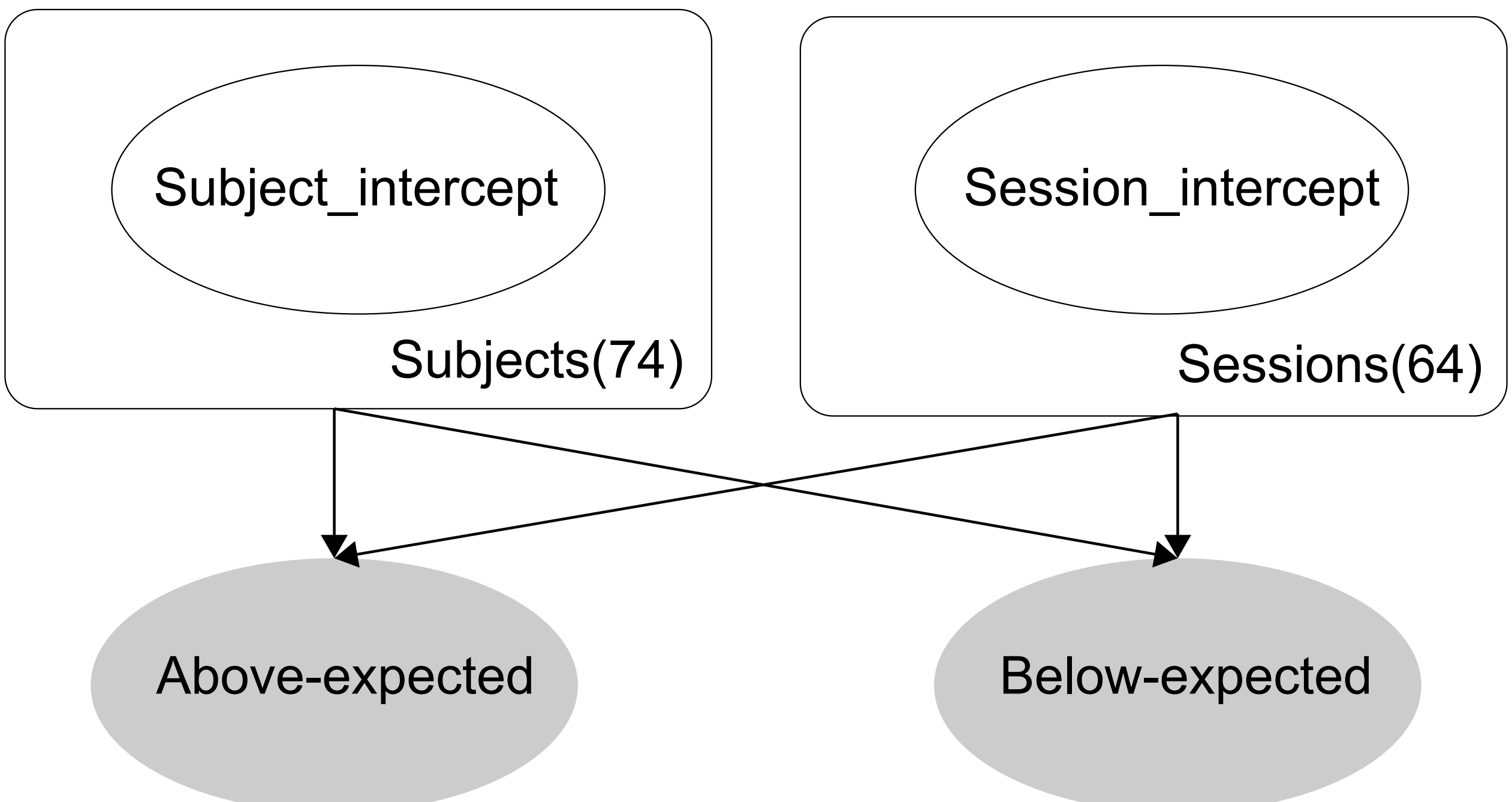

Fig. S9. Hierarchical model of Bayesian estimation. In total, there number of individuals and sessions used in the model were 74 and 64, respectively.

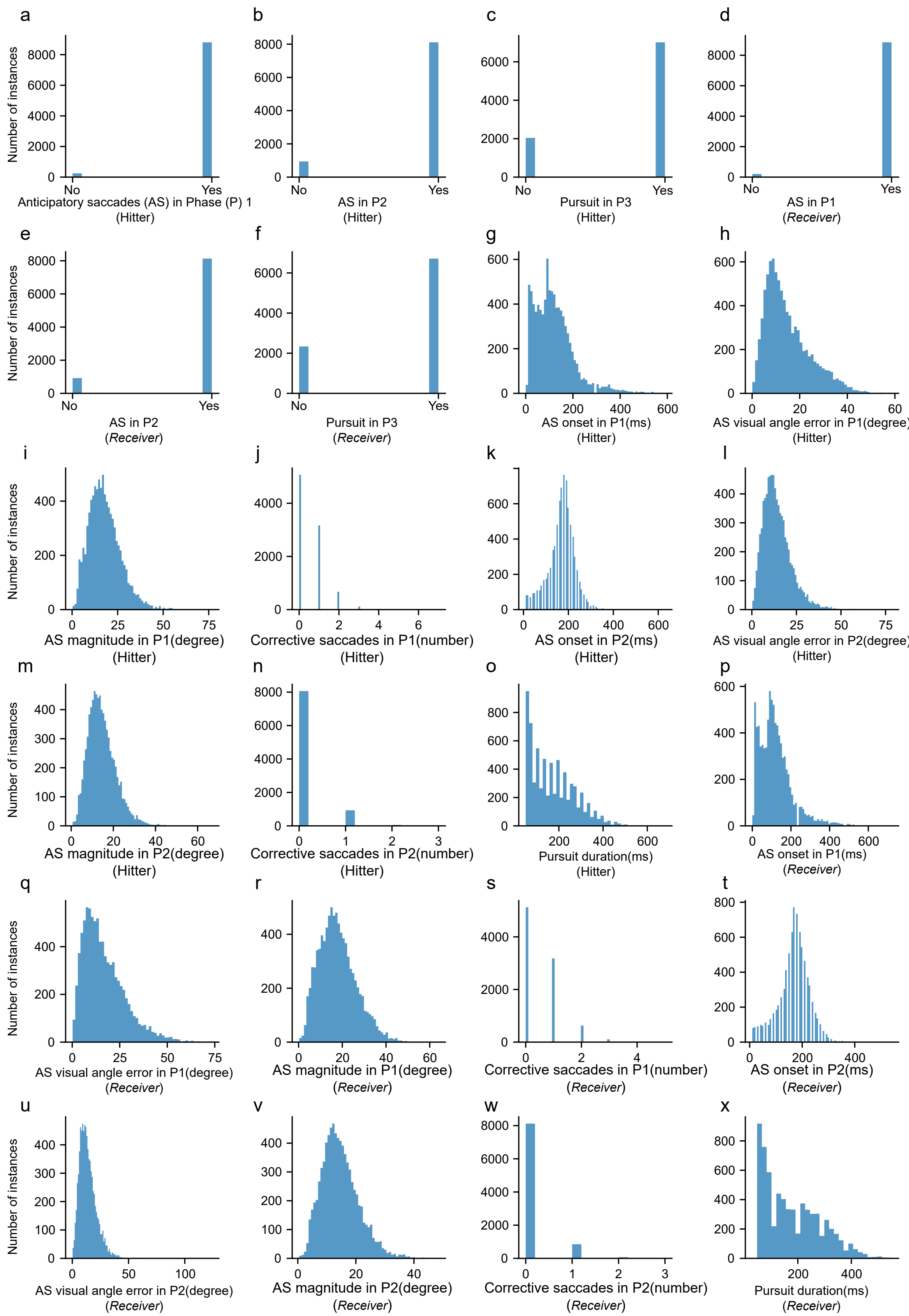

Fig. S10. Distribution of eye-movement predictors used in Analysis 2.

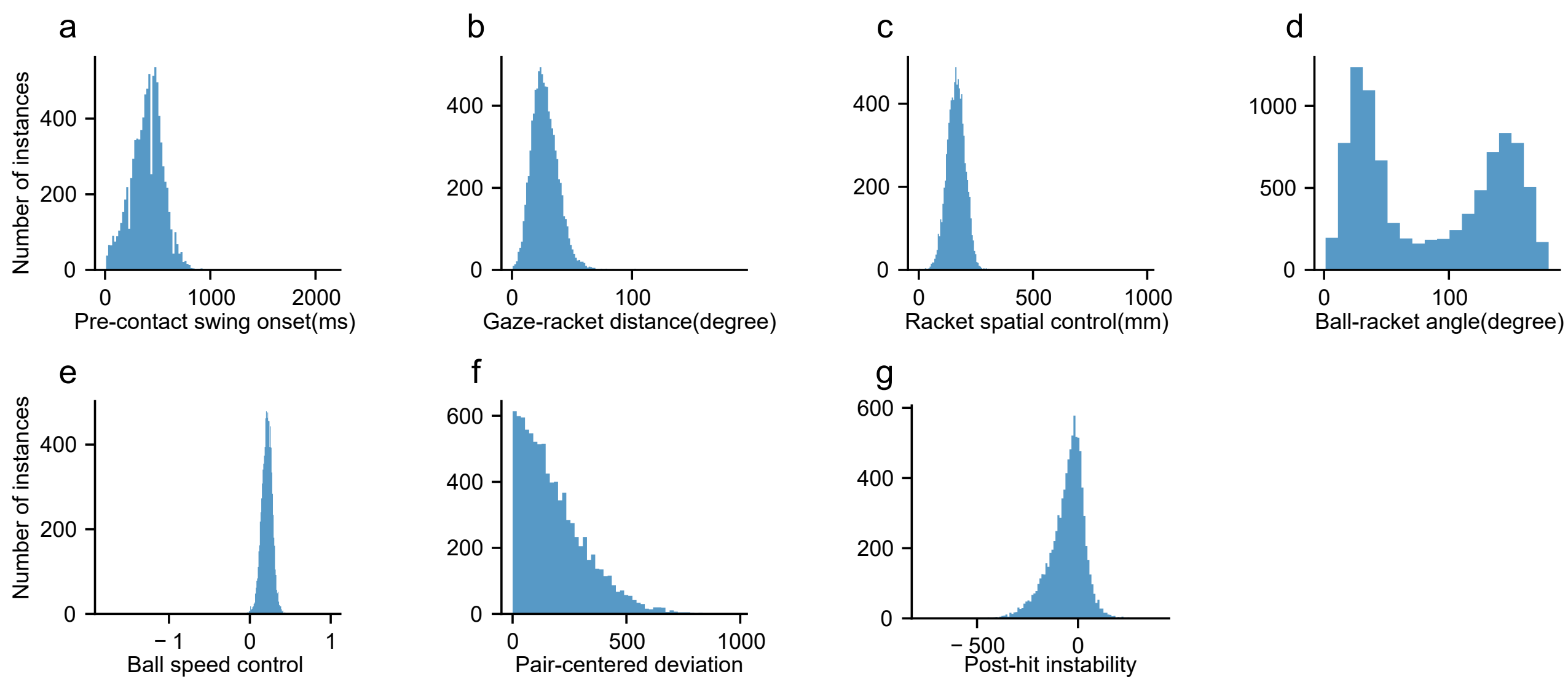

Fig. S11. Distribution of pre-contact, contact, and positional alignment predictors used in Analysis 2.

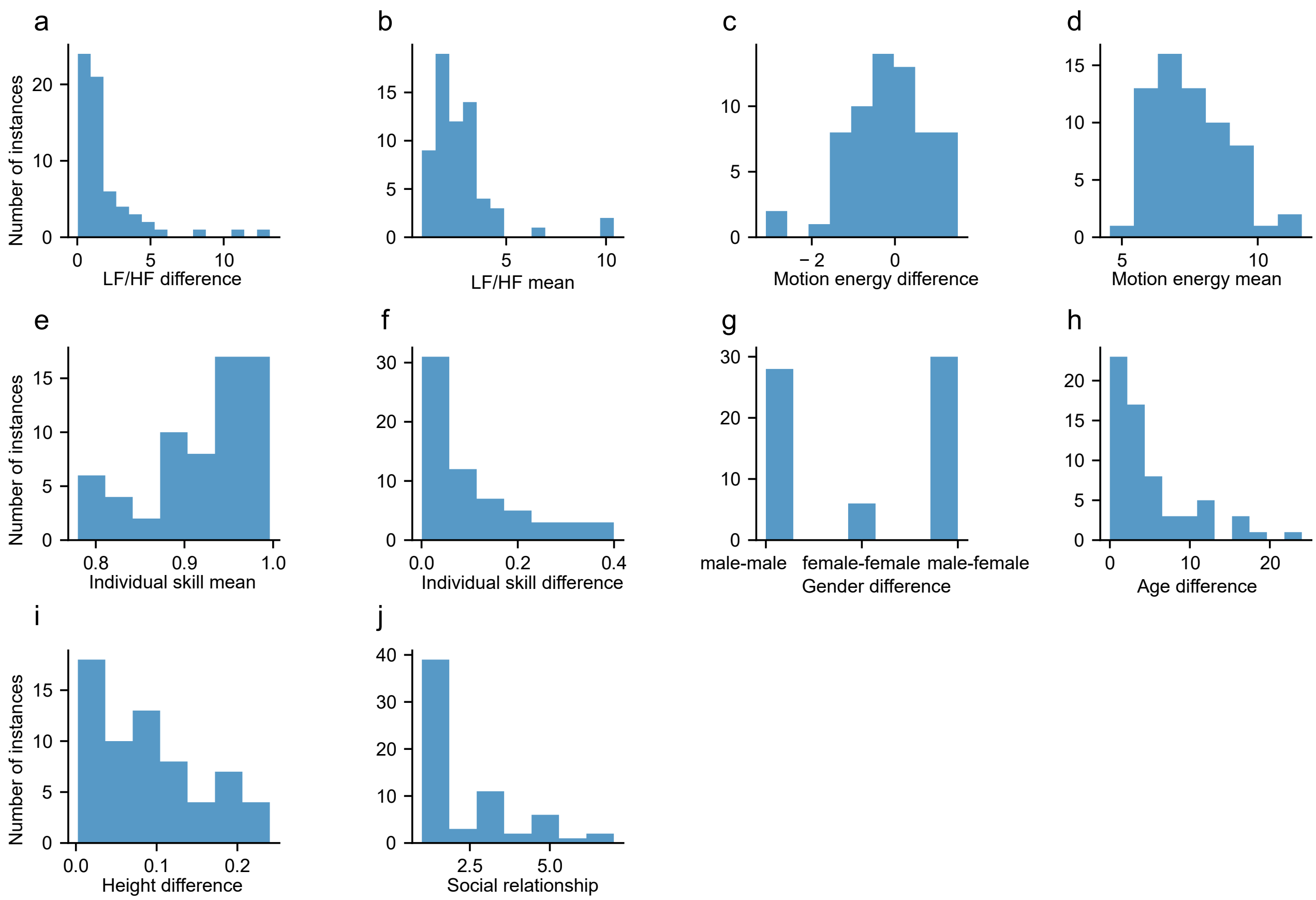

Fig. S12. Distribution of individual skill, demographic, heart rate, and movement intensity used in Analysis 2.

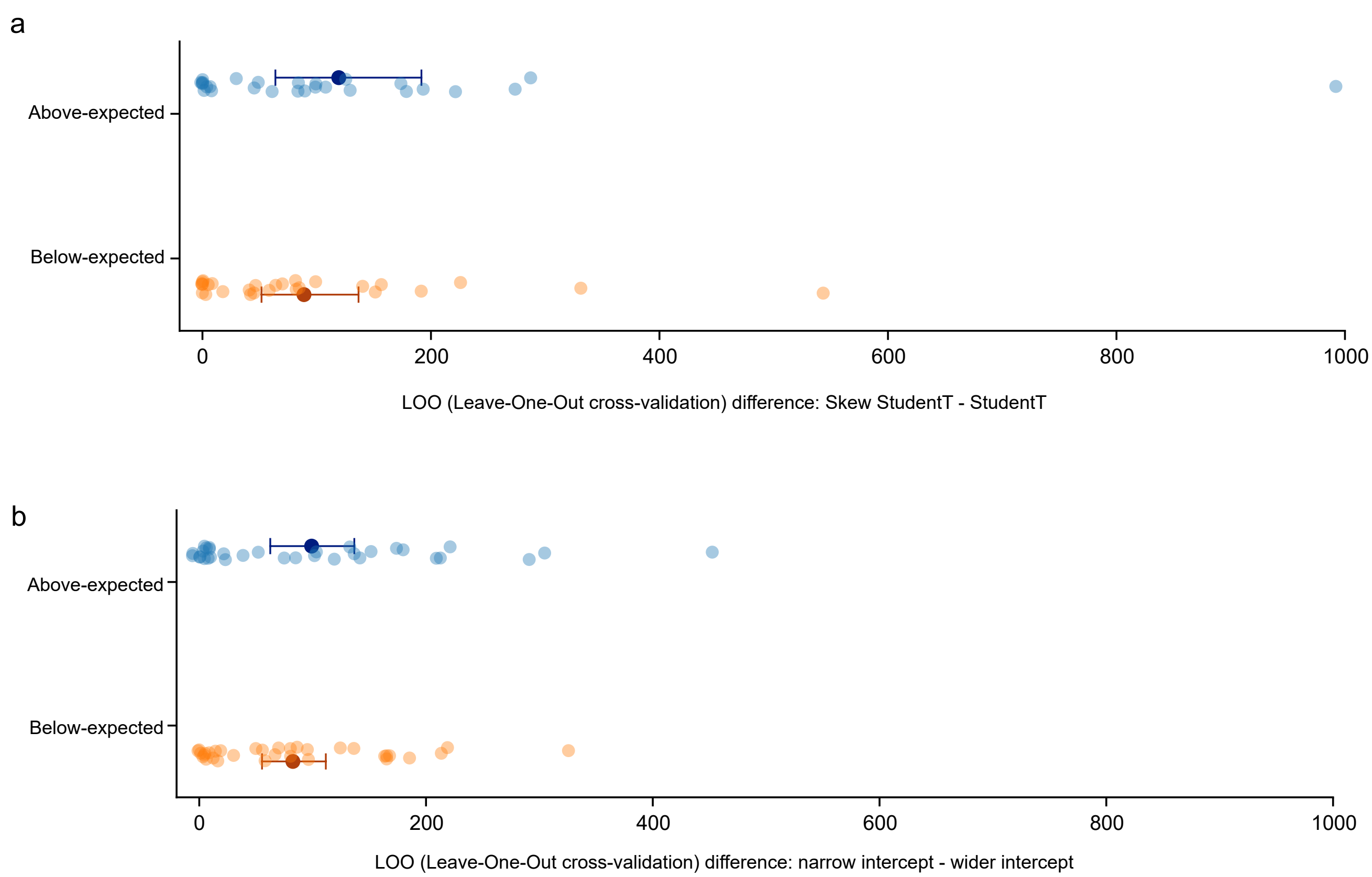

Fig. S13. Sensitivity analyis results. LOO difference between Skew-StudentT and StudentT models (a) and between narrow and wider prior models (b) for subjects and sessions intercepts. Positive values indicate that the Skew-StudentT model has better predictive performance than the StudentT model. Values near zero suggest that the difference is negligible or not statistically meaningful.

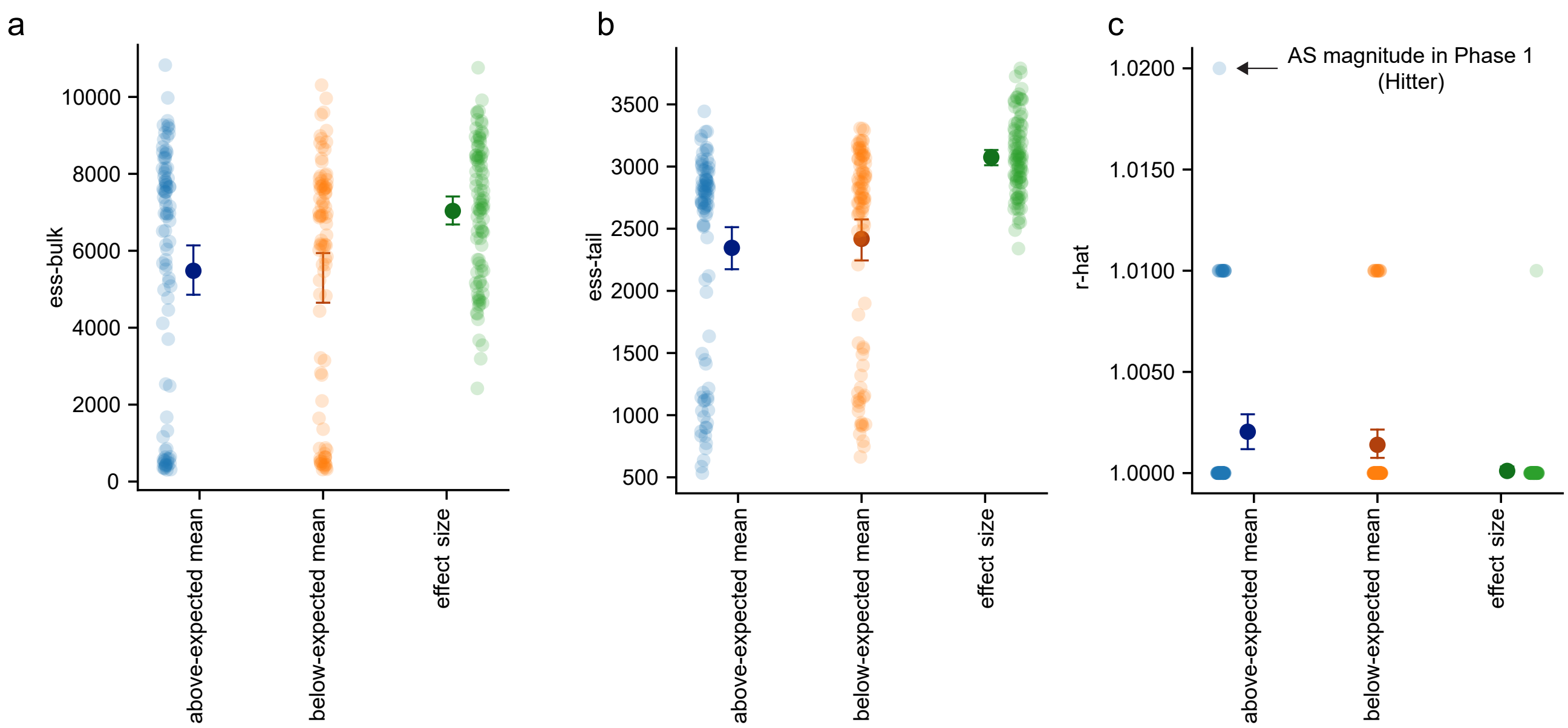

Fig. S14. Results of model convergence test. Distributions of diagnostic statistics from the Bayesian t-test model: (a) Effective sample size for the bulk of the posterior distribution (ess_bulk), (b) Effective sample size for the tails (ess_tail), and (c) Potential scale reduction factor (r_hat). Values of r_hat close to 1 and sufficiently large effective sample sizes (typically > 400) indicate good convergence of the Markov chains.

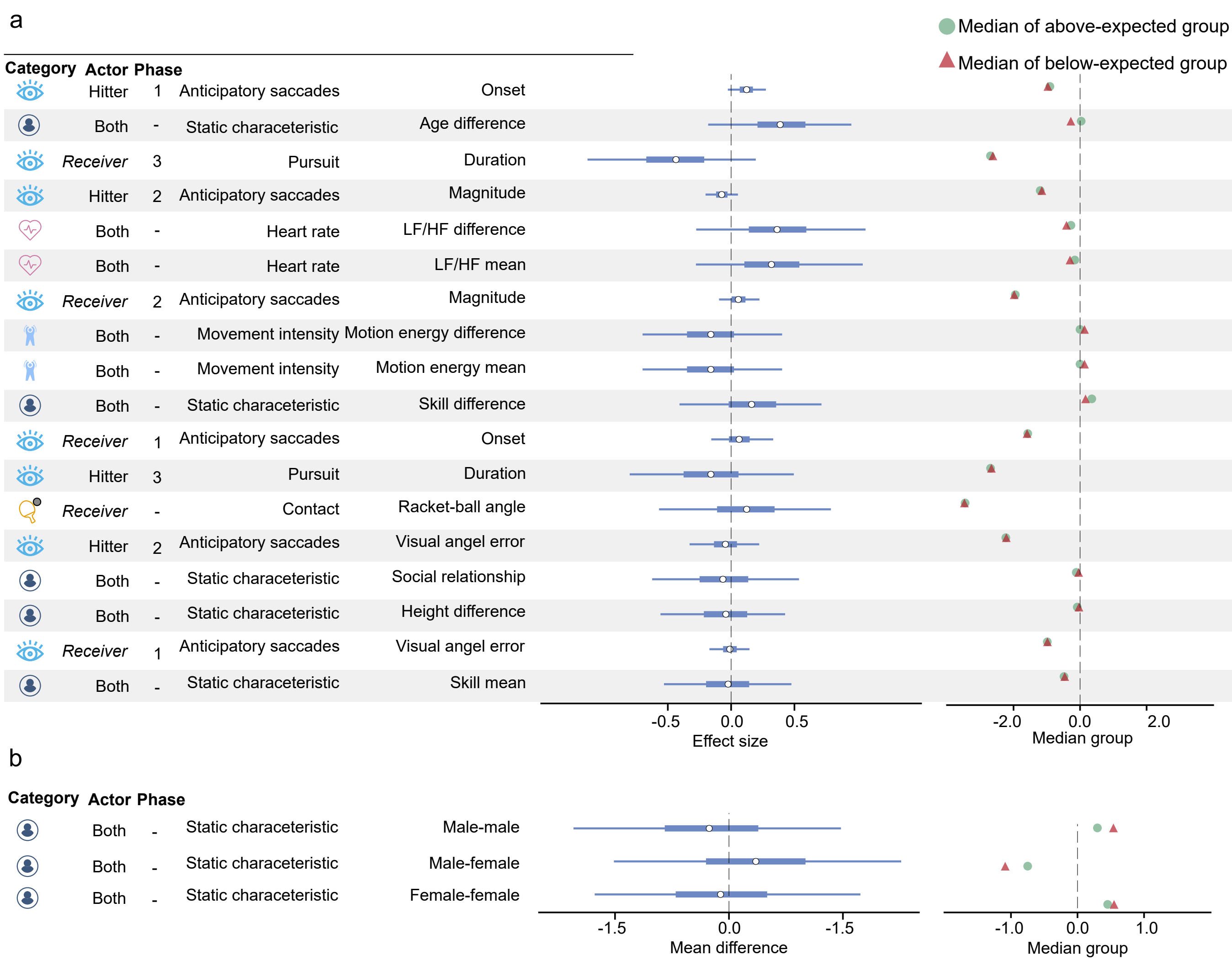

Fig. S15. Predictors showing no significant difference between above- and below-expected groups (n = 64 observations for static features and n = 9056 observations for other features).(**a**) Median predictor values for participants with above- and below-expected outcomes (right), and Bayesian effect sizes (left), defined as posterior mean differences scaled by the pooled standard deviation. (**b**) Median gender values for above- and below-expected groups (right), and corresponding Bayesian mean differences (left). Effect sizes are not shown because many were undefined (NaN) due to mean gender differences being close to zero.

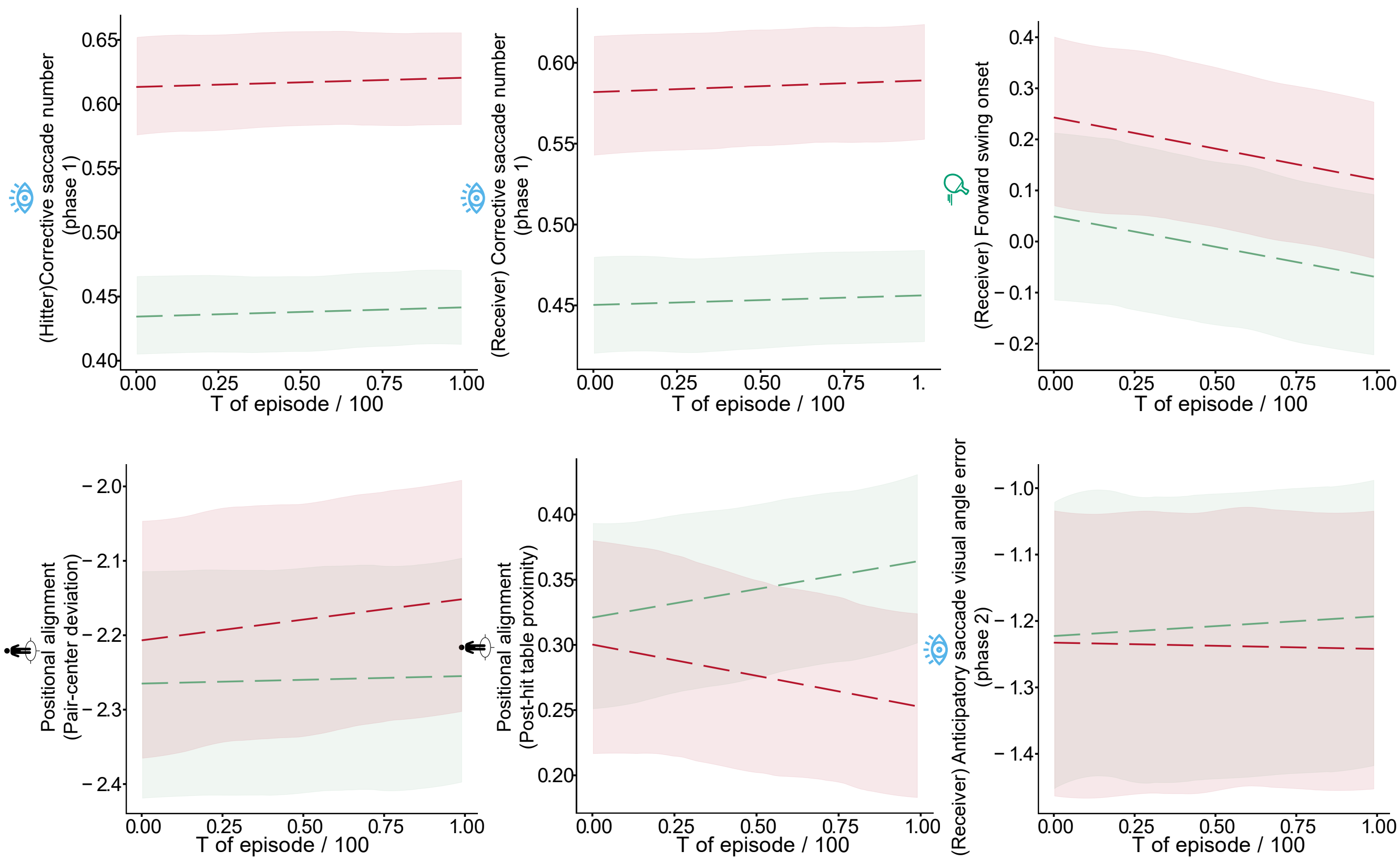

Fig. S16. Visualization of the temporal dynamics of key factors influencing successful coordination, based on posterior estimates (n = 3330 observations). The dashed line indicates the mean trend, while the shaded area represents the 95% Highest Density Interval (HDI).

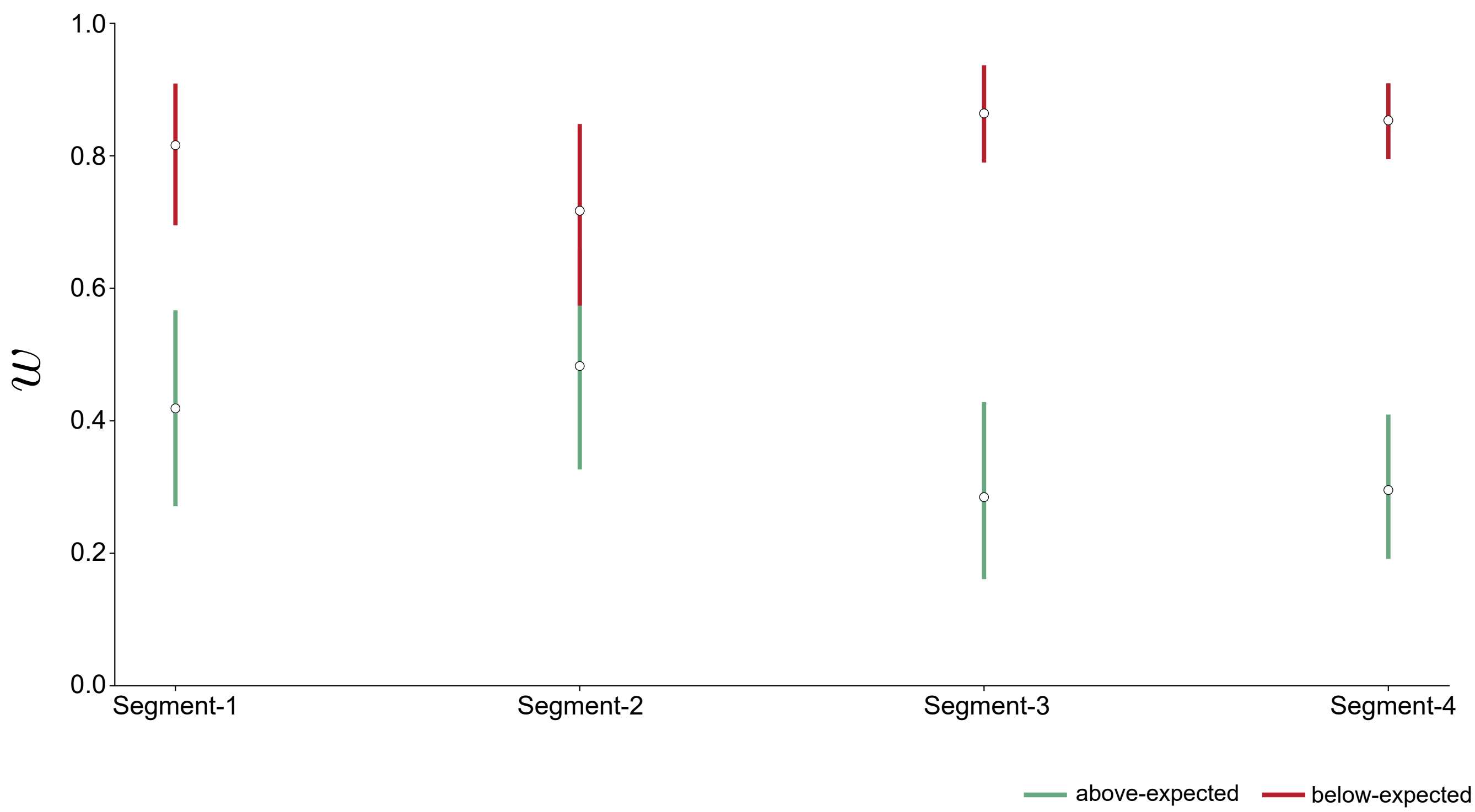

Fig. S17. **Median sensory-evidence weights (circles) with 95% confidence intervals (error bars) for each segment (n = 3330 observations).**

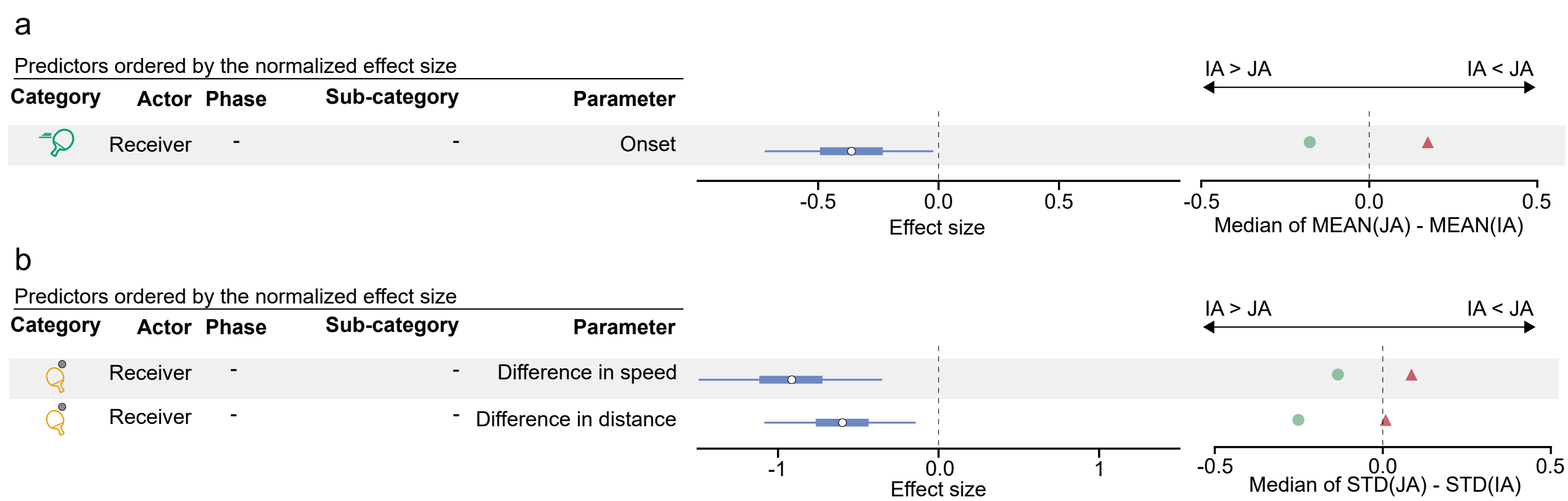

Fig. S18. Comparison of individual behavior between individual and joint-action tasks (n = 64 observations). (**a**) Median predictor values for the above- and below-expected groups (right), and Bayesian effect sizes (left), defined as the posterior mean differences between groups expressed in standard deviation units. Differences in the mean (**b**), and differences in the standard deviation (SD) (**c**), of predictor values between individual-action (IA) and joint-action (JA) tasks. Error bars and boxes in the effect size plots represent 95% highest density intervals (HDIs) and the interquartile ranges of the posterior distributions, respectively.

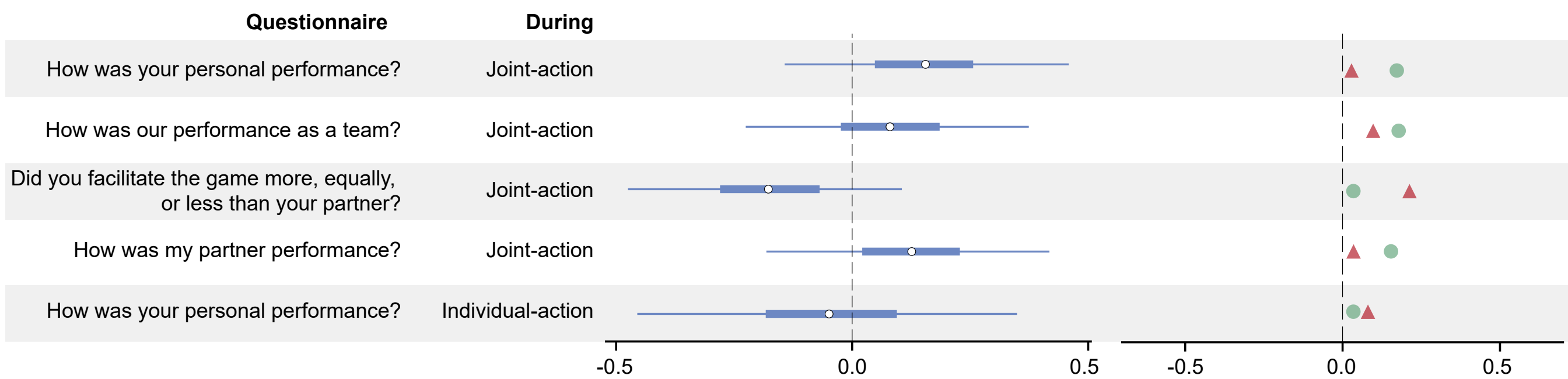

Fig. S19. Bayesian t-test results for self-assessment predictors (n = 64 observations). (**a**) Median predictor values for the above- and below-expected groups (right), and Bayesian effect sizes (left), defined as the posterior mean differences between groups expressed in standard deviation units. Error bars and boxes in the effect size plots represent 95% highest density intervals (HDIs) and the interquartile ranges of the posterior distributions, respectively.

Table S1. Variance inflation factors (VIFs) of the predictor predictors used in the Analysis 1. Higher VIF values indicate greater multicollinearity

Predictor name	Type	Variance Inflation
Subject skill	Mean	26.20
Subject skill	Difference	17.69
Corrective saccade in P2	Mean	13.60
Pre-contact swing onset	Mean	13.33
Corrective saccade in P1	Mean	8.12
Corrective saccade in P2	Difference	6.76
Visual angle error of anticipatory saccade in P2	Mean	6.72
Pursuit duration in P3	Mean	6.64
Magnitude of anticipatory saccade in P1	Mean	6.19
Ball speed control	Mean	6.11
Onset of anticipatory saccade in P2	Mean	5.64
Onset of anticipatory saccade in P1	Mean	5.32
Racket spatial control	Difference	5.29
Magnitude of anticipatory saccade in P2	Mean	5.09
Gaze racket distance	Mean	4.24
Pre-contact swing onset	Difference	4.23
Magnitude of anticipatory saccade in P1	Difference	4.16
Onset of anticipatory saccade in P1	Difference	4.16
LF/HF	Mean	4.11
Gaze racket angle	Difference	4.04
Corrective saccade in P1	Difference	4.04
LF/HF	Difference	3.96
Motion energy	Mean	3.95
Magnitude of anticipatory saccade in P2	Difference	3.73
Racket spatial control	Mean	3.61
Visual angle error of anticipatory saccade n P1	Mean	3.48
Gaze racket distance	Difference	3.48
Pursuit duration in P3	Difference	3.28
Motion energy	Difference	3.09
Visual angle error of anticipatory saccade in P1	Difference	3.07
Gender	Difference	3.00
Ball speed control	Difference	2.94
Onset of anticipatory saccade in P2	Difference	2.81
Visual angle error of anticipatory saccade in P2	Difference	2.69
Height	Difference	2.60
Relationship	Difference	2.36
Gaze racket angle	Mean	2.34
Age	Difference	2.23

Table S2. Results of the PCA analysis (n = 64 observations). $N_{component}$ represents the number of latent variables extracted from the original set of predictors.

N_component	ACC	MCC	AUC_PR
2	57.81%	0.17	0.52
5	53.13%	0.07	0.53
10	51.56%	0.03	0.55
15	56.25%	0.13	0.60

Table S3. Overview of the experimental sessions, showing the number of participants recruited in each session and the total number of dyads formed. Sessions with three participants yielded three unique dyads, whereas sessions with two participants yielded one dyad.

#	Session	Total number of participants	Total number of dyads	Excluded dyads	Retained dyads
1	2022-11-08_A	3	3	0	3
2	2022-11-09_A	3	3	0	3
3	2022-11-14_M	3	3	0	3
4	2022-11-15_A	3	3	0	3
5	2022-11-15_M	3	3	0	3
6	2022-11-17_M	2	1	0	1
7	2022-11-21_A	3	3	0	3
8	2022-11-24_M	3	3	0	3
9	2022-11-28_A	3	3	0	3
10	2022-11-29_A	3	3	3	0
11	2022-11-30_A	3	3	2	1
12	2022-12-01_A	3	3	0	3
13	2022-12-05_M	3	3	0	3
14	2022-12-07_A	2	1	0	1
15	2022-12-07_M	2	1	1	0
16	2022-12-08_A	3	3	0	3
17	2022-12-08_M	3	3	0	3
18	2022-12-19_A	3	3	0	3
19	2022-12-20_A	2	1	1	0
20	2022-12-21_M	3	3	0	3
21	2023-01-03_A	2	1	0	1
22	2023-01-16_A	3	3	0	3
23	2023-01-17_A	2	1	1	0
24	2023-01-19_A	2	1	1	0
25	2023-01-26_M	2	1	0	1
26	2023-02-06_A	2	1	0	1
27	2023-02-07_A	3	3	0	3
28	2023-02-08_A	3	3	3	0
29	2023-02-15_A	2	1	0	1
30	2023-02-15_M	3	3	2	1
31	2023-02-27_A	2	1	0	1
32	2023-03-09_A	3	3	2	1
33	2023-03-15_A	3	3	0	3
34	2023-03-16_A	3	3	0	3
	Total	91	80	16	64

Table S4. Sessions per participant. For each participant, the number of sessions and the corresponding session identifiers are listed; the count reflects how many experimental sessions the participant attended.

No.	Participant ID	No. of sessions	Sessions
1	SE001A	2	2022-11-08_A_T05, 2022-11-08_A_T06
2	SE001B	2	2022-11-08_A_T02, 2022-11-08_A_T05
3	SE001C	2	2022-11-08_A_T02, 2022-11-08_A_T06
4	SE002A	2	2022-11-09_A_T01, 2022-11-09_A_T06
5	SE002B	2	2022-11-09_A_T05, 2022-11-09_A_T06
6	SE002C	2	2022-11-09_A_T01, 2022-11-09_A_T05
7	SE003A	2	2022-11-14_M_T03, 2022-11-14_M_T04
8	SE003B	2	2022-11-14_M_T04, 2022-11-14_M_T05
9	SE003C	2	2022-11-14_M_T03, 2022-11-14_M_T05
10	SE004A	2	2022-11-15_M_T05, 2022-11-15_M_T06
11	SE004B	2	2022-11-15_M_T02, 2022-11-15_M_T06
12	SE004C	2	2022-11-15_M_T02, 2022-11-15_M_T05
13	SE005A	2	2022-11-15_A_T01, 2022-11-15_A_T03
14	SE005B	2	2022-11-15_A_T02, 2022-11-15_A_T03
15	SE005C	2	2022-11-15_A_T01, 2022-11-15_A_T02
16	SE006A	1	2022-11-17_M_T05
17	SE006C	1	2022-11-17_M_T05
18	SE007A	2	2022-11-21_A_T01B, 2022-11-21_A_T05
19	SE007B	2	2022-11-21_A_T02, 2022-11-21_A_T05
20	SE007C	2	2022-11-21_A_T01B, 2022-11-21_A_T02
21	SE008A	2	2022-11-24_M_T02, 2022-11-24_M_T05
22	SE008B	2	2022-11-24_M_T04, 2022-11-24_M_T05
23	SE008C	2	2022-11-24_M_T02, 2022-11-24_M_T04
24	SE009A	2	2022-11-28_A_T05, 2022-11-28_A_T06
25	SE009B	2	2022-11-28_A_T01, 2022-11-28_A_T06
26	SE009C	2	2022-11-28_A_T01, 2022-11-28_A_T05
27	SE011B	1	2022-11-30_A_T06
28	SE011C	1	2022-11-30_A_T06
29	SE012A	2	2022-12-01_A_T02, 2022-12-01_A_T06
30	SE012B	2	2022-12-01_A_T05, 2022-12-01_A_T06
31	SE012C	2	2022-12-01_A_T02, 2022-12-01_A_T05
32	SE013A	2	2022-12-05_M_T01, 2022-12-05_M_T05
33	SE013B	2	2022-12-05_M_T02, 2022-12-05_M_T05
34	SE013C	2	2022-12-05_M_T01, 2022-12-05_M_T02
35	SE016A	1	2022-12-07_A_T05B
36	SE016B	1	2022-12-07_A_T05B
37	SE017A	2	2022-12-08_M_T02, 2022-12-08_M_T04
38	SE017B	2	2022-12-08_M_T02, 2022-12-08_M_T06
39	SE017C	2	2022-12-08_M_T04, 2022-12-08_M_T06
40	SE018A	2	2022-12-08_A_T02, 2022-12-08_A_T03
41	SE018B	2	2022-12-08_A_T03, 2022-12-08_A_T04A
42	SE018C	2	2022-12-08_A_T02, 2022-12-08_A_T04A
43	SE019A	2	2022-12-19_A_T01B, 2022-12-19_A_T04B
44	SE019B	2	2022-12-19_A_T01B, 2022-12-19_A_T06
45	SE019C	2	2022-12-19_A_T04B, 2022-12-19_A_T06
46	SE021A	2	2022-12-21_M_T01, 2022-12-21_M_T06
47	SE021B	2	2022-12-21_M_T05, 2022-12-21_M_T06
48	SE021C	2	2022-12-21_M_T01, 2022-12-21_M_T05
49	SE022A	1	2023-01-03_A_T04
50	SE022B	1	2023-01-03_A_T04
51	SE023A	2	2023-01-16_A_T02, 2023-01-16_A_T04
52	SE023B	2	2023-01-16_A_T01, 2023-01-16_A_T02
53	SE023C	2	2023-01-16_A_T01, 2023-01-16_A_T04
54	SE026A	1	2023-01-26_M_T01

Continued on next page

Table S4 – continued from previous page

No.	Participant ID	No. of sessions	Sessions
55	SE026B	1	2023-01-26_M_T01
56	SE027A	1	2023-02-06_A_T03
57	SE027B	1	2023-02-06_A_T03
58	SE028A	2	2023-02-07_A_T03, 2023-02-07_A_T05
59	SE028B	2	2023-02-07_A_T04, 2023-02-07_A_T05
60	SE028C	2	2023-02-07_A_T03, 2023-02-07_A_T04
61	SE030A	1	2023-02-15_M_T01
62	SE030C	1	2023-02-15_M_T01
63	SE031A	1	2023-02-15_A_T03
64	SE031B	1	2023-02-15_A_T03
65	SE032A	1	2023-02-27_A_T03
66	SE032B	1	2023-02-27_A_T03
67	SE033B	1	2023-03-09_A_T01
68	SE033C	1	2023-03-09_A_T01
69	SE034A	2	2023-03-15_A_T03, 2023-03-15_A_T04
70	SE034B	2	2023-03-15_A_T03, 2023-03-15_A_T05
71	SE034C	2	2023-03-15_A_T04, 2023-03-15_A_T05
72	SE035A	2	2023-03-16_A_T01, 2023-03-16_A_T02
73	SE035B	2	2023-03-16_A_T01, 2023-03-16_A_T04
74	SE035C	2	2023-03-16_A_T02, 2023-03-16_A_T04

Table S5. Optimized XGBoost hyperparameters for each feature category. The table lists the selected values for learning_rate, subsample, max_depth, alpha, min_child_weight, and max_delta_step used in model training across different input feature sets.

Features category	learning_rate	subsample	max_depth	alpha	min_child_weight	max_delta_step
Full-model	0.020	1.000	3	0.050	3	3
Eye-movement	0.030	1.000	3	0.050	3	3
Forward swing	0.010	1.000	3	0.050	1	1
Impact	0.032	0.744	3	0.074	3	2
Heart rate	0.010	0.950	3	0.046	3	3
Motion energy	0.015	0.506	4	0.263	3	1
Individual characteristic	0.021	0.550	4	0.029	3	2

Table S6. Hyperparameters used to sample the model parameters vary for each model, taking into account the convergence of the estimation process.

Parameters	Values (GLM)	Values (T-Test and Generative)
Number of samples	1000	1000
Number of tunes	500	2000
Number of chains	4	4
Number of core	4	4
Target acceptance	0.95	0.99

Movie S1. An Illustration of Eye and Body Movement Data from the Individual-Action Task. Dots represent motion-capture markers connected by lines indicating joints. The pink line illustrates the gaze direction captured using wearable eye-trackers.

Movie S2. An Illustration of Eye and Body Movement Data from the Joint-Action Task. Dots represent motion-capture markers connected by lines indicating joints. Different colors of dots and lines indicate different individual IDs. The pink line illustrates the gaze direction captured using wearable eye-trackers.

References

1. D Rajput, WJ Wang, CC Chen, Evaluation of a decided sample size in machine learning applications. *BMC bioinformatics* **24**, 48 (2023).
2. MD Hoffman, A Gelman, , et al., The no-u-turn sampler: adaptively setting path lengths in hamiltonian monte carlo. *J. Mach. Learn. Res.* **15**, 1593–1623 (2014).
3. JO Friedrich, NK Adhikari, J Beyene, The ratio of means method as an alternative to mean differences for analyzing continuous outcome variables in meta-analysis: a simulation study. *BMC medical research methodology* **8**, 32 (2008).
