# Supplementary material for: Successful coordination emerges from aligned self-predictions in a dynamic motor interaction
Source: Commun Psychol. 2026 Aug 20;4:119. doi: 10.1038/s44271-026-00523-7 (PMC13493972; doi:10.1038/s44271-026-00523-7)
Supplement: Supplementary file 3 [file 44271_2026_523_MOESM3_ESM.pdf]

Description of Additional Supplementary Files

File name: Supplementary Movie S1

Description: An Illustration of Eye and Body Movement Data from the Individual-Action Task. Dots represent motion-capture markers connected by lines indicating joints. The pink line illustrates the gaze direction captured using wearable eye-trackers.

File name: Supplementary Movie S2

Description: An Illustration of Eye and Body Movement Data from the Joint-Action Task. Dots represent motion-capture markers connected by lines indicating joints. Different colors of dots and lines indicate different individual IDs. The pink line illustrates the gaze direction captured using wearable eye-trackers.
